# Chemosensory Neurons Modulate the Response to Oomycete Recognition in *Caenorhabditis elegans*

**DOI:** 10.1016/j.celrep.2020.108604

**Published:** 2021-01-12

**Authors:** Michael K. Fasseas, Manish Grover, Florence Drury, Clara L. Essmann, Eva Kaulich, William R. Schafer, Michalis Barkoulas

**Affiliations:** 1 Department of Life Sciences, Imperial College, London SW7 2AZ, UK; 2 MRC Laboratory of Molecular Biology, Cambridge CB2 0QH, UK

**Keywords:** oomycete, pathogen recognition, innate immunity, *chitinase-like* genes, *pals*, *Myzocytiopsis humicola*, *Caenorhabditis elegans*, hypodermis, cuticle, cross-tissue signaling

## Abstract

Understanding how animals detect and respond to pathogen threats is central to dissecting mechanisms of host immunity. The oomycetes represent a diverse eukaryotic group infecting various hosts from nematodes to humans. We have previously shown that *Caenorhabditis elegans* mounts a defense response consisting of the induction of *chitinase-like* (*chil*) genes in the epidermis to combat infection by its natural oomycete pathogen *Myzocytiopsis humicola*. We provide here evidence that *C. elegans* can sense the oomycete by detecting an innocuous extract derived from animals infected with *M. humicola*. The oomycete recognition response (ORR) leads to changes in the cuticle and reduction in pathogen attachment, thereby increasing animal survival. We also show that TAX-2/TAX-4 function in chemosensory neurons is required for the induction of *chil-27* in the epidermis in response to extract exposure. Our findings highlight that neuron-to-epidermis communication may shape responses to oomycete recognition in animal hosts.

## Highlights

• *C. elegans* senses its natural oomycete pathogen *M. humicola* without infection
• Exposure to a pathogen extract triggers an oomycete recognition response
• Upon pathogen detection, *C. elegans* resists infection through changes in the cuticle
• The response involves signaling between sensory neurons and the epidermis

## Graphical Abstract

**Figure:**
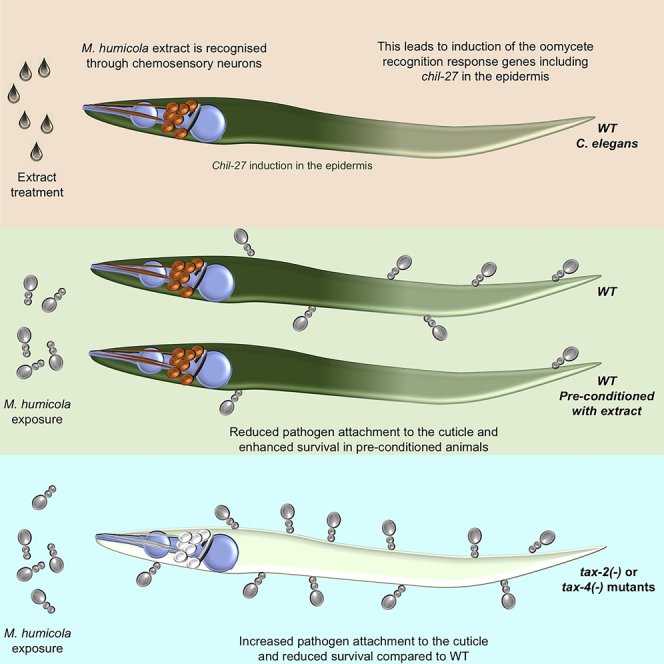

## Teaser

The oomycetes include key eukaryotic pathogens of animals and plants, but animal-oomycete interactions are little explored. Fasseas et al. report that *C. elegans* has evolved appropriate means to sense its natural oomycete pathogen *Myzocytiopsis humicola* and mount anticipatory defense response in the epidermis to protect from an upcoming infection threat.

## Introduction

Organisms can use multiple strategies to protect themselves from pathogens in their natural environment. These strategies can involve avoiding contact with the pathogen or resisting infection by mounting appropriate immune responses (Akira et al., 2006; Singh and Aballay, 2020). Innate immune responses can be both pathogen or host specific and can be generalized into two main types based on how they are initiated. Pathogen-associated molecular patterns (PAMPs) can act as signals for dedicated host receptors that then activate an intracellular cascade of events leading to the production of defense molecules (Akira et al., 2006). Alternatively, damage-associated molecular patterns (DAMPs) associated with perturbations of host physiology or tissue damage can also trigger the activation of innate immune signaling (Hou et al., 2019; Pujol et al., 2008). In both cases, pathogen detection, before and during infection, provides a strategy for organisms to strengthen the efficacy and specificity of their defense response (Mogensen, 2009).

The nematode *Caenorhabditis elegans* has been extensively used as a model for studying host-pathogen interactions for both naturally occurring (Schulenburg and Félix, 2017) and lab-induced (Sifri et al., 2003; Tan et al., 1999) infections. The former category is particularly useful to uncover host immune responses that have evolved to combat nematode infections in the wild, while the latter category may offer more direct links to biomedical challenges. Based on these infection models, various signaling pathways and immune effectors have been described to play a role in *C. elegans* defense (Ermolaeva and Schumacher, 2014; Ewbank and Pujol, 2016; Kim and Ewbank, 2018). Nevertheless, the characterization of the molecular pathways that underlie detection of various pathogens in *C. elegans* remains largely elusive (Meisel et al., 2014; Pradel et al., 2007; Pukkila-Worley et al., 2011; Twumasi-Boateng and Shapira, 2012).

We recently reported the identification of the oomycete *Myzocytiopsis humicola* as a natural pathogen of *C. elegans* (Osman et al., 2018). Oomycetes represent an evolutionarily distinct lineage of eukaryotes sharing morphological similarities with fungi but phylogenetic space with brown algae and diatoms in the Stramenopiles (Beakes et al., 2012). This group includes the crop pathogen *Phytophthora infestans*, infamous for the Irish famine in the mid-19^th^ century due to shortage of potatoes caused by the late blight disease (Fry, 2008). Although oomycetes have been mostly known as plant pathogens, they can also cause human infections, such as those caused by the oomycete *Pythium insidiosum* (De Cock et al., 1987; Krajaejun et al., 2006). The resulting animal disease, known as pythiosis or “swamp cancer,” is considered an emerging disease with significant mortality, especially in the tropics (Mendoza and Newton, 2005). The recent identification of oomycetes as natural pathogens of *C. elegans* offers an attractive model system to mechanistically study and dissect animal-oomycete interactions.

In contrast to bacterial pathogens that most often infect from the gut, *M. humicola* enters through the cuticle to rapidly kill *C. elegans* (Osman et al., 2018). In this study, we make use of this infection model to explore aspects of oomycete recognition in animals. We demonstrate that *C. elegans* can detect the oomycete, even in the absence of infection. We characterize in detail the transcriptional response of *C. elegans* to oomycete recognition and uncouple it from the response to tissue damage that occurs during infection. We provide evidence that *C. elegans* exposed to a non-infectious pathogen extract becomes less susceptible to oomycete infection through changes in the cuticle that reduce pathogen attachment. Interestingly, the response to extract and its protective effect is alleviated when chemosensation in sensory neurons is compromised. This work highlights the ability of *C. elegans* to sense its natural oomycete pathogen *M. humicola* and initiate anticipatory defense responses that reduce the chance of infection.

## Results

### *C. elegans* Can Sense Its Natural Oomycete Pathogen *M. humicola*

**Figure 1:**
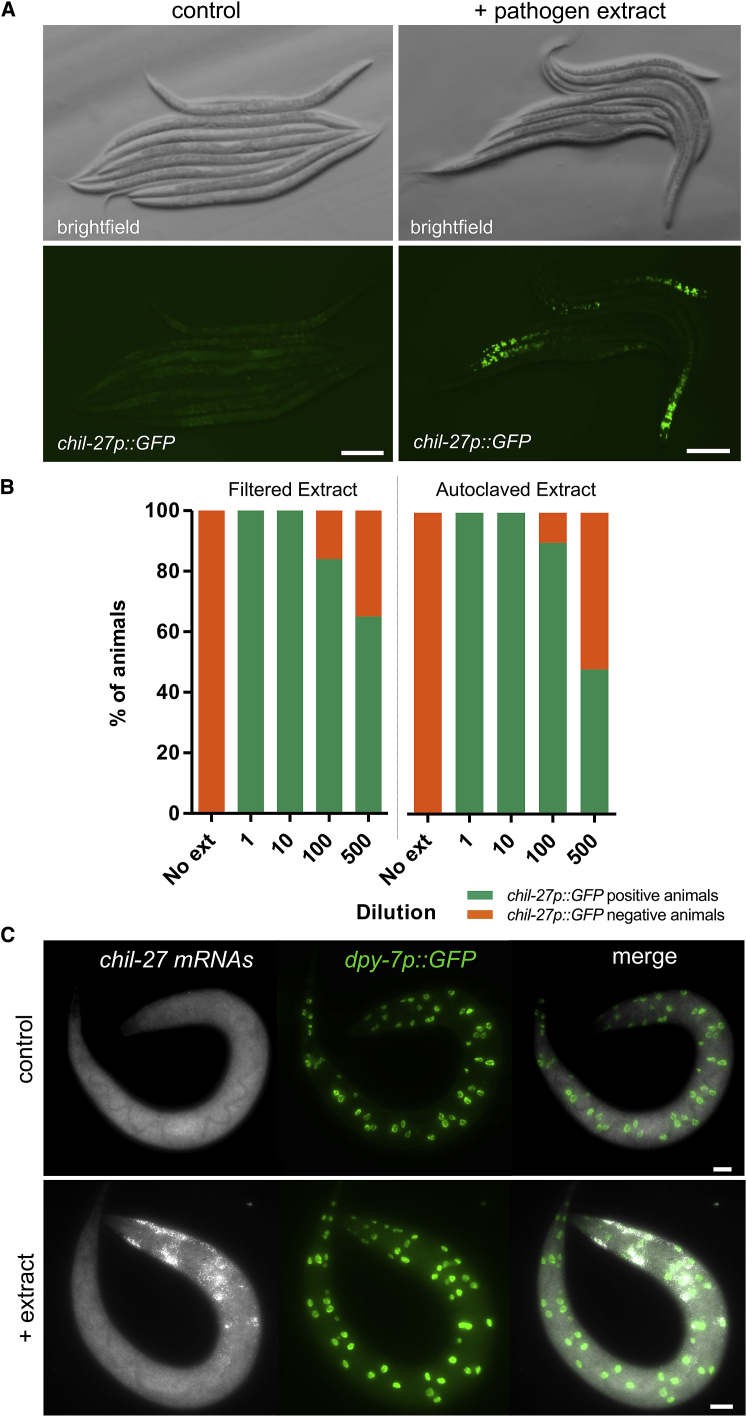
An Extract from *M. humicola*-Infected Nematodes Can Induce *chil-27p::GFP* Expression in *C. elegans* without Infection (A) A non-infectious extract made by washing infected plates with water leads to 100% *chil-27p::GFP* induction in the population. Control here refers to extract made from plates with no oomycete infection, and this treatment does not result in *chil-27p::GFP* expression (0% induction, n > 100). (B) Representative *chil-27p::GFP* induction assay upon dilution of filtered pathogen extract versus autoclaved extract. No significant difference is found between the two treatments. (C) Expression of *chil-27* by smFISH upon pathogen extract exposure. Image shows a second larval stage animal 1 h post extract or control treatment. A *dpy-7p::GFP* marker is used to visualize hypodermal nuclei. Note co-localization of *chil-27* mRNAs with hypodermal nuclei (hyp6 and anterior hyp7). Scale bars: 100 μm in (A) and 20 μm in (C). See also Figure S1.

We previously reported that *chitinase-like* (*chil*) gene induction is a hallmark of the *C. elegans* defense response to oomycete exposure and that this response is triggered upon exposure to both live and autoclaved *M. humicola* (Osman et al., 2018). This observation led us to hypothesize that *C. elegans* may be able to sense the oomycete pathogen to initiate defense responses that precede infection. To test this hypothesis, we decided to evaluate whether a pathogen extract derived from infected nematodes could also trigger a host defense response. We devised a method to produce an aqueous extract from infected populations of *C. elegans* that is non-infectious yet capable of inducing the expression of a transcriptional *chil-27p::GFP* marker (Figure 1A). This method consists of washing culture plates in which the infection has progressed with sterile water in parallel with plates that do not contain pathogen as a control. The derived pathogen extract was purified by centrifugation and filtration of the supernatant before application to nematode growth media. Efficacy was evaluated by performing serial dilution experiments and quantifying the dose-dependent induction of the *chil-27p::GFP* marker. Interestingly, we found that the pathogen extract is potent and can be diluted up to 500 times, while retaining more than 50% activity (Figure 1B). We also found that it is heat stable and maintains its activity after autoclaving (Figure 1B) as well as upon treatment with various degrading enzymes, such as β-glucanases, proteinases, and chitinases, among others (Figure S1A). Finally, the extract is stable over a long time and retains full activity with or without the *Escherichia coli* food source at 4°C for at least 2 months (Figure S1B). We observed in multiple attempts that animals expressing the *chil-27p::GFP* marker upon exposure to extract could not transmit the response when introduced to populations that had not been previously exposed. We conclude that the oomycete extract may contain a pathogen-derived molecule as its active component, which is both stable and abundant in our infected nematode cultures.

We next used the pathogen extract to explore the temporal dynamics of *chil-27p::GFP* induction. Using single molecule fluorescence *in situ* hybridization (smFISH), we could detect *chil-27* mRNAs induced in the epidermis (known as “hypodermis” in *C. elegans*) as early as 1 h after extract exposure (Figure 1C). Interestingly, *chil-27* transcripts localized first to the anterior side of the syncytial hypodermis, which is also reminiscent of the activation pattern of the *chil-27p::GFP* transcriptional marker (Figure 1A). Taken together, these results suggest that the pathogen extract and *chil-27p::GFP* induction represent a robust trigger and readout of pathogen detection, thereby offering a powerful system to dissect mechanisms of oomycete recognition using *C. elegans*.

### Exposure to Pathogen Extract Triggers an ORR in the Host

**Figure 2:**
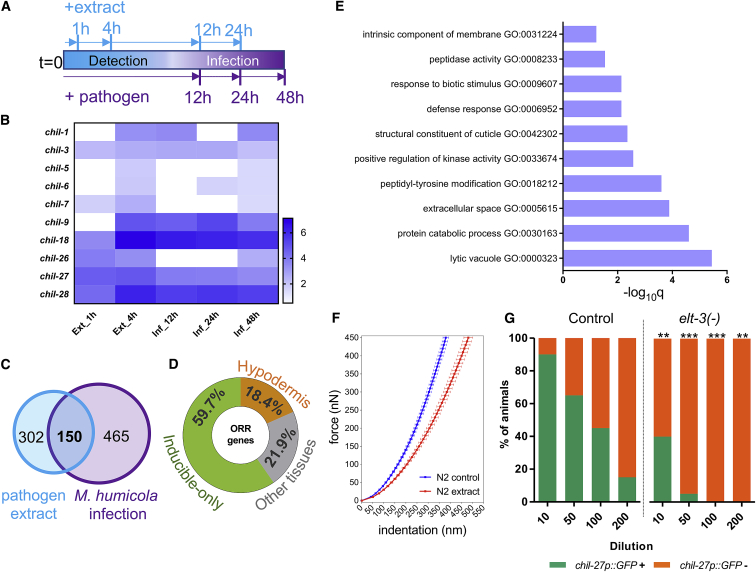
Transcriptional Response to Pathogen Extract Defines an Oomycete Recognition Response (A) Cartoon summarizing the design of the RNA-seq experiment. N2 animals at the L4 stage were exposed to pathogen extract for 1, 4, 12, and 24 h or to live pathogen for 12, 24, and 48 h, respectively. (B) Heatmap showing the members of the *chil* gene family that are differentially expressed by pathogen extract and infection. Heatmap color is based on Sleuth *b* values, which are analogous to fold change (Pimentel et al., 2017). White color indicates that the gene was not significantly upregulated in that condition (significance is defined as p value < 0.01 and FDR-adjusted p value < 0.1). (C) Venn comparison showing the overlap of differentially expressed genes by extract and *M. humicola* infection (pooled time points). This overlap is significant with a representation factor (RF) 8.3 and p value < 2.858 × 10^−99^ with a hypergeometric test. (D) Classification of upregulated ORR genes. A major fraction of these genes (n = 117) are only inducible upon extract treatment: 36 genes are reported to be expressed in the hypodermis, while 43 genes are not expressed in the hypodermis in wild type based on a threshold of <5 transcripts per million (Cao et al., 2017a). Pseudogenes have been removed, so 196 genes have been used in this analysis. (E) Gene Ontology (GO) enrichment analysis for the upregulated ORR genes using WormBase. Adjusted p value is shown on the x axis, and GO terms are listed on the y axis. (F) Force-displacement curves using atomic force microscopy. N2 animals treated with extract show reduction in stiffness, n > 20 animals per genotype. (G) Induction of *chil-27p::GFP* in *elt-3(gk121)* versus control (^∗∗∗^p value < 0.001 and ^∗∗^p value < 0.01 with a chi-square test for each independent dilution, n > 50 for each dilution). See also Figure S2 and Tables S1, S2, and S3.

We next sought to determine how the *C. elegans* transcriptional response to extract exposure may compare with infection with live pathogen. We reasoned that this experiment would allow us to uncouple responses underlying oomycete recognition from those attributed to tissue damage during infection. Our previous experiments indicated that *chil-27* is rapidly induced within the first hour of extract exposure, while it takes 24–48 h for the infection phenotype to appear, as manifested by the development of sporangia within the animal body (Osman et al., 2018). Therefore, we chose to include in the design of RNA sequencing (RNA-seq) experiments both early (1 and 4 h) and late (12, 24, and 48 h) time points post extract or pathogen treatment in parallel to control treatments with no exposure to extract or pathogen (Figure 2A). We found a strong transcriptional response to extract 1 and 4 h post exposure, which became much less detectable at 12 or 24 h (Table S1). For example, 268 genes were induced at 1 h and 250 genes at 4 h, with 78 genes being in common between the two time points. Among the induced genes, several members of the *chil* gene family were identified (Figure 2B). A total of 96 genes were found to be downregulated at 1 h and 109 genes at 4 h post extract treatment, but the overlap between time points was smaller, consisting of 16 genes in total (Table S1).

To define what may constitute an oomycete recognition response (ORR), we compared the infection and extract datasets by pooling all time points. Given that pathogen detection is part of the infection process, we anticipated that genes differentially regulated upon extract treatment would show a significant overlap with those identified upon exposure to live pathogen. Indeed, of 452 genes found to be upregulated in response to extract treatment by pooling all time points, 150 genes were also induced during infection with *M. humicola* (Figure 2C). We argued that genes detected exclusively upon extract treatment may still be part of a genuine pathogen recognition response. These genes may have been missed in the infection experiments due to undersampling of the time course of infection or because infection could mask the recognition process. Therefore, we decided to consider as strict ORR genes those that were common between extract treatment and infection as well as those uniquely found in the extract datasets if they showed a high magnitude of change (fold change ≥ 2 or ≤ −2) (Pimentel et al., 2017). The final ORR list consisted of 206 upregulated genes and 49 downregulated genes, a number of which were found to be conserved in humans (69/206 and 22/49 genes, respectively; Table S2) (Kim et al., 2018).

Most ORR genes were differentially expressed 4 h post extract exposure (183/206 induced and 34/49 downregulated genes) (Table S2). ORR genes were present on all chromosomes, but induced genes were overrepresented on chromosome V (Figure S2A). ORR genes were found to be primarily inducible genes (57% of the genes; Figure 2D) with minimal expression (a threshold of <5 transcripts per million) detected in wild-type tissues based on published single-cell RNA-seq (Table S2) (Cao et al., 2017a). An interesting feature of the ORR list is that it contained many genes expressed mostly outside the hypodermis (Figure 2D; Table S2), which may indicate systemic features in the response. However, this does not rule out that ORR genes get induced in the hypodermis upon oomycete exposure as part of a pathogen-specific recognition program.

We used Gene Ontology (GO) enrichment analysis (Angeles-Albores et al., 2016; Ge et al., 2019; Raudvere et al., 2019) to further characterize the obtained datasets upon extract treatment, as well as the derived ORR list (Figure 2E; Figures S2B and S2C; Table S3). The GO term chitin binding was found for all datasets by ShinyGo and g:Profiler, and this reflects the induction of several *chil* genes. We found enrichment of a cuticle composition term for the dataset obtained 1 h post extract treatment, which suggests a link between pathogen detection and the cuticle physical barrier. Consistent with this idea, atomic force microscopy (AFM) revealed that extract treatment changed the stiffness of the cuticle (Figure 2F). GO terms for defense response and response to a biotic stimulus were found 4 h post extract treatment (Figure 2E; Table S3). Other GO terms related to immunity, such as molecular degradation (e.g., protein catabolic processes), as well as cell signaling (e.g., transmembrane receptor kinase activity), were also found at 4 h (Figure 2E; Table S3) and may reflect pathways involved in the response to pathogen recognition. Finally, transcription factor binding analysis upstream of ORR genes using g:Profiler identified the hypodermally expressed GATA transcription factor ELT-3 as a putative regulator of the response (Table S3). We tested this prediction and found that *elt-3(gk121)* loss-of-function mutants are partially impaired in the induction of *chil-27p::GFP* in the hypodermis (Figure 2G).

**Figure 3:**
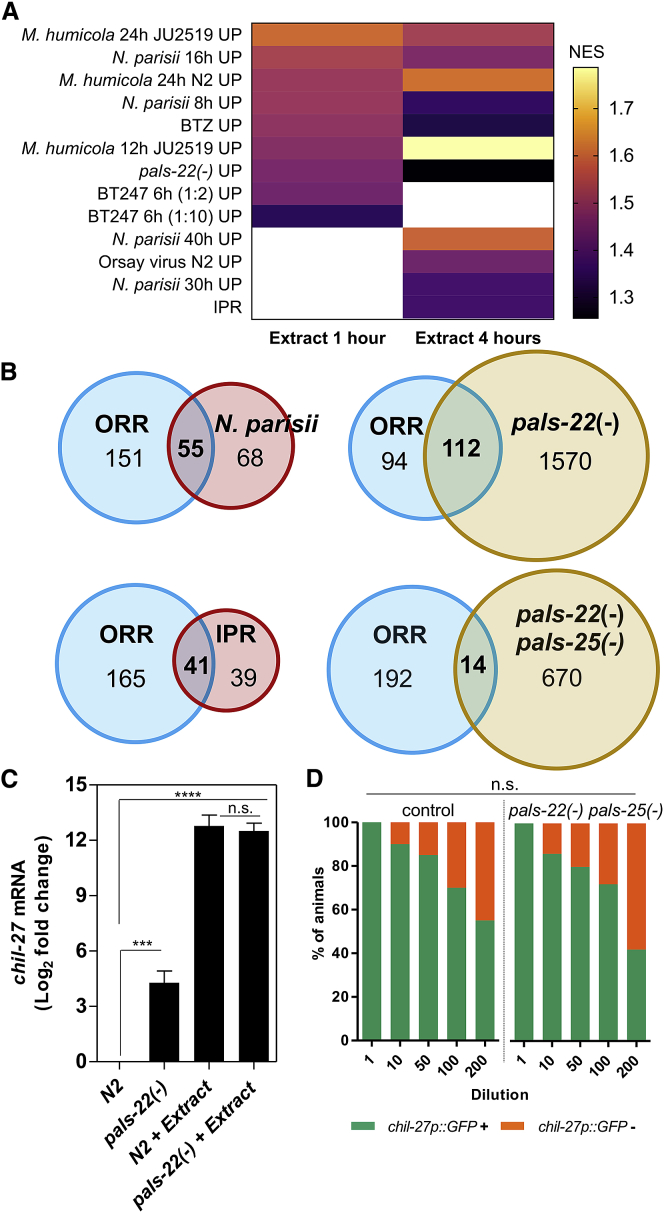
Comparative Analysis of the Oomycete Recognition Response (A) Heatmap presenting the normalized enrichment score (NES) derived from GSEA analysis and focusing on all gene sets showing significant intersection with our pathogen extract datasets. White color depicts no significant intersection (FDR < 0.25 and nominal p value < 0.05). (B) Venn diagrams comparing the upregulated ORR genes versus IPR (RF: 38.1, p < 1.892 × 10^−56^), *N. parisii* infection at 8 h (RF: 33.3, p < 9.031 × 10^−72^), and *pals-22(jy3)* (RF: 5, p < 1.070 × 10^−53^) and *pals-22(jy3) pals-25(jy9)* double mutants (RF: 1.5, non-significant). RNA-seq data used here are from Reddy et al. (2019). (C) qRT-PCR for *chil-27* expression in *pals-22*(*icb89*) treated with extract and N2 treated with extract (p < 0.001, one-way ANOVA and Tukey’s multiple comparison test) and N2 upon extract treatment. (D) *pals-22(icb90) pals-25(icb92)* double mutants respond to extract just like N2 at all extract dilutions (results are non-significant with a chi-square test for each independent dilution, n > 50 for each dilution). See also Figure S3 and Table S4.

To compare the response to oomycete extract exposure with the response against other known pathogens of *C. elegans*, we performed gene set enrichment analysis (GSEA) (Mootha et al., 2003; Subramanian et al., 2005). We found that datasets representing extract exposure for 1 and 4 h had significant intersections (nominal [NOM] p value < 0.05 and false discovery rate [FDR] < 0.25) with 9 and 11 of 65 datasets, respectively (Table S4). As expected, a significant intersection was found with datasets derived from infection by *M. humicola* (Figure 3A; Figure S3A; Table S4). The remaining significant intersections involved mostly datasets derived from time points post infection by the intestinal microsporidia *Nematocida parisii* (Figure 3A). It is of note that the intracellular pathogen response (IPR) (Reddy et al., 2017), which represents part of the response to microsporidia infection in *C. elegans*, was 51% shared with ORR (Figure 3B; Figure S3A). This is interesting given that these two independently derived gene lists concern evolutionary unrelated pathogens that use different infection strategies and exhibit distinct tissue tropism.

We previously reported an overlap in the machinery regulating the transcriptional response to microsporidia and oomycete infections (Reddy et al., 2019). This is because loss-of-function mutations in the protein containing ALS2CR12 signature (PALS) gene *pals-22* lead to constitutive *chil-27p::GFP* induction in the epidermis as well as induction of host responses against microsporidia in the intestine (Reddy et al., 2019). IPR and *chil-27p::GFP* induction rely on another member of the PALS family, PALS-25, as they are both suppressed in a *pals-22 pals-25* double-mutant background (Reddy et al., 2019). We therefore tested whether the ORR shares similarities with genes induced in a *pals-22* mutant background. Interestingly, 54% of induced ORR genes were also upregulated in *pals-22* mutants, while the overlap between ORR and *pals-22 pals-25* mutants was no longer significant (Figure 3B). These results indicate that PALS-25 is able to regulate a large proportion of ORR genes, when its suppression by PALS-22 is released (Reddy et al., 2019). Nevertheless, both *pals-22* and *pals-22 pals-25* mutants retained the ability to respond to extract to the same extent as control animals (Figures 3C and 3D). These results suggest that although the PALS-22/PALS-25 module can regulate ORR genes, it is unlikely to be directly involved in the response to extract perception.

The ORR gene list can serve as a signature of the host response pathway to *M. humicola* detection. It is however conceivable that the ORR list may also include genes that act, in turn, to enhance or suppress the recognition response. To test this idea, we performed a targeted RNAi screen to address whether knockdown of a subset of highly induced ORR genes would compromise *chil-27p::GFP* induction following extract treatment. We found no significant change in *chil-27p::GFP* induction in these RNAi experiments (Figure S3B). These results indicate that ORR genes are unlikely to be enriched for modulators or buffers of the response to oomycete detection.

### *C. elegans* Can Resist Infection by Detecting *M. humicola*

**Figure 4:**
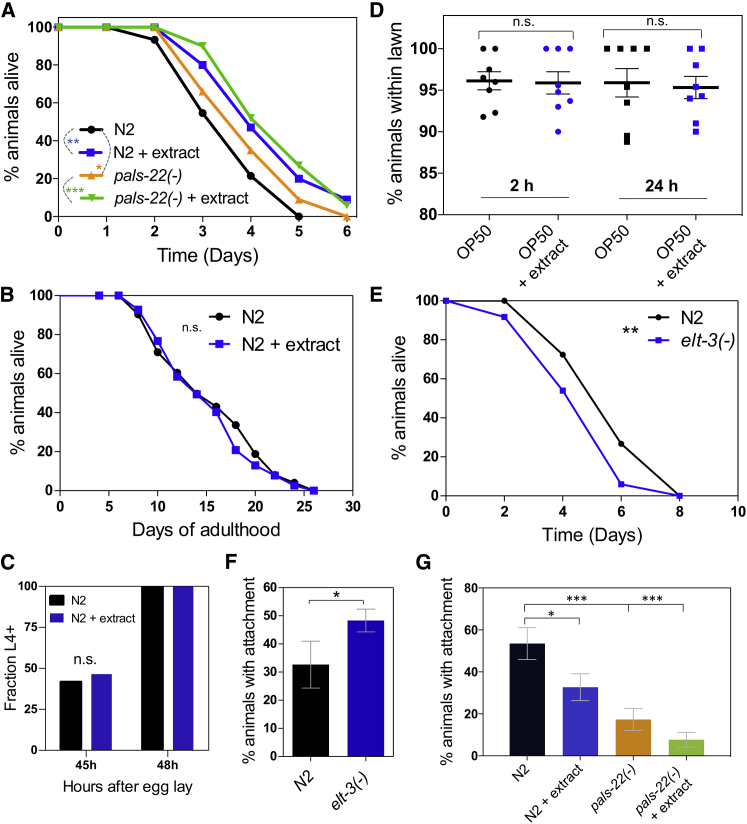
Exposure of *C. elegans* to Pathogen Extract Leads to Reduced Pathogen Attachment and Provides Protection from Oomycete Infection (A) Infection assay comparing N2 and *pals-22*(*icb89*) animals with or without previous exposure to extract (^∗∗∗^p value < 0.001, ^∗∗^p value < 0.01, ^∗^p value < 0.05 with a log rank test, n = 60–90 animals per condition). (B) Life-span comparison of animals treated with extract versus non-treated controls. No significant difference was found with a log rank test. (C) Quantification of developmental pace by measuring the fraction of animals at or beyond the L4 stage at two different time points. No difference was observed upon extract treatment with a chi-square test. (D) Avoidance assay depicting the percentage of animals leaving the lawn in 2 and 24 h with or without adding pathogen extract. (E) Survival curve of N2 and *elt-3(gk121)* animals at 20°C in the presence of *M. humicola* JUo1 (^∗∗^p < 0.01 with log rank test, n = 60 for each). (F) Percentage of N2 and *elt-3(−)* animals showing oomycete attachment after 4 h exposure with the pathogen at 20°C (^∗^p < 0.05 with chi-square test, n = 40). (G) Attachment assay for N2 and *pals-22(icb89)* animals with or without previous exposure to extract (^∗^p value < 0.05 and ^∗∗∗^p value < 0.001 with a chi-square test and comparisons made to N2 control, n = 50). See also Figure S4.

Pathogen recognition can provide a strategy for animals to prepare for the possibility of an upcoming infection. To test whether early detection of *M. humicola* by *C. elegans* can influence the outcome of infection, we compared the survival of animals exposed to extract before encountering the pathogen to controls that were encountering the pathogen without any prior extract treatment. We found that pretreatment with extract provided a mild, yet significant, protection from *M. humicola* infection compared with non-pretreated controls (Figure 4A). In a similar manner, *pals-22* loss-of-function mutants that show constitutive *chil-27p::GFP* expression, as well as induction of ∼50% of the ORR program, were also mildly more resistant to infection (Figure 4A). Consistent with the ability of *pals-22(icb89)* mutants to still respond to extract treatment, pretreatment with extract further enhanced their protection from infection (Figure 4A). It is of note that *pals-22* loss-of-function mutants display developmental delay, as well as shortened lifespan (Reddy et al., 2017), which may affect their performance in infection assays. However, we did not find an effect on lifespan or developmental speed upon extract treatment (Figures 4B and 4C). These results highlight that detection of *M. humicola* by *C. elegans* may be beneficial as a strategy for nematode survival.

Resistance to *C. elegans* pathogens may stem from mechanisms antagonizing pathogen entry and growth or behavioral changes leading to pathogen avoidance. However, we found that animals do not avoid an *E. coli* lawn supplemented with pathogen extract (Figure 4D). Currently, there is no evidence to suggest that *C. elegans* can clear filamentous eukaryotic pathogens when these have already penetrated the cuticle to initiate infection; thus, controlling pathogen entry into the nematode body is likely to be more crucial. We have previously proposed that induction of *chil* genes can modify the properties of the cuticle in a way that reduces pathogen attachment (Osman et al., 2018), so we focused here on whether changes in susceptibility correlate with oomycete attachment to the cuticle. As shown above, ELT-3 was predicted to regulate a part of ORR and its compromised function reduced *chil-27p::GFP* induction upon oomycete extract exposure. We found that *elt-3(gk121)* mutants were more sensitive to infection (Figure 4E), and this correlated with increased pathogen attachment (Figure 4F). Furthermore, we found a significant decrease in pathogen attachment in both *pals-22(icb89)* mutants and extract-treated animals (Figure 4G), which correlates with the increase in survival observed in infections assays. We conclude that detection of *M. humicola* may lead to anticipatory immunity through changes in the host cuticle that reduce pathogen attachment.

### Induction of *chil-27* in the Epidermis Is Modulated by the Chemosensory Neuron Proteins TAX-2/TAX-4

The main body hypodermis in *C. elegans* is a multinucleate syncytium, so we were surprised to find anterior localization of *chil-27* mRNAs within a single cell (Figure 1C). We argued that this localization may reflect signal transmission from the anterior side of the animal, which is dominated by neurons and glial cells that could readily play a role in pathogen detection. Furthermore, 13 of 43 ORR genes that were not expressed in hypodermis were exclusively expressed in neurons or neuronal support cells (Table S2). We therefore decided to test the possibility that neuronal signaling may regulate detection of *M. humicola* by *C. elegans*.

**Figure 5:**
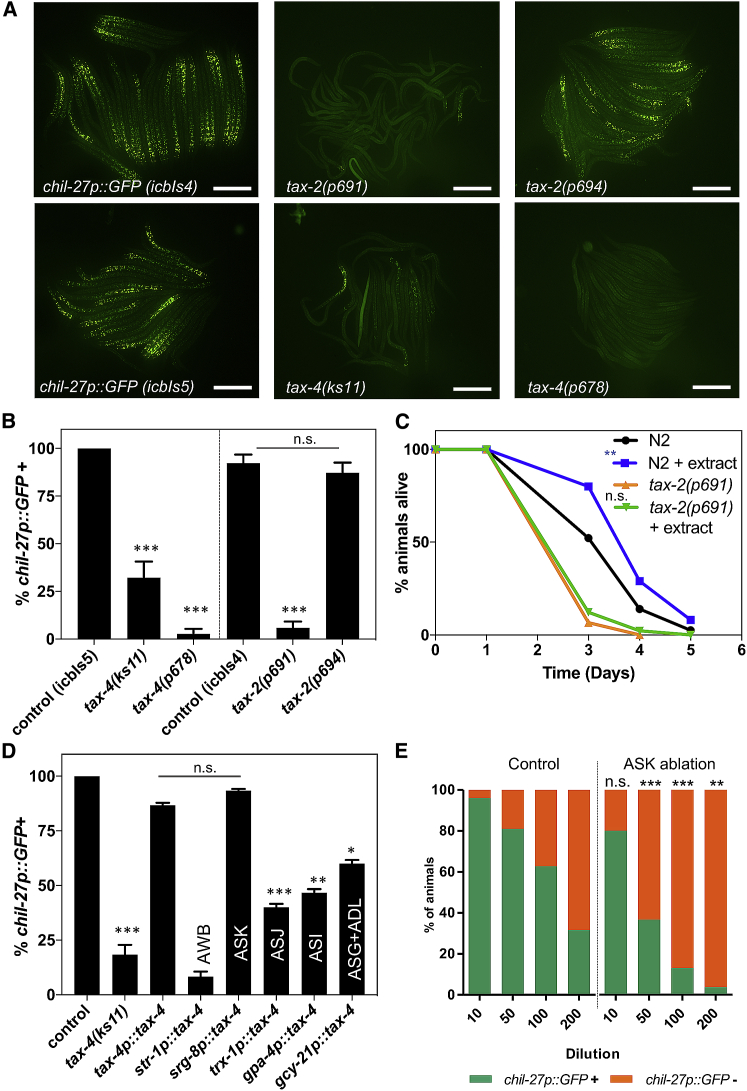
Neuronal Signaling Is Required for *C. elegans* Response to Pathogen Extract (A) *chil-27p::GFP* transgene induction assays in strains carrying *tax-2* or *tax-4* mutations using 1:100 dilution of the pathogen extract. Images show a group animals per genotype clustered together. Scale bars, 200 μm. (B) Quantification of induction assay for *tax-2* and *tax-4* mutants, n > 30. Note that strains carrying the *tax-2(p691)*, *tax-4(ks11)*, or *tax-4(p678)* alleles are strongly impaired in their ability to respond to extract (^∗∗∗^p value < 0.001 with a Fisher’s exact test in comparison with control). However, animals that carry the *tax-2(p694)* mutation that allows for *tax-2* expression in a subset of neurons respond normally. (C) Infection assay comparing N2 with *tax-2(p691)* mutants, with or without pre-exposure to extract (^∗∗^p value < 0.01 with a log rank test, n = 60–90 animals per condition). Note that *tax-2(p691)* is more susceptible than N2 to infection (^∗∗∗^p value < 0.001), but extract treatment does not have any protective effect. (D) Induction assays in strains carrying transgenes that rescue *tax-4* mutants in specific neurons. Note that rescue in ASK neurons leads to similar induction to that observed with the endogenous *tax-4* promoter, while no rescue or only partial rescue was observed in the case of other neuronal promoters (^∗∗∗^p value < 0.001, ^∗∗^p value < 0.01, ^∗^p value < 0.05 with a Fisher’s exact test, all comparisons are against the rescue observed using the endogenous *tax-4* promoter). Control induction in wild-type animals carrying *chil-27p::GFP* is shown as reference. (E) Quantification of *chil-27p::GFP* induction in ASK ablated *C. elegans* (through expression of a *sra-9p::mCasp-1* transgene) in response to serial dilution of the pathogen extract. ASK-ablated strain shows significantly reduced response to extract (^∗∗∗^p value < 0.001 and ^∗∗^p value < 0.01 with a chi-square test, n = 50 for each dilution). See also Figures S5 and S6.

We focused here on mutations in *tax-2* and *tax-4*, which encode cyclic nucleotide-gated channel α and β subunits responsible for cyclic guanosine monophosphate (cGMP)-activated cation channel activity in a subset of chemosensory neurons (Bretscher et al., 2011; Coburn and Bargmann, 1996). We found that loss-of-function alleles of *tax-2* (*p691*) and *tax-4* (*ks11*, *p678*) led to a strong reduction in the frequency of animals expressing *chil-27p::GFP* upon extract treatment (Figures 5A and 5B), highlighting a potential role for sensory neurons in the response. Failure to detect the pathogen should nullify the protective effect conferred by exposure to extract. We tested this idea by comparing wild-type N2 and *tax-2(p691)* animals in infection assays with and without pretreatment with extract. Although *tax-2(p691)* mutants were found to be more susceptible to infection than N2, they behaved in a similar manner with and without extract treatment (Figure 5C). This correlated with pathogen attachment assays in *tax-2(p691)* mutants, which showed increased attachment compared with N2, but no change upon extract exposure (Figure S4). These results implicate neuronal signaling in the underlying response to oomycete detection.

To narrow down the sensory neurons in which *tax-2/tax-4* are required for *M. humicola* detection, we made use of the *tax-2(p694)* allele, where *tax-2* expression is limited to six amphid neurons—AWB, AWC, ASG, ASI, ASK, and ASJ—due to a promoter deletion (Bretscher et al., 2011; Coburn and Bargmann, 1996). Interestingly, *tax-2(p694)* mutants carrying the *chil-27p::GFP* marker responded normally to extract exposure, indicating that expression within one or more of these neurons may be sufficient for the response (Figure 5D). To identify which neuron is involved, we pursued neuron-specific rescue experiments of the impaired *chil-27p::GFP* response to extract. Here, we used a *tax-4* rescue approach to take advantage of the *tax-4* locus that is smaller and thus more convenient for molecular cloning than *tax-2*. We drove *tax-4* expression in AWB, ASK, ASJ, ASI, and ASG/ADL using the *str-1*, *srg-8*, *trx-1*, *gpa-4*, and *gcy-21* promoters, respectively. We found that expressing *tax-4* under an ASK-specific promoter rescued the response of *tax-4* mutants to extract at a greater level than other neurons (Figure 5D). Consistent with this result, genetic ablation of the ASK neurons through expression of a *sra-9p::mCasp-1* transgene reduced the ability of the animals to induce *chil-27p::GFP* as a response to extract treatment (Figure 5E), while ablation of AWC or ASI did not have a similar effect (Figures S5A–S5D). To test whether the ASK neurons are likely to be the primary pathogen-sensing neurons, we monitored their activity upon extract exposure. However, we did not find a real-time calcium response in ASK neurons immediately following extract treatment (Figure S5E). We finally analyzed ASK-ablated animals for pathogen attachment and infection susceptibility, but we did not observe a statistically significant difference compared with wild-type animals (Figures S6F and S6G). Since ASK-ablated neurons do not exhibit complete loss of response to oomycete extract (Figure 5E), this suggests that other sensory neurons are likely to also be involved. Taken together, we conclude that ASK neurons modulate the pathogen-recognition immune response triggered in the epidermis of *C. elegans*.

## Discussion

We extend here our knowledge of host-pathogen interactions focusing on a diverse class of pathogens, the oomycetes, which naturally infect the model organism *C. elegans*. We present evidence that *C. elegans* is able to sense the oomycete *M. humicola* and mount a gene expression response to resist infection. Although *C. elegans* lacks key pattern recognition receptors that are present in other organisms, our work further suggests that the nematode can distinguish between the pathogens it encounters (Irazoqui et al., 2010; Wong et al., 2007). Since *M. humicola* is an obligate pathogen of *C. elegans* and cannot be grown so far in pure culture, we cannot formally rule out the possibility that the active component of the extract is produced by infected animals. However, several observations suggest that the signal is more likely to initiate directly from the pathogen. We have previously shown that mechanical damage of the cuticle by microinjection is unable to induce the *chil-27p::GFP* response (Osman et al., 2018), which is in contrast to the induction of protective antimicrobial peptides in response to cuticular damage upon infection by other epidermal pathogens, such as the fungus *Drechmeria coniospora* (Pujol et al., 2008; Zugasti et al., 2014). Extracts prepared from plates containing animals infected with *D. coniospora* were shown to be unable to induce *chil-27p::GFP* expression and so were extracts prepared from *pals-22(icb89)* mutants that exhibit constitutive *chil* gene expression (Figure S1C). *C. elegans* is known to produce ascarosides under stress, which are sensed through various chemosensory neurons, including the ASK neurons (Kim et al., 2009; Ludewig et al., 2013). However, extracts made from infected *daf-22(m130)* animals, which are defective in ascaroside biosynthesis (Butcher et al., 2009), induce the immune response at a comparable level to extracts made from infected N2 animals (Figure S1C). Taken together, these observations are less compatible with the idea of a host-derived transmissible danger signal that propagates the response in the nematode population. Instead, we suggest that the active component in the extract is more likely to be a PAMP that is sensed by *C. elegans.* Such oomycete-derived molecules may correspond to essential factors of the pathogen’s life cycle or infection strategy, for example, cell wall components or secreted virulence factors, as previously described for plant hosts (Fawke et al., 2015).

We uncouple here the response to pathogen recognition (ORR) from responses to tissue damage that occur during infection. The hallmark of the response to *M. humicola* infection is the induction of the *chil* gene family (Osman et al., 2018), which we report here also occurs upon exposure to pathogen extract. The function of *chil* genes has been linked to collagen synthesis and degradation in some systems (Bigg et al., 2006; Iwata et al., 2009), and induction of *chil* genes in *C. elegans* has been proposed to modify properties of the cuticle in a way that reduces pathogen attachment (Osman et al., 2018). Consistent with this model, AFM revealed changes in stiffness upon extract treatment, and extract-treated animals showed reduced pathogen attachment. Although these observations strongly suggest cuticle changes upon oomycete recognition, whether these changes remain purely at a biochemical level or whether they also have morphological consequences remains unknown. Besides cuticle modification, the induced ORR genes may play a role in other responses conferring a protective effect against oomycete infection. For example, changes in host metabolism are likely to influence immunity, and it is interesting that the ORR list includes a number of metabolic genes. It is conceivable that behavioral changes would represent a putative strategy for nematodes to reduce infection in the wild; however, we did not see such an effect in lab conditions. We hypothesize that ORR genes are more likely to represent effectors of the immune response, as opposed to the machinery required for oomycete detection, which is emphasized by the fact that many ORR genes are only expressed upon exposure to pathogen extract.

Responses of *C. elegans* to different pathogens are thought to be largely distinct. It is therefore interesting that the ORR gene list significantly overlaps with datasets derived from microsporidial or viral infections, which are intracellular pathogens of the intestine (Bakowski et al., 2014). We report here that *pals-22* loss-of-function mutants show induction of more than 50% of the ORR genes, which correlates with their increased resistance to oomycete infection through a reduction in pathogen attachment. It is of note that all shared genes between ORR and IPR are also common between ORR and *pals-22* mutants. Therefore, we argue that the overlap between microsporidial infections and *M. humicola* detection relates to the *pals-22/25* regulatory circuit. Interestingly, *pals-22* and *pals-25* mRNA levels do not change upon extract treatment and infection, and *chil* gene induction upon extract treatment is not compromised in *pals-22* and *pals-22 pals-25* double mutants. This indicates that oomycete detection is unlikely to act through direct changes in the PALS-22/PALS-25 module, although PALS-22/PALS-25 and the extract response pathway may converge on the regulation of a yet unknown downstream factor that triggers *chil* gene expression and cuticle remodeling.

Epidermal immune responses are thought to counteract pathogens that infect through the cuticle. We report here that induction of *chil-27* in the epidermis is partially dependent on the GATA transcription factor ELT-3. This family of transcription factors includes key regulators of pathogen-induced genes and dominates tissue-specific immune responses in *C. elegans* (Block and Shapira, 2015; Shapira et al., 2006; (Yang et al., 2016a)). However, our results support a model wherein the immune response triggered in the epidermis relies on oomycete recognition in neurons. Neuron-to-epidermis communication has been shown to occur during fungal infections and leads to antimicrobial peptide induction in the epidermis (Zugasti and Ewbank, 2009). Our model of neuron-to-epidermis communication culminating in cuticular changes is reminiscent of some recently reported neural regulation of the cuticle barrier, through the neuropeptide Y receptor NPR-8, in response to infection by bacterial pathogens (Sellegounder et al., 2019). NPR-8 is not directly involved in sensing bacteria and acts to suppress cuticular collagen genes that contribute to host defense (Sellegounder et al., 2019). Interestingly, neuron-to-epidermis regulation in the oomycete context is linked to pathogen recognition and does not appear to involve regulation of the same collagen genes, which are not part of the identified ORR. Therefore, neuronal regulation of cuticle remodeling may provide a broad mechanism for protection against pathogens infecting through the epidermis and also those that colonize the *C. elegans* intestine.

Our results are consistent with reports of various sensory neurons modulating innate immune pathways, physical barrier defense, and behavior in *C. elegans* (Cao et al., 2017b; Foster et al., 2020; Styer et al., 2008). TAX-2/4-dependent signaling in chemosensory neurons has been previously linked to cuticle morphology and epidermal pathogen avoidance (Bretscher et al., 2011; Coburn and Bargmann, 1996; Sellegounder et al., 2019; Yook and Hodgkin, 2007). Tissue-specific rescue and neuronal ablation experiments revealed ASK neurons to be the most prominent in modulating the response to oomycete recognition. While ASK neurons have been previously associated with avoidance of certain chemical repellents (Hilliard et al., 2002), an involvement in modulation of pathogen detection has not been reported. Given that ASK-ablated animals are still able to respond to pathogen extract, and restoration of *tax-4* function in other sensory neurons does partially rescue *chil-27p::GFP* induction in *tax-4(ks11)* mutant animals upon extract treatment, we anticipate that other neurons also contribute to this response. The lack of a detectable calcium response in ASK further suggests that these may not be the primary oomycete-sensing neurons, although they are involved in modulating the mounting of the immune response in the hypodermis. A similar scenario has been reported in another study where AWB, AWC, and ASJ neurons regulate cuticle dynamics in response to bacterial infection in *C. elegans*, but none of these neurons are actually involved in sensing the pathogenic bacteria (Sellegounder et al., 2019). Future work will focus on dissecting the machinery and specificity of neuronal and intercellular signaling in the context of oomycete recognition. We speculate that such cross-tissue communication may be an important feature of animal defense to oomycete infection.

## STAR★Methods

### Key Resources Table

**Table:** 

REAGENT or RESOURCE	SOURCE	IDENTIFIER
**Bacterial and Virus Strains**		

*E. coli*: OP50	Caenorhabditis Genetics Center	Wormbase ID: OP50
Ahringer RNAi Libraries in *E. coli* HT115	Source Bioscience	N/A

**Chemicals, Peptides, and Recombinant Proteins**		

Calcofluor White	Sigma	Cat: 910090
IPTG	Sigma	Cat: I6758
DNase I	Promega	Cat: Z358A-C
TRIzol	Invitrogen	Cat: 15596026
SuperScript IV Reverse Transcriptase	Invitrogen	Cat: 18090010

**Critical Commercial Assays**		

LightCycler480 SYBR Green I Master	Roche	Cat: 4707516001

**Deposited Data**		

RNA-seq data, normalized per gene sequence counts and differentially expressed genes as determined by Kallisto & Sleuth respectively	This paper	GEO: GSE150135

**Experimental Models: Organisms/Strains**		

*M. humicola* Lisbon isolate	Osman et al., 2018	JUo1
*C. elegans: wild-type N2*	Caenorhabditis Genetics Center	Wormbase ID: N2
*C. elegans: unc-119(ed3) III; icbSi3[dpy-7::GFP::H2B::unc-54 3′UTR+cb-unc-119]*	This paper	MBA260
*C. elegans: icbIs4[chil-27p::GFP, col-12p::mCherry-pest] II*	Osman et al., 2018	MBA281
*C. elegans: icbIs5[chil-27p::GFP, col-12p::mCherry-pest] IV*	Osman et al., 2018	MBA282
*C. elegans: pals-22(icb89)III*	This paper	MBA1080
*C. elegans: elt-3(gk121); icbIs4[chil-27p::GFP, col-12p::mCherry-pest] II*	This paper	MBA397
*C. elegans: pals-22(icb90) pals-25(icb92) III; icbIs4[chil-27p::GFP, col-12p::mCherry-pest] II*	Reddy et al., 2019	MBA791
*C. elegans: tax-2(p691) I; icbIs4[chil-27p::GFP, col-12p::mCherry-pest] II*	This paper	MBA507
*C. elegans: tax-2(p694) I; icbIs4[chil-27p::GFP, col-12p::mCherry-pest] II*	This paper	MBA667
*C. elegans: tax-4(ks11) III; icbIs5[chil-27p::GFP, col-12p::mCherry-pest] IV*	This paper	MBA668
*C. elegans: tax-2(p691) I*	Caenorhabditis Genetics Center	Wormbase ID: PR691
*C. elegans: icbIs5[chil-27p::GFP, col-12p::mCherry-pest] IV; tax-4(ks11) III.; icbEx246[pFD18(tax-4::tax-4::wrmScarlet::SL2::unc-54),pRJM163 (bus-1::GFP), BJ36]*	This paper	MBA1069
*C. elegans: icbIs5[chil-27p::GFP, col-12p::mCherry-pest] IV; tax-4(ks11) III.; icbEx247[pFD19(str-1::tax-4::wrmScarlet::SL2::unc-54),pRJM163 (bus-1::GFP), BJ36]*	This paper	MBA1070
*C. elegans: icbIs5[chil-27p::GFP, col-12p::mCherry-pest] IV; tax-4(ks11) III.; icbEx243[pFD8(srg-8::tax-4::wrmScarlet::SL2::unc-54),pRJM163 (bus-1::GFP), BJ36]*	This paper	MBA1066
*C. elegans: icbIs5[chil-27p::GFP, col-12p::mCherry-pest] IV; tax-4(ks11) III.; icbEx242[pFD7(trx-1::tax-4::wrmScarlet::SL2::unc-54),pRJM163 (bus-1::GFP), BJ36]*	This paper	MBA1065
*C. elegans: icbIs5[chil-27p::GFP, col-12p::mCherry-pest] IV; tax-4(ks11) III.; icbEx244[pFD9(gpa-4::tax-4::wrmScarlet::SL2::unc-54),pRJM163 (bus-1::GFP), BJ36]*	This paper	MBA1067
*C. elegans: icbIs5[chil-27p::GFP, col-12p::mCherry-pest] IV; tax-4(ks11) III.; icbEx245[pFD11(gcy-21::tax-4::wrmScarlet::SL2::unc-54),pRJM163 (bus-1::GFP), BJ36]*	This paper	MBA1068
*C. elegans: icbIs4[pGO4, pCMH1195] II; qrIs2[sra-9p::mCasp-1, elt-2p::GFP]*	This paper	MBA1081
*C. elegans: oyIs85 [ceh-36p::TU#813 + ceh-36p::TU#814 + srtx-1p::GFP + unc-122p::DsRed];icbIs5[chil-27p::GFP, col-12p::mCherry-pest] IV.*	This paper	MBA665
*C. elegans: oyIs84 [gpa-4p::TU#813 + gcy-27p::TU#814 + gcy-27p::GFP + unc-122p::DsRed]; icbIs5[chil-27p::GFP, col-12p::mCherry-pest] IV.*	This paper	MBA666
*C. elegans: daf-22(m130) II*	Caenorhabditis Genetics Center	Wormbase ID:DR476
*C. elegans: elt-3(gk121) X*	Caenorhabditis Genetics Center	Wormbase ID:VC143
*C. elegans: qrIs2[sra-9p::mCasp-1, elt-2p::GFP]*	Caenorhabditis Genetics Center	Wormbase ID:PS6025
*C. elegans: ljEx1186[sra-9p::GCaMP3::SL2-tagRFP(pWRS1483); unc-122p::RFP]*	This paper	AQ4259

**Oligonucleotides**		

oligos, see Table S6	Eurofins Genomics	N/A
smFISH probe, see Table S6	Biomers.net	N/A

**Recombinant DNA**		

*trx-1p::tax-4:::wrmScarlet::SL2::unc-54*	This paper	pFD7
*srg-8p::tax-4::wrmScarlet::SL2::unc-54*	This paper	pFD8
*gpa-4p::tax-4::wrmScarlet::SL2::unc-54*	This paper	pFD9
*gcy-21p::tax-4::wrmScarlet::SL2::unc-54*	This paper	pFD11
*str-1p::tax-4::wrmScarlet::SL2::unc-54*	This paper	pFD19
*tax-4p::tax-4::wrmScarlet::SL2::unc-54*	This paper	pFD18

**Software and Algorithms**		

GraphPad Prism 7	GraphPad Software, La Jolla, CA	https://www.graphpad.com/scientific-software/prism/
ImageJ	NIH Image	https://imagej.nih.gov/ij/
LightCycler480 (version 1.5.1.62)	Roche	N/A
Kallisto	Bray et al., 2016	https://pachterlab.github.io/kallisto/
Gene Set Enrichment Analysis (GSEA) software v4.0.3	Mootha et al., 2003	https://www.gsea-msigdb.org/gsea/index.jsp
	Subramanian et al., 2005	

### Resource Availability

#### Lead Contact

Further information and requests for resources and reagents should be directed to and will be fulfilled by the Lead Contact, Michalis Barkoulas (m.barkoulas@imperial.ac.uk).

#### Materials Availability

All unique/stable reagents generated in this study are available from the Lead Contact without restriction.

#### Data and Code Availability

The raw RNA-seq data have been deposited to NCBI GEO under accession GEO: GSE150135.

### Experimental Model and Subject Details

*C. elegans* strains were cultured on NGM plates seeded with *E. coli* OP50 at 20°C under standard conditions (Stiernagle, 2006). *M. humicola* was grown and maintained at 25°C on NGM plates along with *C. elegans* N2. The oomycete cultures can be maintained indefinitely by chunking onto fresh NGM plates with OP50. For use in experiments, freshly killed animals that are full of sporangia were washed off plates that were chunked 2-3 days earlier. Infected animals were separated from live animals using 70 μm nylon filter mesh immersed in M9 buffer. This allowed for live animals to swim through the mesh while the dead animals were retained. The dead animals were gently disrupted using a grinder in a microcentrifuge tube and the numbers of sporangia were counted under a microscope. Dilutions of these suspensions were made using M9 and they were added to standard NGM plates with OP50 to be used in infection or attachment assays. A list of strains used in this study is presented in the Key Resources Table.

### Method Details

#### Pathogen extract preparation

Crude pathogen extract was prepared by growing *M. humicola* strain JUo1 on 90mm standard or egg yolk plates with N2 until starvation. Plates were washed with 5 mL H_2_O each and the suspension was centrifuged in 50 mL tubes for 20 minutes at 5000 rpm to remove debris. The supernatant was collected, filter-sterilized using 0.2 μm filters and autoclaved. The extract was stored for long term at −80°C until use. The control extract was also prepared in the same way from N2 animals grown in the absence of any pathogen.

#### Phenotypic assays

All infection, attachment and lifespan assays were performed at 20°C in triplicates on standard NGM plates with OP50. For populations treated with extract, this was added at the L2 stage. Infection assays were started by moving 30 L4 animals to plates containing *M. humicola* (as described above) and experiments were done in triplicates, so 90 animals were used per condition in total. The animals were scored for visible infection (i.e., development of sporangia) and the live ones were moved to new plates containing *M. humicola* for a period up to 7 days, depending on how the infection progressed. Dead animals without signs of infection or lost animals were censored. N2 animals were bleached 8h after *pals-22(-)* mutants in order to have synchronized development as the latter exhibit developmental delay. For lifespan assays, the same conditions were used without the presence of oomycete and the assay continued until all animals had died, while lost or bagged animals were censored. All experiments were repeated at least three times (Table S5) and GraphPad Prism (GraphPad Software Inc.) was used to plot and compare survival curves. The log-rank test was used to assess statistical significance and a *p* value of < 0.05 was considered significant.

For the attachment assays, day 1 adult animals were moved to plates containing *M. humicola* at approximately 100 animals per plate. They were left for 4h at 20°C and then washed with M9 buffer. Attachment was scored by two methods, using FISH as previously described (Osman et al., 2018) and imaged using an epifluorescence Ti-eclipse (Nikon) microscope equipped with a low noise CCD camera (Andor iKon-M934), or by staining briefly in calcofluor white (Sigma) and visualized with a DAPI filter. Images were analyzed using ImageJ. A chi-square test was used to test significance of the results.

For induction assays, serial dilutions of the extract were made in water and 100 μL of each was added to OP50 plates. The extract was added on top of the bacterial lawn and the plate was moved gently to allow it to spread uniformly. *C. elegans* eggs suspended in M9 were added to each plate to have 100-150 eggs in total. The plates were incubated at 20°C and scored for *chil-27p::GFP* induction after 48h using Zeiss Axio Zoom V16 (Zeiss) microscope. Animals were classified in two groups (GFP positive and negative) and *col-12p::mCherry* expression was confirmed in both cases. For RNAi experiment, 6-8 L4 stage worms were added onto respective RNAi plates containing extract and *chil-27p::GFP* induction was scored after 72h when the progeny on the plate were predominantly L4 stage or beyond. All RNAi clones were obtained from the Ahringer collection (Source BioScience) and were verified by sequencing. The expression of dsRNA was induced by the addition of 1 mM isopropyl β-D-1-thiogalactopyranoside (IPTG; Sigma).

For the avoidance assay, 100 L4 stage animals suspended in M9 were spotted in the center of an OP50 plate with and without extract; and animals present within the lawn were counted after 2h and 24h.

#### qRT-PCR

*C. elegans* at L4 stage were treated with extract for 4 h and total RNA was extracted from animals grown at the appropriate stage/exposure time using the TRIzol reagent (Invitrogen) and isopropanol/ethanol precipitation. NanoDrop quantification (Thermo Scientific) and gel electrophoresis was used to assess the quantity and quality of RNA. 2 μg of RNA from all samples was subjected to DNase (Promega) treatment and cDNA was synthesized using Superscript IV (Invitrogen) with Oligo(dT) primers as per manufacturer’s instructions. Real-time PCR was performed using LightCycler480 SYBR Green I Master (Roche) in a LightCycler480 instrument and Ct values were derived using the LightCycler480 software and second derivative maximum method. Expression levels of *chil-27* were normalized with the values obtained for the reference gene *pmp-3.* The efficiency of each set of primers and calculation of levels of induction was determined as described previously (Pfaffl, 2001). Experiments were performed in biological triplicates and oligos used are listed in the Key Resources Table.

#### RNA-seq

*C. elegans* populations were synchronized by bleaching and grown until the L4 stage. For all extract treatments, 300 μL of undiluted extract was added to NGM plates. Infection samples were exposed to conditions as described for the infection assays. RNA was extracted in triplicates at 1h, 4h, 12h and 24h for extract samples and 12h, 24h and 48h post exposure to the pathogen. RNA samples were sequenced by BGI Genomics (Hong Kong). Pseudoalignment was performed using Kallisto (Bray et al., 2016) and the WS235 transcriptome from Wormbase. Analysis of counts was performed using Sleuth and a Wald test for two-sample comparisons (Pimentel et al., 2017).

#### GSEA

The RNA-seq datasets from exposure to extract at 1h and 4h were compared to other *C. elegans* transcriptomic datasets using Gene Set Enrichment Analysis (GSEA) software v4.0.3 (Mootha et al., 2003; Subramanian et al., 2005). The genes that were significantly differentially expressed upon infection (FDR < 0.1) were ranked based on their b value in descending order. We used 65 gene sets for the analysis derived from (Osman et al., 2018), (Reddy et al., 2019) and WormExp ((Yang et al., 2016b), which can be found in Table S3). Pre-ranked analysis with weighted enrichment statistic, 1000 permutations and a minimum of 15 genes overlap, was performed independently for extract treatment at 1h and 4h time points. The NES-values of gene sets with FDR < 0.25 and nominal p value < 0.05 were considered as significant and the results are summarized in Table S3.

#### Molecular cloning and transgenesis

To generate constructs for neuron-specific rescue of *tax-4* mutants, splice leader 2 (SL2) sequence was amplified from genomic DNA using primers SL2-F and SL2-R. Restriction sites and Gibson Assembly complementarity arms were added to this SL2 amplicon using primers SL2-F Gib and SL2-R. WrmScarlet followed up by *unc-54* 3′UTR was amplified from the plasmid pDK38C2 using primers SL2 scarlet-F and BJ36-unc54-ter-R. Fragments were cloned using a Gibson Assembly protocol into SpeI-digested BJ97 to give the intermediate vector pFD1. Promoter regions and *tax-4* coding sequence were amplified from genomic DNA and cloned into AvrII-digested pFD1 using Gibson Assembly. Constructs were injected into the *tax-4(ks11)* strain at a concentration of 5ng/μl along with 20ng/μl of *bus-1p::GFP* (pRJM163) as a co-injection marker. A list of oligos used in this study is presented in the Key Resources Table.

#### Microscopy

Single molecule FISH was performed as previously described (Hintze et al., 2020; Katsanos et al., 2017). Briefly, animals were synchronized by bleaching and treated with extract for 1 hour at mid-L2 stage. Animals were fixed with 4% formaldehyde (Sigma-Aldrich) in 1x PBS (Ambion) for 45 min and were permeabilised with 70% Ethanol for 24 hours. Hybridization was performed at 30°C for 16 hours. A list of oligos included in the *chil-27* probe can be found in the Key Resources Table. Imaging was performed in an inverted and fully motorised epifluorescence microscope (Nikon Ti-eclipse) with an iKon M DU-934 CCD camera (Andor) controlled via the NIS-Elements software (Nikon) using the 100x objective. Atomic force microscopy was performed as previously described (Osman et al., 2018) using day 1 N2 adults grown on plates supplemented with oomycete or N2 control extract. Calcium imaging was performed on day 2 adult animals in a custom-designed microfluidic devices as previously described (Chew et al., 2018; Cho et al., 2018; Cho et al., 2017). Briefly, experiments were performed on a Leica Axiovert 135 inverted microscope using a 40x air objective. Video sequences were captured using a Hamamatsu ORCA-R2 (C10600-10B) camera with 100 msec exposure time. Simultaneous dual color imaging was performed using an OptoSplit II (Andor Technology) beamsplitter containing a GFP (520 nm)/RFP(605 nm) filter set. CoolLED’s pE-300 white was used as a light source for fluorescent imaging. Imaging was carried out in S-basal buffer (100mM NaCl, 0.05M phosphate buffer pH 6.0, 5 μg/mL cholesterol). Stimulus was 1/10 diluted pathogen extract in S-basal as a 10 s pulse and was delivered at t = 5 s after recordings were started. Videos were recorded for 25 s following stimulus delivery. For analysis of calcium transients, fluorescence intensities for each frame were extracted using a custom MATLAB script (Cho et al., 2018; Cho et al., 2017).

### Quantification and Statistical Analysis

Graphic representation and statistical analysis were performed using the GraphPad Prism 7 software. Data shown in bar graphs indicate mean, and error bars represent standard error of the mean or standard error of the proportion as indicated. A log-rank, chi-square, Fisher’s exact test, ANOVA and Tukey’s multiple comparison test was used to analyze data as described in the figure legends. Results were considered statistically significant when p < 0.05. Asterisks in figures indicate corresponding statistical significance as it follows: ^∗^ p < 0.05; ^∗∗^ p < 0.01; ^∗∗∗^ p < 0.001; ^∗∗∗∗^ p < 0.0001. Overlaps between gene lists were assessed based on a hypergeometric test (nemates.org).

## Supplemental Information

Document S1. Figures S1–S5

Table S1. RNA-Seq Results of Animals Treated with Pathogen Extract and Live Pathogen, Related to Figure 2 Extract treatments (ext) and infections (inf) were performed using *M. humicola* isolate JUo1. Sleuth *b* values are analogous to fold change. Tables also show transcript names, Wormbase Gene IDs, gene/sequence names are shown, Wald test *p* value and p value adjusted for multiple test correction.

Table S2. Defining an Oomycete Recognition Response (ORR), Related to Figure 2 (A) Upregulated genes, (B) Downregulated genes, (C) Criteria used in ORR definition, (D) Overlap between ORR genes and other datasets, (E) ORR genes with human homologs, (F) Predicted tissue distribution of induced ORR genes.

Table S3. GO Term Analysis of Genes Induced upon Pathogen Extract Exposure, Related to Figure 2 Induced genes 1 hour (A) and 4 hours (B) post extract treatment, and (C) induced ORR genes. Results from ShinyGO, g:Profiler and Wormbase are shown.

Table S4. GSEA Analysis of Genes Identified upon Pathogen Extract Exposure, Related to Figure 3 (A) GSEA results, (B-C) Gene overlap for all significant intersection using induced genes 1 hour (B) and 4 hours (C) post extract treatment, (D) Percentage of gene overlap between the two extract datasets and all other datasets used in GSEA analysis, (E) Gene sets used in GSEA analysis.

Table S5. Survival Data for Infection and Lifespan Assays, Related to STAR Methods

Table S6. Oligos Used in This Study, Related to STAR Methods

Document S2. Article plus Supplemental Information
