# Supplementary material for: Chemosensory Neurons Modulate the Response to Oomycete Recognition in Caenorhabditis elegans
Source: Cell Rep. 2021 Jan 12;34(2):108604. doi: 10.1016/j.celrep.2020.108604 (PMC7809619; doi:10.1016/j.celrep.2020.108604)
Supplement: Document S1. Figures S1–S5 [file mmc1.pdf]

Cell Reports, Volume *34*

Supplemental Information

Chemosensory Neurons Modulate the Response to Oomycete Recognition in *Caenorhabditis elegans*

Michael K. Fasseas, Manish Grover, Florence Drury, Clara L. Essmann, Eva Kaulich, William R. Schafer, and Michalis Barkoulas

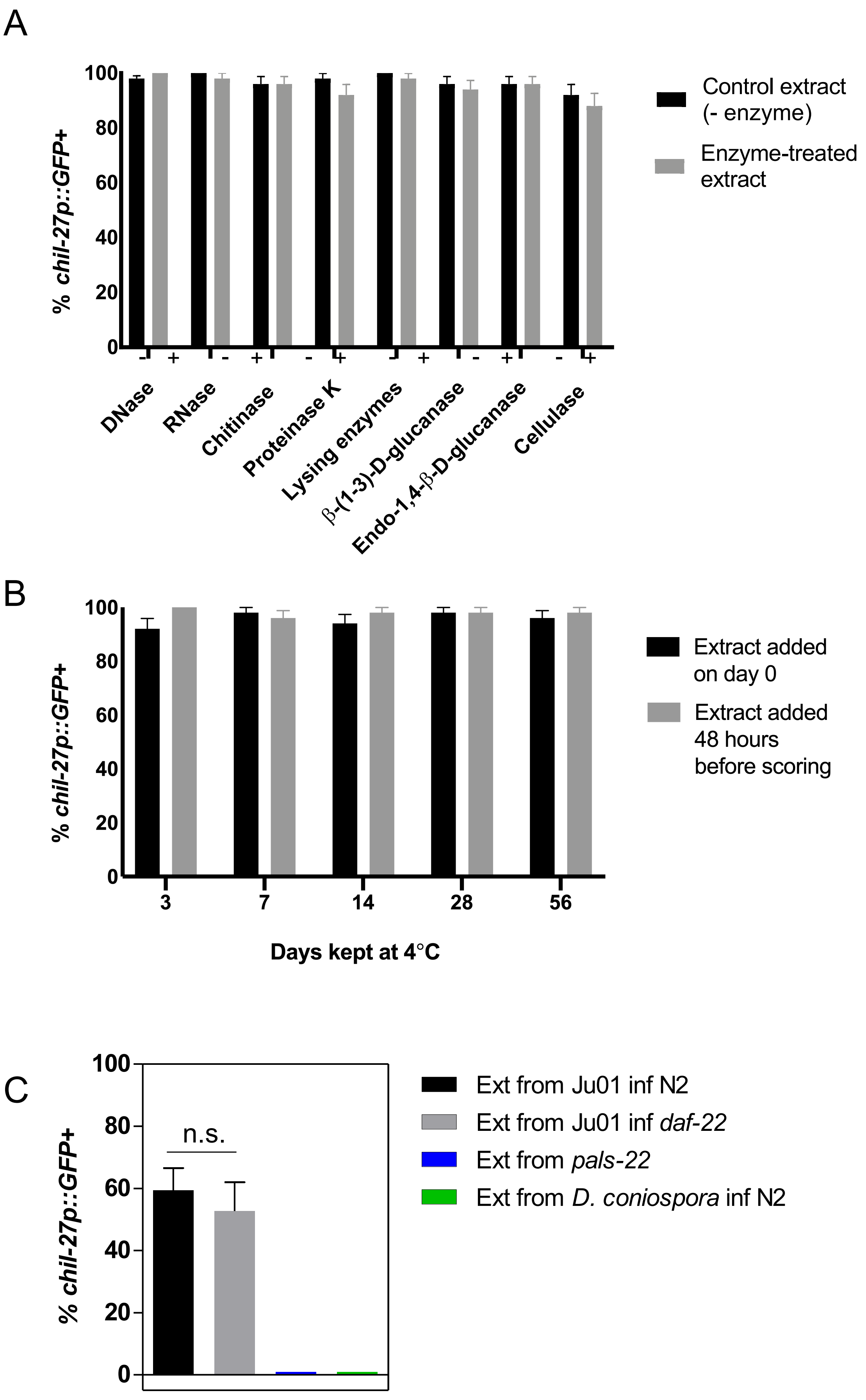
A
% chil-27p::GFP+
100
80
60
40
20
0
- + - + - + - + - + - + - + - +
DNase
RNase
Chitinase
Proteinase K
Lysing enzymes
β-(1-3)-D-glucanase
Endo-1,4-β-D-glucanase
Cellulase
Control extract (- enzyme)
Enzyme-treated extract
B
% chil-27p::GFP+
3
7
14
28
56
Days kept at 4°C
Extract added on day 0
Extract added 48 hours before scoring
C
% chil-27p::GFP+
n.s.
Ext from Ju01 inf N2
Ext from Ju01 inf daf-22
Ext from pals-22
Ext from D. coniospora inf N2

Figure S1

Figure S1: Determining the chemical identity, stability and source of the active molecule in the pathogen extract. Related to Figure 1.

(A) Treatment with various degrading enzymes does not reduce the potency of the extract. Each control extract was treated with the same conditions as the enzyme-treated extract, according to manufacturer's instructions to maximise enzyme activity. Lysing enzymes include DNase, RNAse, β-glucanase, cellulase, protease, and chitinase activities. The concentration used for DNase and RNase treatment was 100 µg/ml, while 1 mg/ml was used for rest of the enzymes. Enzyme-treated (+) and control extract (-) was added to the OP50 lawn and animals were scored 48 hours later for *chil-27p::GFP* induction (n = 50, for all treatments). (B) Incubation of extract on OP50 lawn at 4°C for up to 8 weeks does not decrease its potency to induce *chil-27p::GFP*. All plates were seeded with extract on day 0 and kept at 4°C until ready for testing. The same batch of extract was used as control and was added to the OP50 lawn 48 hours before animals were scored for *chil-27p::GFP* induction (n = 50, for all treatments). (C) Response to small-scale extracts (Ext) prepared from *M. humicola*-infected N2, *daf-22(m130)*, *D. coniospora*-infected N2 and uninfected *pals-22(icb89)* animals. No response was seen for *pals-22(-)* and *D. coniospora* and comparable response was found between N2 and *daf-22(-)* mutants. Error bars in A-C indicate standard error of the proportion.

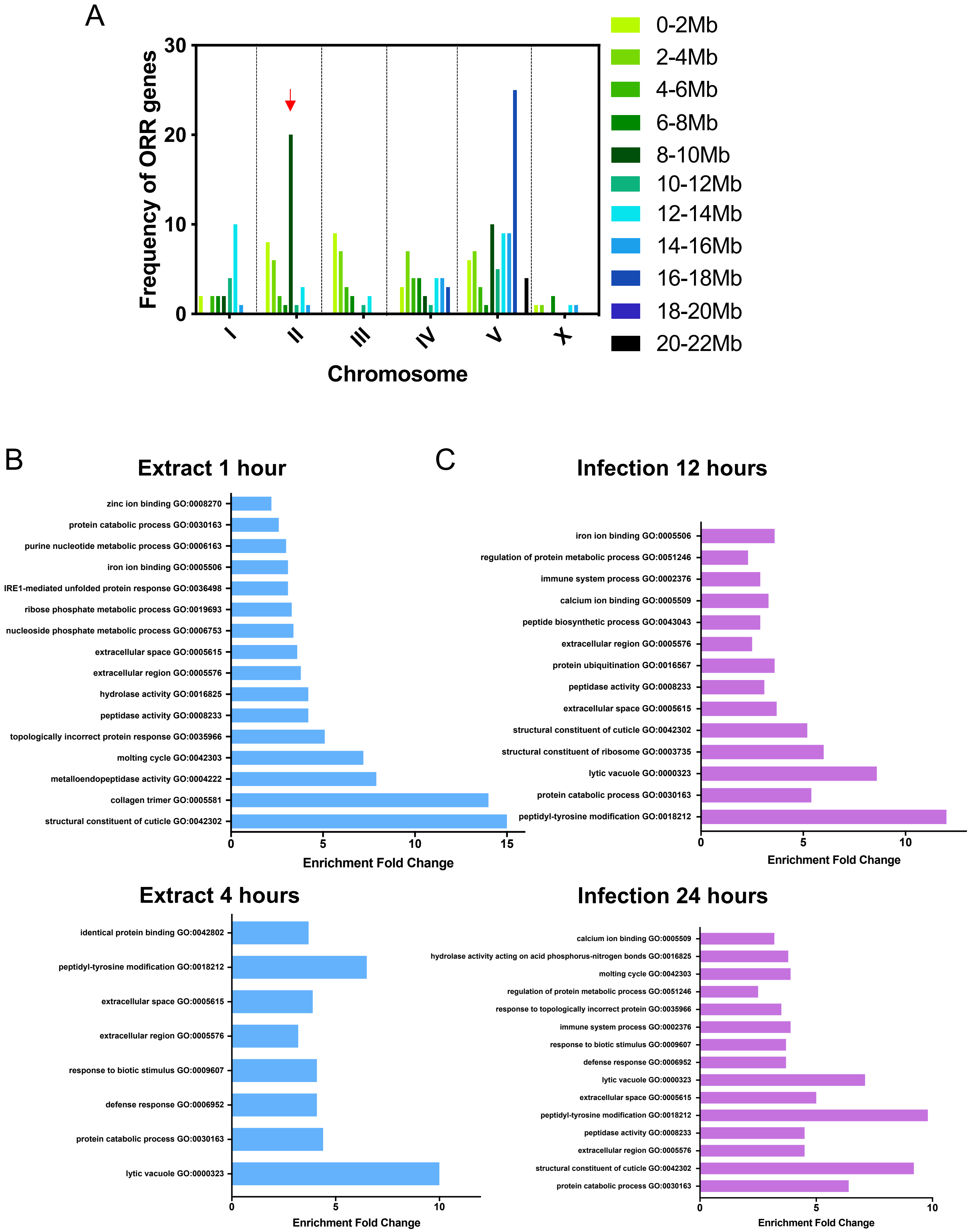
A
Frequency of ORR genes
30
20
10
0
I
II
III
IV
V
X
Chromosome
0-2Mb
2-4Mb
4-6Mb
6-8Mb
8-10Mb
10-12Mb
12-14Mb
14-16Mb
16-18Mb
18-20Mb
20-22Mb
B
Extract 1 hour
zinc ion binding GO:0008270
protein catabolic process GO:0030163
purine nucleotide metabolic process GO:0006163
iron ion binding GO:0005506
IRE1-mediated unfolded protein response GO:0036498
ribose phosphate metabolic process GO:0019693
nucleoside phosphate metabolic process GO:0006753
extracellular space GO:0005615
extracellular region GO:0005576
hydrolase activity GO:0016825
peptidase activity GO:0008233
topologically incorrect protein response GO:0035966
molting cycle GO:0042303
metalloendopeptidase activity GO:0004222
collagen trimer GO:0005581
structural constituent of cuticle GO:0042302
0
5
10
15
Enrichment Fold Change
C
Infection 12 hours
iron ion binding GO:0005506
regulation of protein metabolic process GO:0051246
immune system process GO:0002376
calcium ion binding GO:0005509
peptide biosynthetic process GO:0043043
extracellular region GO:0005576
protein ubiquitination GO:0016567
peptidase activity GO:0008233
extracellular space GO:0005615
structural constituent of cuticle GO:0042302
structural constituent of ribosome GO:0003735
lytic vacuole GO:0000323
protein catabolic process GO:0030163
peptidyl-tyrosine modification GO:0018212
0
5
10
Enrichment Fold Change
Extract 4 hours
identical protein binding GO:0042802
peptidyl-tyrosine modification GO:0018212
extracellular space GO:0005615
extracellular region GO:0005576
response to biotic stimulus GO:0009607
defense response GO:0006952
protein catabolic process GO:0030163
lytic vacuole GO:0000323
0
5
10
Enrichment Fold Change
Infection 24 hours
calcium ion binding GO:0005509
hydrolase activity acting on acid phosphorus-nitrogen bonds GO:0016825
molting cycle GO:0042303
regulation of protein metabolic process GO:0051246
response to topologically incorrect protein GO:0035966
immune system process GO:0002376
response to biotic stimulus GO:0009607
defense response GO:0006952
lytic vacuole GO:0000323
extracellular space GO:0005615
peptidyl-tyrosine modification GO:0018212
peptidase activity GO:0008233
extracellular region GO:0005576
structural constituent of cuticle GO:0042302
protein catabolic process GO:0030163
0
5
10
Enrichment Fold Change

Figure S2

Figure S2: Distribution and gene ontology enrichment analysis of upregulated ORR genes. Related to Figure 2.

(A) Graph showing induced ORR genes per chromosome in bins of 2Mb. Genes on chromosome V are significantly overrepresented with a binomial test (*p* value<0.001). Local peaks in the frequency of induced genes along the chromosomes may correspond to response clusters, for example the most prominent peak (indicated by a red arrow) on chromosome II (8-10 Mb bin) includes many *chil* genes. (B-C) Gene ontology enrichment analysis of upregulated genes identified at different timepoints. Two time points are shown for pathogen extract treatment (B) and infection (C). All datasets have been analysed using the enrichment analysis tool on Wormbase.

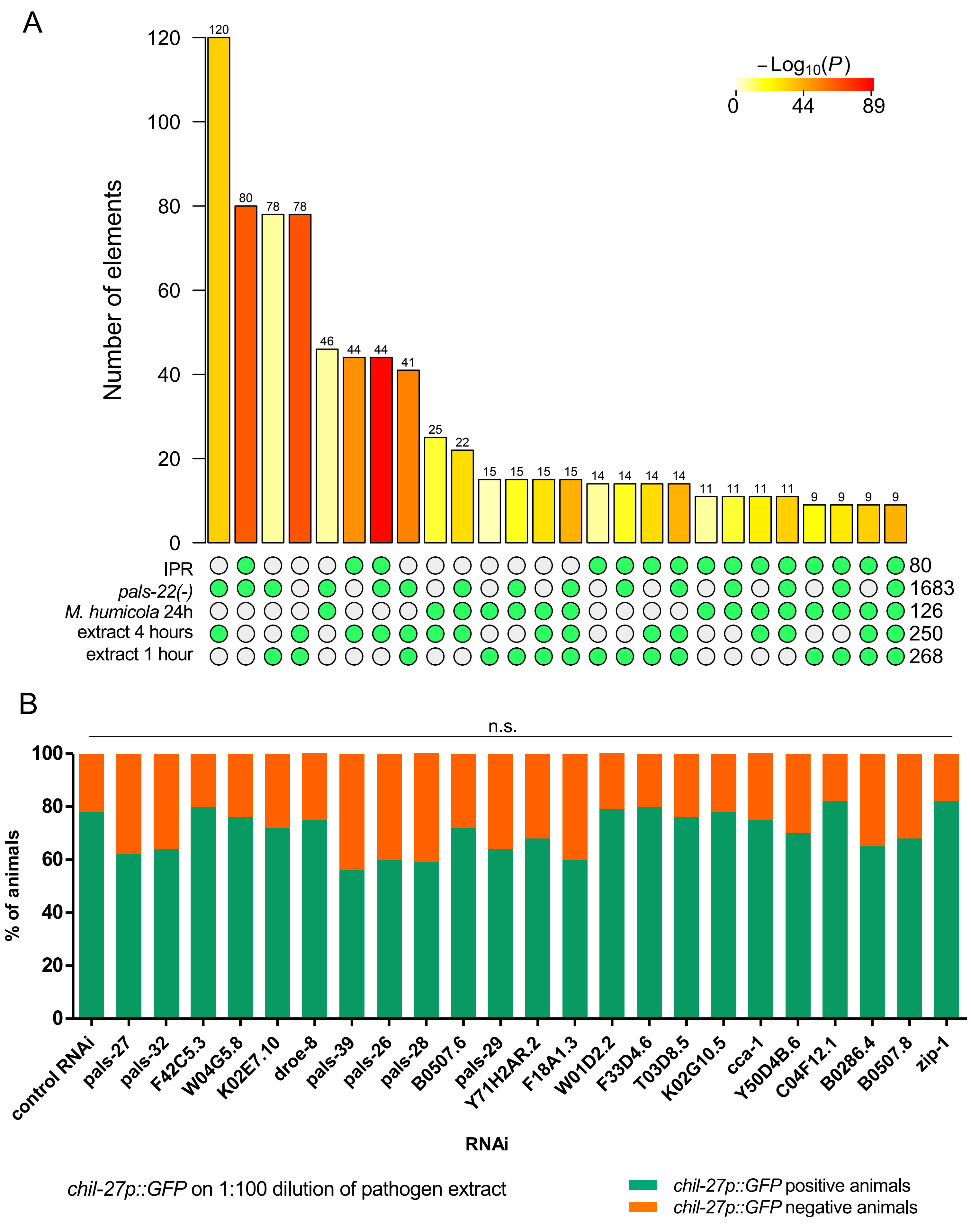
A
Number of elements
–Log10(P)
0
44
89
IPR
pals-22(-)
M. humicola 24h
extract 4 hours
extract 1 hour
80
1683
126
250
268
B
n.s.
% of animals
RNAi
control RNAi
pals-27
pals-32
F42C5.3
W04G5.8
K02E7.10
droe-8
pals-39
pals-26
pals-28
B0507.6
pals-29
Y71H2AR.2
F18A1.3
W01D2.2
F33D4.6
T03D8.5
K02G10.5
cca-1
Y50D4B.6
C04F12.1
B0286.4
B0507.8
zip-1
chil-27p::GFP on 1:100 dilution of pathogen extract
chil-27p::GFP positive animals
chil-27p::GFP negative animals

Figure S3

Figure S3: Analysis of the transcriptional response to pathogen extract exposure. Related to Figure 3.

(A) Overlap between different gene sets included in the GSEA analysis. Bars with numbers on top show the overlap of specific gene sets depicted in green. The size of each gene set appears on the bottom right. Key shows *p* value based on Fisher's exact test (Wang et al., 2015). (B) RNAi screen targeting ORR genes and studying their impact on *chil-27p::GFP* induction. No significant changes were found.

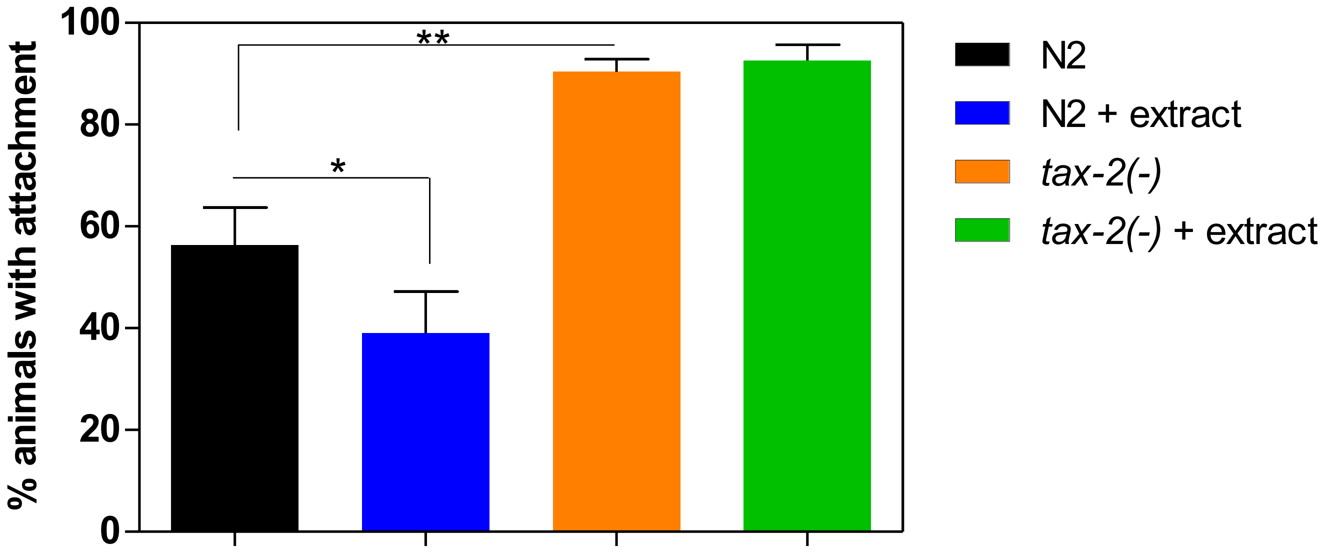

100
80
60
40
20
0
% animals with attachment
**
*
N2
N2 + extract
tax-2(-)
tax-2(-) + extract

Figure S4

Figure S4: Pathogen attachment assay for *tax-2(p691)* mutant animals. Related to Figure 5.

(A) Percentage of N2 and *tax-2(p691)* animals with and without pretreatment with extract showing oomycete attachment after 4h exposure with the pathogen at 20°C (** $p<0.01$ and * $p<0.05$ with Chi-square test, n=50).

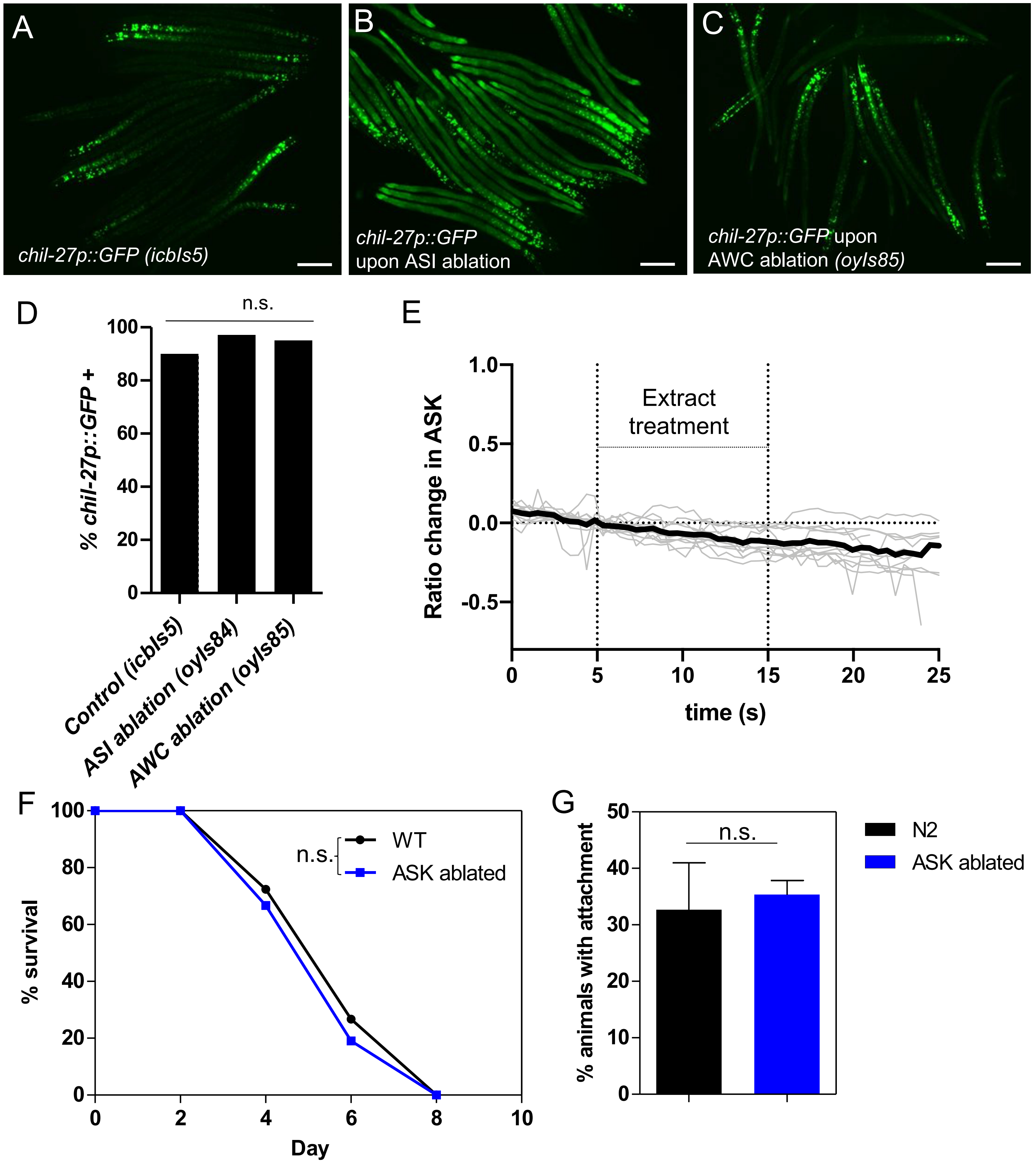

A
chil-27p::GFP (icbIs5)
B
chil-27p::GFP
upon ASI ablation
C
chil-27p::GFP upon
AWC ablation (oyIs85)
D
n.s.
% chil-27p::GFP +
100
80
60
40
20
0
Control (icbIs5)
ASI ablation (oyIs84)
AWC ablation (oyIs85)
E
Extract
treatment
Ratio change in ASK
1.0
0.5
0.0
-0.5
0
5
10
15
20
25
time (s)
F
100
80
60
40
20
0
% survival
n.s.
WT
ASK ablated
0
2
4
6
8
10
Day
G
50
40
30
20
10
0
% animals with attachment
n.s.
N2
ASK ablated

Figure S5

Figure S5: Calcium response in ASK upon oomycete extract treatment and response to extract and infection in animals with specific neurons ablated. Related to Figure 5.

(A-D) Response to oomycete extract in not impaired in strains with ASI or AWC neurons ablated. Induction assay showing *chil-27p::GFP* expression in control carrying the transgene (A), ASI ablated *(oyIs84)* (B) and AWC ablated *(oyIs85)* strains (C) in response to treatment with 1:100 dilution of oomycete extract. Neuron ablation is performed in these strains by driving split caspase expression under *gpa-4/gcy-27* promoters in (B) and a *ceh-36* promoter in (C). Scale bar is 200 μm. (D) Quantification of the *chil-27p::GFP* induction in response to extract treatment (n>30). (E) Calcium traces (n=12) over time and upon delivery of pathogen extract in ASK neurons using transgene *ljEx1186[sra-9p::GCaMP3::SL2-tagRFP]*. GCaMP3 and tagRFP intensities were measured as the mean pixel intensity of the 100 brightest pixels in a circular region of interest (ROI) with a 14 pixel radius. Calcium traces were computed as the change in the GCaMP3/tagRFP ratio (R) from the baseline value defined as the mean R prior to stimulus onset. Note no increase in R upon exposure to extract from time 5-15sec. (F) Survival curve of N2 and ASK ablated animals *(qrIs2)* at 20°C in the presence of *M. humicola* JUo1 (n.s., *p*>0.05, log-rank test, n=60 per condition). (G) Percentage of N2 and ASK ablated animals showing oomycete attachment after 4h exposure with the pathogen at 20°C (n.s., *p*>0.05, Chi-square test, n=40).
