# Supplementary material for: Chemosensory Neurons Modulate the Response to Oomycete Recognition in Caenorhabditis elegans
Source: Cell Rep. 2021 Jan 12;34(2):108604. doi: 10.1016/j.celrep.2020.108604 (PMC7809619; doi:10.1016/j.celrep.2020.108604)
Supplement: Document S2. Article plus Supplemental Information [file mmc8.pdf]

Cell Reports

Article

# Chemosensory Neurons Modulate the Response to Oomycete Recognition in *Caenorhabditis elegans*

## Graphical Abstract

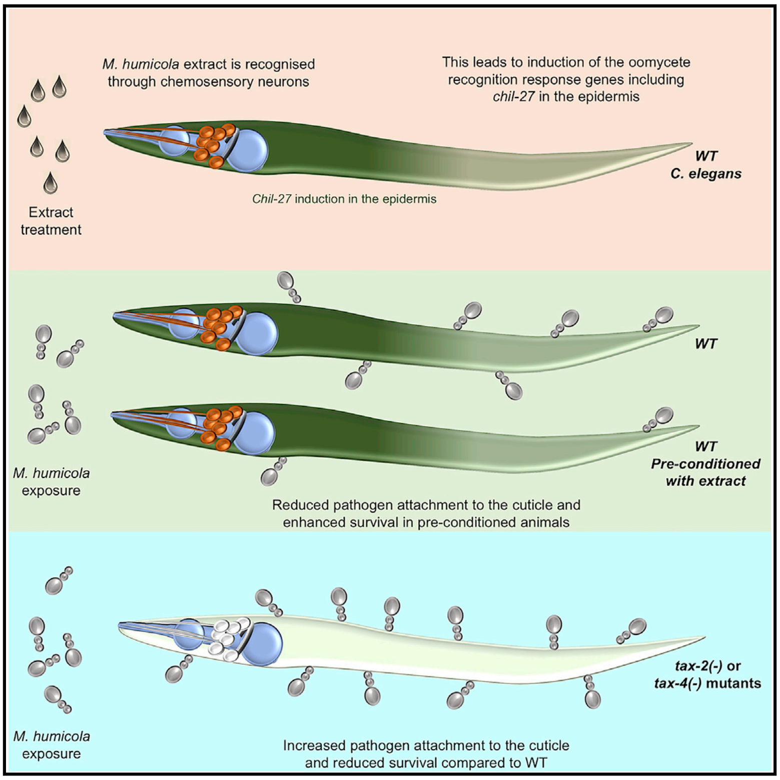


## Authors

Michael K. Fasseas, Manish Grover, Florence Drury, Clara L. Essmann, Eva Kaulich, William R. Schafer, Michalis Barkoulas

## Correspondence

m.barkoulas@imperial.ac.uk

## In Brief

The oomycetes include key eukaryotic pathogens of animals and plants, but animal-oomycete interactions are little explored. Fasseas et al. report that *C. elegans* has evolved appropriate means to sense its natural oomycete pathogen *Myzocytiopsis humicola* and mount anticipatory defense response in the epidermis to protect from an upcoming infection threat.

## Highlights

- *C. elegans* senses its natural oomycete pathogen *M. humicola* without infection
- Exposure to a pathogen extract triggers an oomycete recognition response
- Upon pathogen detection, *C. elegans* resists infection through changes in the cuticle
- The response involves signaling between sensory neurons and the epidermis

Fasseas et al., 2021, Cell Reports *34*, 108604
January 12, 2021 © 2020 The Authors.
https://doi.org/10.1016/j.celrep.2020.108604

CellPress

Cell Reports

CellPress
OPEN ACCESS

Article

# Chemosensory Neurons Modulate the Response to Oomycete Recognition in *Caenorhabditis elegans*

Michael K. Fasseas,[1,3] Manish Grover,[1,3] Florence Drury,[1] Clara L. Essmann,[1] Eva Kaulich,[2] William R. Schafer,[2] and Michalis Barkoulas[1,4,*]
[1]Department of Life Sciences, Imperial College, London SW7 2AZ, UK
[2]MRC Laboratory of Molecular Biology, Cambridge CB2 0QH, UK
[3]These authors contributed equally
[4]Lead Contact
*Correspondence: m.barkoulas@imperial.ac.uk
https://doi.org/10.1016/j.celrep.2020.108604

## SUMMARY

Understanding how animals detect and respond to pathogen threats is central to dissecting mechanisms of host immunity. The oomycetes represent a diverse eukaryotic group infecting various hosts from nematodes to humans. We have previously shown that *Caenorhabditis elegans* mounts a defense response consisting of the induction of *chitinase-like* (*chil*) genes in the epidermis to combat infection by its natural oomycete pathogen *Myzocytiopsis humicola*. We provide here evidence that *C. elegans* can sense the oomycete by detecting an innocuous extract derived from animals infected with *M. humicola*. The oomycete recognition response (ORR) leads to changes in the cuticle and reduction in pathogen attachment, thereby increasing animal survival. We also show that TAX-2/TAX-4 function in chemosensory neurons is required for the induction of *chil-27* in the epidermis in response to extract exposure. Our findings highlight that neuron-to-epidermis communication may shape responses to oomycete recognition in animal hosts.

## INTRODUCTION

Organisms can use multiple strategies to protect themselves from pathogens in their natural environment. These strategies can involve avoiding contact with the pathogen or resisting infection by mounting appropriate immune responses (Akira et al., 2006; Singh and Aballay, 2020). Innate immune responses can be both pathogen or host specific and can be generalized into two main types based on how they are initiated. Pathogen-associated molecular patterns (PAMPs) can act as signals for dedicated host receptors that then activate an intracellular cascade of events leading to the production of defense molecules (Akira et al., 2006). Alternatively, damage-associated molecular patterns (DAMPs) associated with perturbations of host physiology or tissue damage can also trigger the activation of innate immune signaling (Hou et al., 2019; Pujol et al., 2008). In both cases, pathogen detection, before and during infection, provides a strategy for organisms to strengthen the efficacy and specificity of their defense response (Mogensen, 2009).

The nematode *Caenorhabditis elegans* has been extensively used as a model for studying host-pathogen interactions for both naturally occurring (Schulenburg and Félix, 2017) and lab-induced (Sifri et al., 2003; Tan et al., 1999) infections. The former category is particularly useful to uncover host immune responses that have evolved to combat nematode infections in the wild, while the latter category may offer more direct links to biomedical challenges. Based on these infection models, various signaling pathways and immune effectors have been described to play a role in *C. elegans* defense (Ermolaeva and Schumacher, 2014; Ewbank and Pujol, 2016; Kim and Ewbank, 2018). Nevertheless, the characterization of the molecular pathways that underlie detection of various pathogens in *C. elegans* remains largely elusive (Meisel et al., 2014; Pradel et al., 2007; Pukkila-Worley et al., 2011; Twumasi-Boateng and Shapira, 2012).

We recently reported the identification of the oomycete *Myzocytiopsis humicola* as a natural pathogen of *C. elegans* (Osman et al., 2018). Oomycetes represent an evolutionarily distinct lineage of eukaryotes sharing morphological similarities with fungi but phylogenetic space with brown algae and diatoms in the Stramenopiles (Beakes et al., 2012). This group includes the crop pathogen *Phytophthora infestans*, infamous for the Irish famine in the mid-19$^{th}$ century due to shortage of potatoes caused by the late blight disease (Fry, 2008). Although oomycetes have been mostly known as plant pathogens, they can also cause human infections, such as those caused by the oomycete *Pythium insidiosum* (De Cock et al., 1987; Krajaejun et al., 2006). The resulting animal disease, known as pythiosis or "swamp cancer," is considered an emerging disease with significant mortality, especially in the tropics (Mendoza and Newton, 2005). The recent identification of oomycetes as natural pathogens of *C. elegans* offers an attractive model system to mechanistically study and dissect animal-oomycete interactions.

In contrast to bacterial pathogens that most often infect from the gut, *M. humicola* enters through the cuticle to rapidly kill


This is an open access article under the CC BY license (http://creativecommons.org/licenses/by/4.0/).

*C. elegans* (Osman et al., 2018). In this study, we make use of this infection model to explore aspects of oomycete recognition in animals. We demonstrate that *C. elegans* can detect the oomycete, even in the absence of infection. We characterize in detail the transcriptional response of *C. elegans* to oomycete recognition and uncouple it from the response to tissue damage that occurs during infection. We provide evidence that *C. elegans* exposed to a non-infectious pathogen extract becomes less susceptible to oomycete infection through changes in the cuticle that reduce pathogen attachment. Interestingly, the response to extract and its protective effect is alleviated when chemosensation in sensory neurons is compromised. This work highlights the ability of *C. elegans* to sense its natural oomycete pathogen *M. humicola* and initiate anticipatory defense responses that reduce the chance of infection.

## RESULTS

### *C. elegans* Can Sense Its Natural Oomycete Pathogen *M. humicola*

We previously reported that *chitinase-like* (*chil*) gene induction is a hallmark of the *C. elegans* defense response to oomycete exposure and that this response is triggered upon exposure to both live and autoclaved *M. humicola* (Osman et al., 2018). This observation led us to hypothesize that *C. elegans* may be able to sense the oomycete pathogen to initiate defense responses that precede infection. To test this hypothesis, we decided to evaluate whether a pathogen extract derived from infected nematodes could also trigger a host defense response. We devised a method to produce an aqueous extract from infected populations of *C. elegans* that is non-infectious yet capable of inducing the expression of a transcriptional *chil-27p::GFP* marker (Figure 1A). This method consists of washing culture plates in which the infection has progressed with sterile water in parallel with plates that do not contain pathogen as a control. The derived pathogen extract was purified by centrifugation and filtration of the supernatant before application to nematode growth media. Efficacy was evaluated by performing serial dilution experiments and quantifying the dose-dependent induction of the *chil-27p::GFP* marker. Interestingly, we found that the pathogen extract is potent and can be diluted up to 500 times, while retaining more than 50% activity (Figure 1B). We also found that it is heat stable and maintains its activity after autoclaving (Figure 1B) as well as upon treatment with various degrading enzymes, such as β-glucanases, proteinases, and chitinases, among others (Figure S1A). Finally, the extract is stable over a long time and retains full activity with or without the *Escherichia coli* food source at 4°C for at least 2 months (Figure S1B). We observed in multiple attempts that animals expressing the *chil-27p::GFP* marker upon exposure to extract could not transmit the response when introduced to populations that had not been previously exposed. We conclude that the oomycete extract may contain a pathogen-derived molecule as its active component, which is both stable and abundant in our infected nematode cultures.

We next used the pathogen extract to explore the temporal dynamics of *chil-27p::GFP* induction. Using single molecule fluorescence *in situ* hybridization (smFISH), we could detect *chil-27* mRNAs induced in the epidermis (known as "hypodermis" in *C. elegans*) as early as 1 h after extract exposure (Figure 1C). Interestingly, *chil-27* transcripts localized first to the anterior side of the syncytial hypodermis, which is also reminiscent of the activation pattern of the *chil-27p::GFP* transcriptional marker (Figure 1A). Taken together, these results suggest that the pathogen extract and *chil-27p::GFP* induction represent a robust trigger and readout of pathogen detection, thereby offering a powerful system to dissect mechanisms of oomycete recognition using *C. elegans*.

### Exposure to Pathogen Extract Triggers an ORR in the Host

We next sought to determine how the *C. elegans* transcriptional response to extract exposure may compare with infection with live pathogen. We reasoned that this experiment would allow us to uncouple responses underlying oomycete recognition from those attributed to tissue damage during infection. Our previous experiments indicated that *chil-27* is rapidly induced within the first hour of extract exposure, while it takes 24–48 h for the infection phenotype to appear, as manifested by the development of sporangia within the animal body (Osman et al., 2018). Therefore, we chose to include in the design of RNA sequencing (RNA-seq) experiments both early (1 and 4 h) and late (12, 24, and 48 h) time points post extract or pathogen treatment in parallel to control treatments with no exposure to extract or pathogen (Figure 2A). We found a strong transcriptional response to extract 1 and 4 h post exposure, which became much less detectable at 12 or 24 h (Table S1). For example, 268 genes were induced at 1 h and 250 genes at 4 h, with 78 genes being in common between the two time points. Among the induced genes, several members of the *chil* gene family were identified (Figure 2B). A total of 96 genes were found to be downregulated at 1 h and 109 genes at 4 h post extract treatment, but the overlap between time points was smaller, consisting of 16 genes in total (Table S1).

To define what may constitute an oomycete recognition response (ORR), we compared the infection and extract datasets by pooling all time points. Given that pathogen detection is part of the infection process, we anticipated that genes differentially regulated upon extract treatment would show a significant overlap with those identified upon exposure to live pathogen. Indeed, of 452 genes found to be upregulated in response to extract treatment by pooling all time points, 150 genes were also induced during infection with *M. humicola* (Figure 2C). We argued that genes detected exclusively upon extract treatment may still be part of a genuine pathogen recognition response. These genes may have been missed in the infection experiments due to undersampling of the time course of infection or because infection could mask the recognition process. Therefore, we decided to consider as strict ORR genes those that were common between extract treatment and infection as well as those uniquely found in the extract datasets if they showed a high magnitude of change (fold change $\geq 2$ or $\leq -2$) (Pimentel et al., 2017). The final ORR list consisted of 206 upregulated genes and 49 downregulated genes, a number of which were found to be conserved in humans (69/206 and 22/49 genes, respectively; Table S2) (Kim et al., 2018).

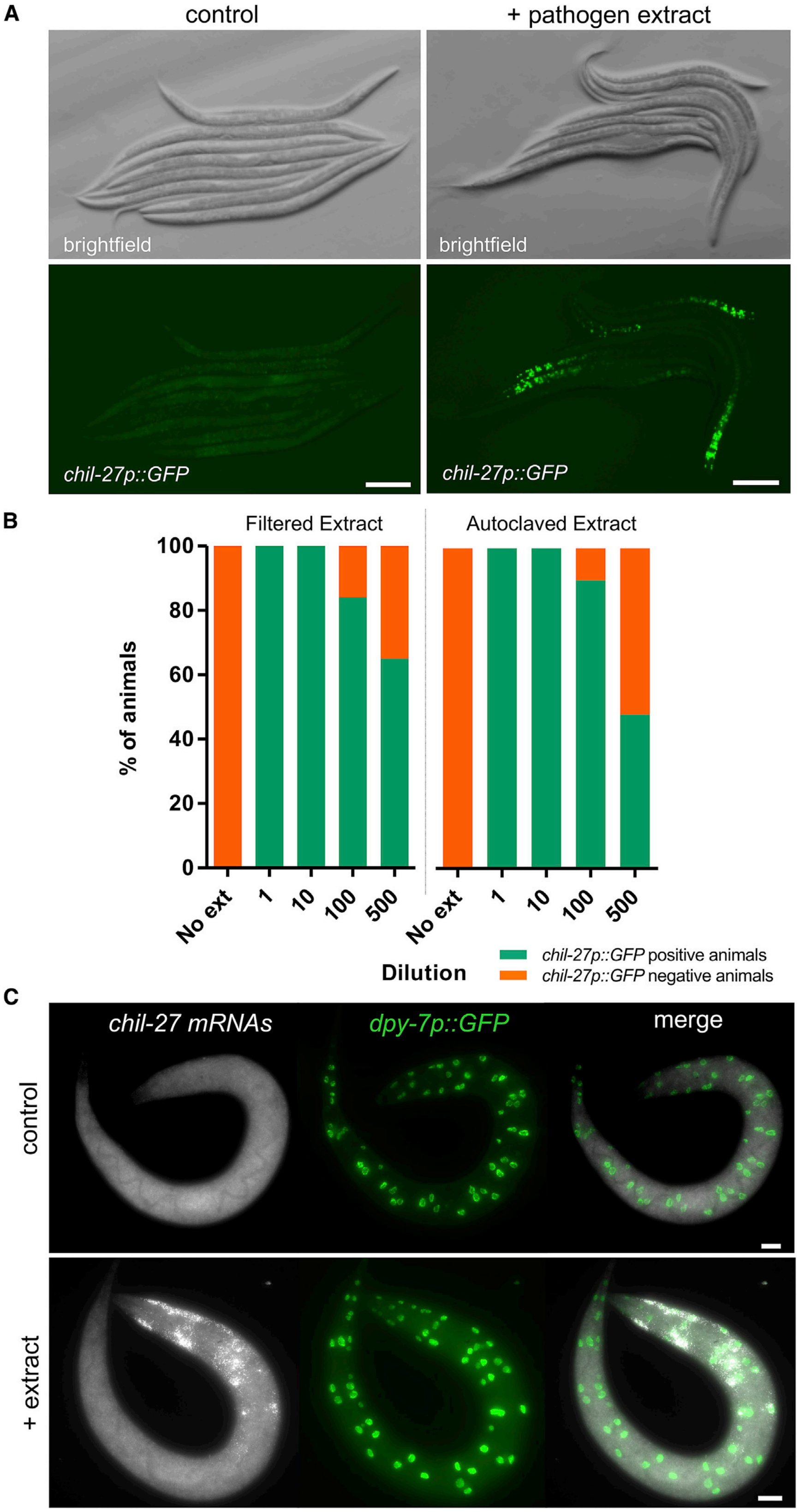


**Figure 1. An Extract from *M. humicola*-Infected Nematodes Can Induce *chil-27p::GFP* Expression in *C. elegans* without Infection**

(A) A non-infectious extract made by washing infected plates with water leads to 100% *chil-27p::GFP* induction in the population. Control here refers to extract made from plates with no oomycete infection, and this treatment does not result in *chil-27p::GFP* expression (0% induction, n > 100).

(B) Representative *chil-27p::GFP* induction assay upon dilution of filtered pathogen extract versus autoclaved extract. No significant difference is found between the two treatments.

(C) Expression of *chil-27* by smFISH upon pathogen extract exposure. Image shows a second larval stage animal 1 h post extract or control treatment. A *dpy-7p::GFP* marker is used to visualize hypodermal nuclei. Note co-localization of *chil-27* mRNAs with hypodermal nuclei (hyp6 and anterior hyp7). Scale bars: 100 μm in (A) and 20 μm in (C).

See also Figure S1.

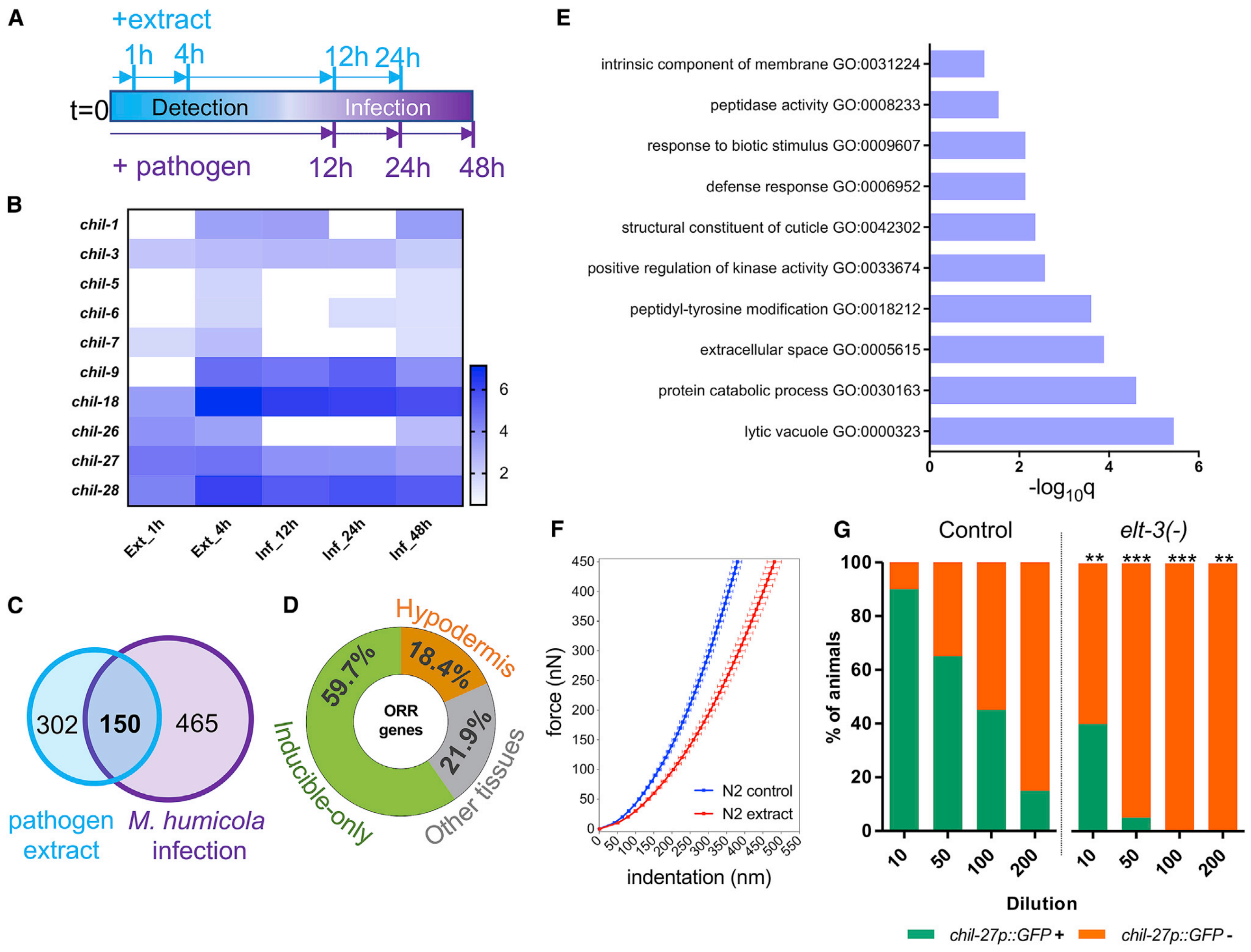


**Figure 2. Transcriptional Response to Pathogen Extract Defines an Oomycete Recognition Response**

(A) Cartoon summarizing the design of the RNA-seq experiment. N2 animals at the L4 stage were exposed to pathogen extract for 1, 4, 12, and 24 h or to live pathogen for 12, 24, and 48 h, respectively.

(B) Heatmap showing the members of the *chil* gene family that are differentially expressed by pathogen extract and infection. Heatmap color is based on Sleuth *b* values, which are analogous to fold change (Pimentel et al., 2017). White color indicates that the gene was not significantly upregulated in that condition (significance is defined as p value < 0.01 and FDR-adjusted p value < 0.1).

(C) Venn comparison showing the overlap of differentially expressed genes by extract and *M. humicola* infection (pooled time points). This overlap is significant with a representation factor (RF) 8.3 and p value $< 2.858 \times 10^{-99}$ with a hypergeometric test.

(D) Classification of upregulated ORR genes. A major fraction of these genes (n = 117) are only inducible upon extract treatment: 36 genes are reported to be expressed in the hypodermis, while 43 genes are not expressed in the hypodermis in wild type based on a threshold of <5 transcripts per million (Cao et al., 2017a). Pseudogenes have been removed, so 196 genes have been used in this analysis.

(E) Gene Ontology (GO) enrichment analysis for the upregulated ORR genes using WormBase. Adjusted p value is shown on the x axis, and GO terms are listed on the y axis.

(F) Force-displacement curves using atomic force microscopy. N2 animals treated with extract show reduction in stiffness, n > 20 animals per genotype.

(G) Induction of *chil-27p::GFP* in *elt-3(gk121)* versus control (***p value < 0.001 and **p value < 0.01 with a chi-square test for each independent dilution, n > 50 for each dilution).

See also Figure S2 and Tables S1, S2, and S3.

Most ORR genes were differentially expressed 4 h post extract exposure (183/206 induced and 34/49 downregulated genes) (Table S2). ORR genes were present on all chromosomes, but induced genes were overrepresented on chromosome V (Figure S2A). ORR genes were found to be primarily inducible genes (57% of the genes; Figure 2D) with minimal expression (a threshold of <5 transcripts per million) detected in wild-type tissues based on published single-cell RNA-seq (Table S2) (Cao et al., 2017a). An interesting feature of the ORR list is that it contained many genes expressed mostly outside the hypodermis (Figure 2D; Table S2), which may indicate systemic features in the response. However, this does not rule out that ORR genes get induced in the hypodermis upon oomycete exposure as part of a pathogen-specific recognition program.

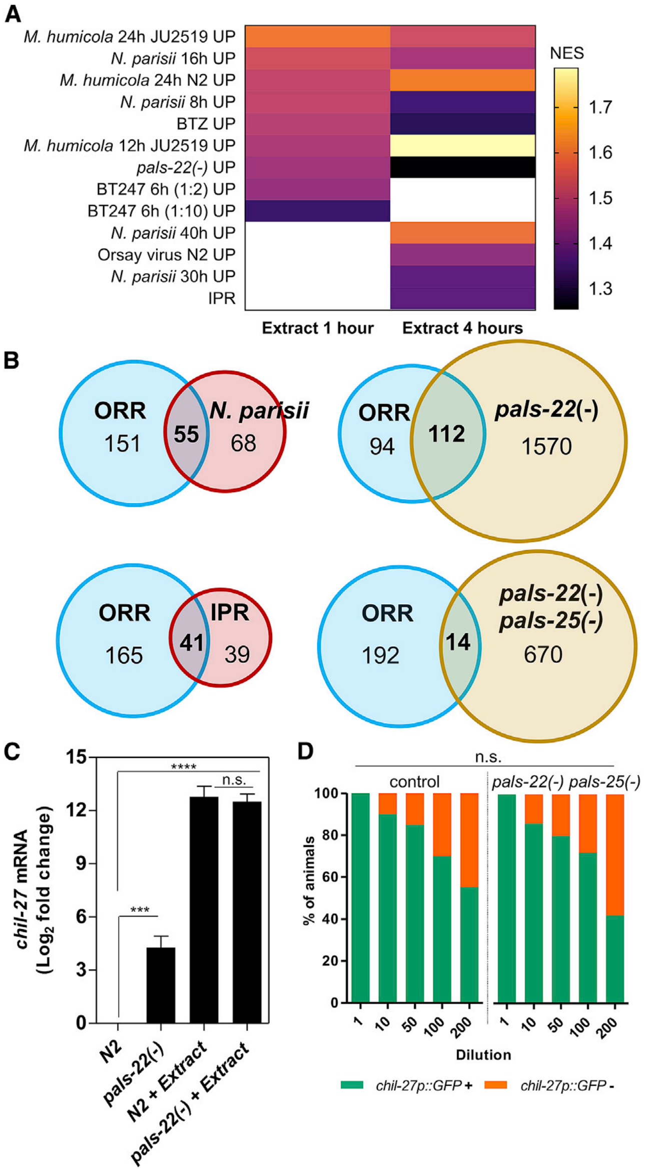


**Figure 3. Comparative Analysis of the Oomycete Recognition Response**

(A) Heatmap presenting the normalized enrichment score (NES) derived from GSEA analysis and focusing on all gene sets showing significant intersection with our pathogen extract datasets. White color depicts no significant intersection (FDR < 0.25 and nominal p value < 0.05).

(B) Venn diagrams comparing the upregulated ORR genes versus IPR (RF: 38.1, $p < 1.892 \times 10^{-56}$), *N. parisii* infection at 8 h (RF: 33.3, $p < 9.031 \times 10^{-72}$), and *pals-22(jy3)* (RF: 5, $p < 1.070 \times 10^{-53}$) and *pals-22(jy3) pals-25(jy9)* double mutants (RF: 1.5, non-significant). RNA-seq data used here are from Reddy et al. (2019).

(C) qRT-PCR for *chil-27* expression in *pals-22(icb89)* treated with extract and N2 treated with extract (p < 0.001, one-way ANOVA and Tukey's multiple comparison test) and N2 upon extract treatment.

(D) *pals-22(icb90) pals-25(icb92)* double mutants respond to extract just like N2 at all extract dilutions (results are non-significant with a chi-square test for each independent dilution, n > 50 for each dilution).

See also Figure S3 and Table S4.

We used Gene Ontology (GO) enrichment analysis (Angeles-Albores et al., 2016; Ge et al., 2019; Raudvere et al., 2019) to further characterize the obtained datasets upon extract treatment, as well as the derived ORR list (Figure 2E; Figures S2B and S2C; Table S3). The GO term chitin binding was found for all datasets by ShinyGo and g:Profiler, and this reflects the induction of several *chil* genes. We found enrichment of a cuticle composition term for the dataset obtained 1 h post extract treatment, which suggests a link between pathogen detection and the cuticle physical barrier. Consistent with this idea, atomic force microscopy (AFM) revealed that extract treatment changed the stiffness of the cuticle (Figure 2F). GO terms for defense response and response to a biotic stimulus were found 4 h post extract treatment (Figure 2E; Table S3). Other GO terms related to immunity, such as molecular degradation (e.g., protein catabolic processes), as well as cell signaling (e.g., transmembrane receptor kinase activity), were also found at 4 h (Figure 2E; Table S3) and may reflect pathways involved in the response to pathogen recognition. Finally, transcription factor binding analysis upstream of ORR genes using g:Profiler identified the hypodermally expressed GATA transcription factor ELT-3 as a putative regulator of the response (Table S3). We tested this prediction and found that *elt-3(gk121)* loss-of-function mutants are partially impaired in the induction of *chil-27p::GFP* in the hypodermis (Figure 2G).

To compare the response to oomycete extract exposure with the response against other known pathogens of *C. elegans*, we performed gene set enrichment analysis (GSEA) (Mootha et al., 2003; Subramanian et al., 2005). We found that datasets representing extract exposure for 1 and 4 h had significant intersections (nominal [NOM] p value < 0.05 and false discovery rate [FDR] < 0.25) with 9 and 11 of 65 datasets, respectively (Table S4). As expected, a significant intersection was found with datasets derived from infection by *M. humicola* (Figure 3A; Figure S3A; Table S4). The remaining significant intersections involved mostly datasets derived from time points post infection by the intestinal microsporidia *Nematocida parisii* (Figure 3A). It is of note that the intracellular pathogen response (IPR) (Reddy et al., 2017), which represents part of the response to microsporidia infection in *C. elegans*, was 51% shared with ORR (Figure 3B; Figure S3A). This is interesting given that these two independently derived gene lists concern evolutionary unrelated pathogens that use different infection strategies and exhibit distinct tissue tropism.

We previously reported an overlap in the machinery regulating the transcriptional response to microsporidia and oomycete infections (Reddy et al., 2019). This is because loss-of-function mutations in the protein containing ALS2CR12 signature (PALS) gene *pals-22* lead to constitutive *chil-27p::GFP* induction in the epidermis as well as induction of host responses against microsporidia in the intestine (Reddy et al., 2019). IPR and *chil-27p::GFP* induction rely on another member of the PALS family, PALS-25, as they are both suppressed in a *pals-22 pals-25* double-mutant background (Reddy et al., 2019). We therefore tested whether the ORR shares similarities with genes induced in a *pals-22* mutant background. Interestingly, 54% of induced ORR genes were also upregulated in *pals-22* mutants, while the overlap between ORR and *pals-22 pals-25* mutants was no longer significant (Figure 3B). These results indicate that PALS-25 is able to regulate

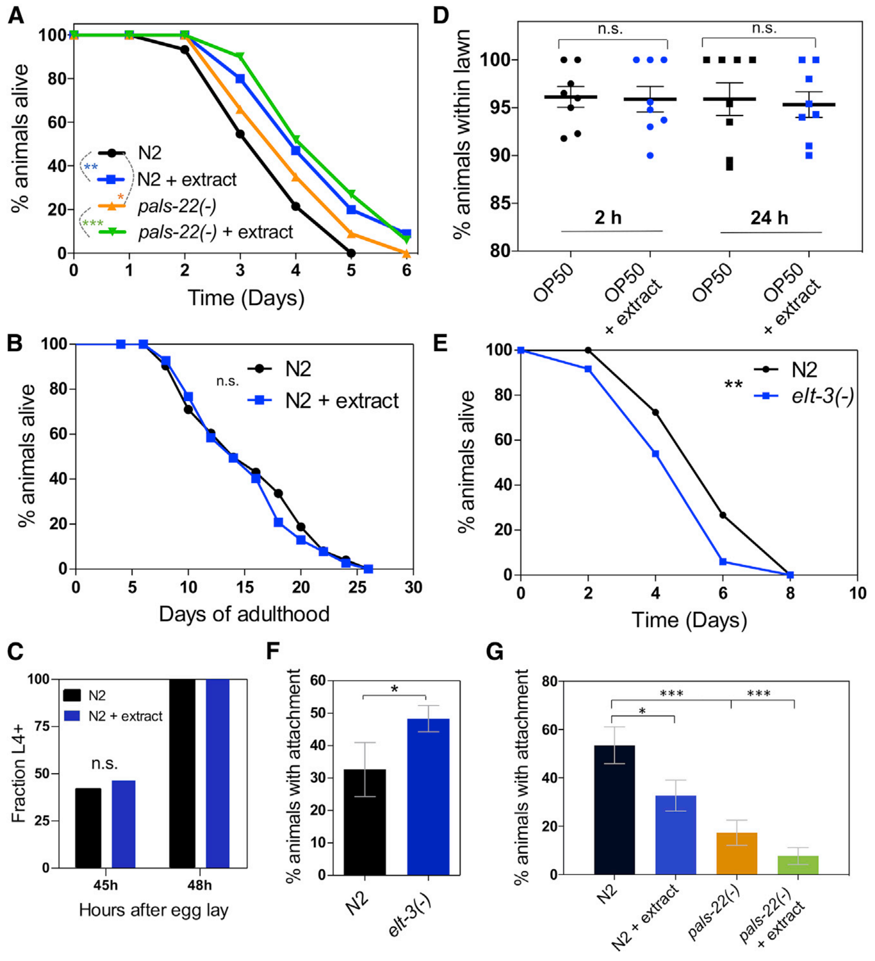


**Figure 4. Exposure of *C. elegans* to Pathogen Extract Leads to Reduced Pathogen Attachment and Provides Protection from Oomycete Infection**

(A) Infection assay comparing N2 and *pals-22(icb89)* animals with or without previous exposure to extract (***p value < 0.001, **p value < 0.01, *p value < 0.05 with a log rank test, n = 60–90 animals per condition).

(B) Life-span comparison of animals treated with extract versus non-treated controls. No significant difference was found with a log rank test.

(C) Quantification of developmental pace by measuring the fraction of animals at or beyond the L4 stage at two different time points. No difference was observed upon extract treatment with a chi-square test.

(D) Avoidance assay depicting the percentage of animals leaving the lawn in 2 and 24 h with or without adding pathogen extract.

(E) Survival curve of N2 and *elt-3(gk121)* animals at 20°C in the presence of *M. humicola* JUo1 (**p < 0.01 with log rank test, n = 60 for each).

(F) Percentage of N2 and *elt-3(−)* animals showing oomycete attachment after 4 h exposure with the pathogen at 20°C (*p < 0.05 with chi-square test, n = 40).

(G) Attachment assay for N2 and *pals-22(icb89)* animals with or without previous exposure to extract (*p value < 0.05 and ***p value < 0.001 with a chi-square test and comparisons made to N2 control, n = 50).

See also Figure S4.

a large proportion of ORR genes, when its suppression by PALS-22 is released (Reddy et al., 2019). Nevertheless, both *pals-22* and *pals-22 pals-25* mutants retained the ability to respond to extract to the same extent as control animals (Figures 3C and 3D). These results suggest that although the PALS-22/PALS-25 module can regulate ORR genes, it is unlikely to be directly involved in the response to extract perception.

The ORR gene list can serve as a signature of the host response pathway to *M. humicola* detection. It is however conceivable that the ORR list may also include genes that act, in turn, to enhance or suppress the recognition response. To test this idea, we performed a targeted RNAi screen to address whether knockdown of a subset of highly induced ORR genes would compromise *chil-27p::GFP* induction following extract treatment. We found no significant change in *chil-27p::GFP* induction in these RNAi experiments (Figure S3B). These results indicate that ORR genes are unlikely to be enriched for modulators or buffers of the response to oomycete detection.

## *C. elegans* Can Resist Infection by Detecting *M. humicola*

Pathogen recognition can provide a strategy for animals to prepare for the possibility of an upcoming infection. To test whether early detection of *M. humicola* by *C. elegans* can influence the outcome of infection, we compared the survival of animals exposed to extract before encountering the pathogen to controls that were encountering the pathogen without any prior extract treatment. We found that pretreatment with extract provided a mild, yet significant, protection from *M. humicola* infection compared with non-pretreated controls (Figure 4A). In a similar manner, *pals-22* loss-of-function mutants that show constitutive *chil-27p::GFP* expression, as well as induction of ~50% of the ORR program, were also mildly more resistant to infection (Figure 4A). Consistent with the ability of *pals-22(icb89)* mutants to still respond to extract treatment, pretreatment with extract further enhanced their protection from infection (Figure 4A). It is of note that *pals-22* loss-of-function mutants display developmental delay, as well as shortened lifespan (Reddy et al., 2017), which may affect their performance in infection assays. However, we did not find an effect on lifespan or developmental speed upon extract treatment (Figures 4B and 4C). These results highlight that detection of *M. humicola* by *C. elegans* may be beneficial as a strategy for nematode survival.

Resistance to *C. elegans* pathogens may stem from mechanisms antagonizing pathogen entry and growth or behavioral changes leading to pathogen avoidance. However, we found that animals do not avoid an *E. coli* lawn supplemented with

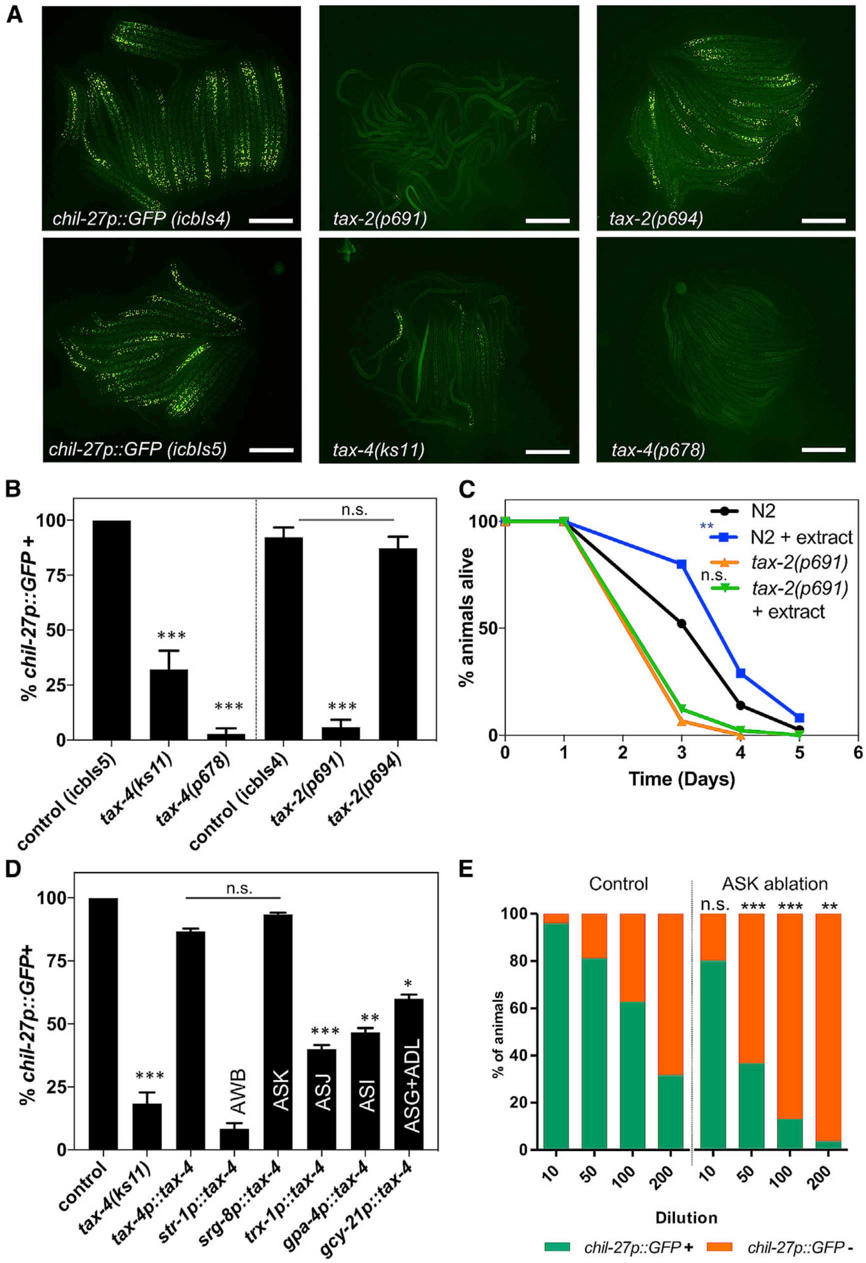


**Figure 5. Neuronal Signaling Is Required for *C. elegans* Response to Pathogen Extract**

(A) *chil-27p::GFP* transgene induction assays in strains carrying *tax-2* or *tax-4* mutations using 1:100 dilution of the pathogen extract. Images show a group animals per genotype clustered together. Scale bars, 200 μm.

(B) Quantification of induction assay for *tax-2* and *tax-4* mutants, n > 30. Note that strains carrying the *tax-2(p691)*, *tax-4(ks11)*, or *tax-4(p678)* alleles are strongly impaired in their ability to respond to extract (***p value < 0.001 with a Fisher's exact test in comparison with control). However, animals that carry the *tax-2(p694)* mutation that allows for *tax-2* expression in a subset of neurons respond normally.

(C) Infection assay comparing N2 with *tax-2(p691)* mutants, with or without pre-exposure to extract (**p value < 0.01 with a log rank test, n = 60–90 animals per condition). Note that *tax-2(p691)* is more susceptible than N2 to infection (***p value < 0.001), but extract treatment does not have any protective effect.

(D) Induction assays in strains carrying transgenes that rescue *tax-4* mutants in specific neurons. Note that rescue in ASK neurons leads to similar induction to that observed with the endogenous *tax-4* promoter, while no rescue or only partial rescue was observed in the case of other neuronal promoters (***p value < 0.001, **p value < 0.01, *p value < 0.05 with a Fisher's exact test, all comparisons are against the rescue observed using the endogenous *tax-4* promoter). Control induction in wild-type animals carrying *chil-27p::GFP* is shown as reference.

(E) Quantification of *chil-27p::GFP* induction in ASK ablated *C. elegans* (through expression of a *sra-9p::mCasp-1* transgene) in response to serial dilution of the pathogen extract. ASK-ablated strain shows significantly reduced response to extract (***p value < 0.001 and **p value < 0.01 with a chi-square test, n = 50 for each dilution).

See also Figures S5 and S6.

pathogen extract (Figure 4D). Currently, there is no evidence to suggest that *C. elegans* can clear filamentous eukaryotic pathogens when these have already penetrated the cuticle to initiate infection; thus, controlling pathogen entry into the nematode body is likely to be more crucial. We have previously proposed that induction of *chil* genes can modify the properties of the cuticle in a way that reduces pathogen attachment (Osman et al., 2018), so we focused here on whether changes in susceptibility correlate with oomycete attachment to the cuticle. As shown above, ELT-3 was predicted to regulate a part of ORR and its compromised function reduced *chil-27p::GFP* induction upon oomycete extract exposure. We found that *elt-3(gk121)* mutants were more sensitive to infection (Figure 4E), and this correlated with increased pathogen attachment (Figure 4F). Furthermore, we found a significant decrease in pathogen attachment in both *pals-22(icb89)* mutants and extract-treated animals (Figure 4G), which correlates with the increase in survival observed in infections assays. We conclude that detection of *M. humicola* may lead to anticipatory immunity through changes in the host cuticle that reduce pathogen attachment.

## Induction of *chil-27* in the Epidermis Is Modulated by the Chemosensory Neuron Proteins TAX-2/TAX-4

The main body hypodermis in *C. elegans* is a multinucleate syncytium, so we were surprised to find anterior localization of *chil-27* mRNAs within a single cell (Figure 1C). We argued that this localization may reflect signal transmission from the anterior side of the animal, which is dominated by neurons and glial cells that could readily play a role in pathogen detection. Furthermore,

13 of 43 ORR genes that were not expressed in hypodermis were exclusively expressed in neurons or neuronal support cells (Table S2). We therefore decided to test the possibility that neuronal signaling may regulate detection of *M. humicola* by *C. elegans*.

We focused here on mutations in *tax-2* and *tax-4*, which encode cyclic nucleotide-gated channel α and β subunits responsible for cyclic guanosine monophosphate (cGMP)-activated cation channel activity in a subset of chemosensory neurons (Bretscher et al., 2011; Coburn and Bargmann, 1996). We found that loss-of-function alleles of *tax-2* (*p691*) and *tax-4* (*ks11*, *p678*) led to a strong reduction in the frequency of animals expressing *chil-27p::GFP* upon extract treatment (Figures 5A and 5B), highlighting a potential role for sensory neurons in the response. Failure to detect the pathogen should nullify the protective effect conferred by exposure to extract. We tested this idea by comparing wild-type N2 and *tax-2(p691)* animals in infection assays with and without pretreatment with extract. Although *tax-2(p691)* mutants were found to be more susceptible to infection than N2, they behaved in a similar manner with and without extract treatment (Figure 5C). This correlated with pathogen attachment assays in *tax-2(p691)* mutants, which showed increased attachment compared with N2, but no change upon extract exposure (Figure S4). These results implicate neuronal signaling in the underlying response to oomycete detection.

To narrow down the sensory neurons in which *tax-2*/*tax-4* are required for *M. humicola* detection, we made use of the *tax-2(p694)* allele, where *tax-2* expression is limited to six amphid neurons—AWB, AWC, ASG, ASI, ASK, and ASJ—due to a promoter deletion (Bretscher et al., 2011; Coburn and Bargmann, 1996). Interestingly, *tax-2(p694)* mutants carrying the *chil-27p::GFP* marker responded normally to extract exposure, indicating that expression within one or more of these neurons may be sufficient for the response (Figure 5D). To identify which neuron is involved, we pursued neuron-specific rescue experiments of the impaired *chil-27p::GFP* response to extract. Here, we used a *tax-4* rescue approach to take advantage of the *tax-4* locus that is smaller and thus more convenient for molecular cloning than *tax-2*. We drove *tax-4* expression in AWB, ASK, ASJ, ASI, and ASG/ADL using the *str-1*, *srg-8*, *trx-1*, *gpa-4*, and *gcy-21* promoters, respectively. We found that expressing *tax-4* under an ASK-specific promoter rescued the response of *tax-4* mutants to extract at a greater level than other neurons (Figure 5D). Consistent with this result, genetic ablation of the ASK neurons through expression of a *sra-9p::mCasp-1* transgene reduced the ability of the animals to induce *chil-27p::GFP* as a response to extract treatment (Figure 5E), while ablation of AWC or ASI did not have a similar effect (Figures S5A–S5D). To test whether the ASK neurons are likely to be the primary pathogen-sensing neurons, we monitored their activity upon extract exposure. However, we did not find a real-time calcium response in ASK neurons immediately following extract treatment (Figure S5E). We finally analyzed ASK-ablated animals for pathogen attachment and infection susceptibility, but we did not observe a statistically significant difference compared with wild-type animals (Figures S6F and S6G). Since ASK-ablated neurons do not exhibit complete loss of response to oomycete extract (Figure 5E), this suggests that other sensory neurons are likely to also be involved. Taken together, we conclude that ASK neurons modulate the pathogen-recognition immune response triggered in the epidermis of *C. elegans*.

## DISCUSSION

We extend here our knowledge of host-pathogen interactions focusing on a diverse class of pathogens, the oomycetes, which naturally infect the model organism *C. elegans*. We present evidence that *C. elegans* is able to sense the oomycete *M. humicola* and mount a gene expression response to resist infection. Although *C. elegans* lacks key pattern recognition receptors that are present in other organisms, our work further suggests that the nematode can distinguish between the pathogens it encounters (Irazoqui et al., 2010; Wong et al., 2007). Since *M. humicola* is an obligate pathogen of *C. elegans* and cannot be grown so far in pure culture, we cannot formally rule out the possibility that the active component of the extract is produced by infected animals. However, several observations suggest that the signal is more likely to initiate directly from the pathogen. We have previously shown that mechanical damage of the cuticle by microinjection is unable to induce the *chil-27p::GFP* response (Osman et al., 2018), which is in contrast to the induction of protective antimicrobial peptides in response to cuticular damage upon infection by other epidermal pathogens, such as the fungus *Drechmeria coniospora* (Pujol et al., 2008; Zugasti et al., 2014). Extracts prepared from plates containing animals infected with *D. coniospora* were shown to be unable to induce *chil-27p::GFP* expression and so were extracts prepared from *pals-22(icb89)* mutants that exhibit constitutive *chil* gene expression (Figure S1C). *C. elegans* is known to produce ascarosides under stress, which are sensed through various chemosensory neurons, including the ASK neurons (Kim et al., 2009; Ludewig et al., 2013). However, extracts made from infected *daf-22(m130)* animals, which are defective in ascaroside biosynthesis (Butcher et al., 2009), induce the immune response at a comparable level to extracts made from infected N2 animals (Figure S1C). Taken together, these observations are less compatible with the idea of a host-derived transmissible danger signal that propagates the response in the nematode population. Instead, we suggest that the active component in the extract is more likely to be a PAMP that is sensed by *C. elegans.* Such oomycete-derived molecules may correspond to essential factors of the pathogen's life cycle or infection strategy, for example, cell wall components or secreted virulence factors, as previously described for plant hosts (Fawke et al., 2015).

We uncouple here the response to pathogen recognition (ORR) from responses to tissue damage that occur during infection. The hallmark of the response to *M. humicola* infection is the induction of the *chil* gene family (Osman et al., 2018), which we report here also occurs upon exposure to pathogen extract. The function of *chil* genes has been linked to collagen synthesis and degradation in some systems (Bigg et al., 2006; Iwata et al., 2009), and induction of *chil* genes in *C. elegans* has been proposed to modify properties of the cuticle in a way that reduces pathogen attachment (Osman et al., 2018). Consistent with this model, AFM revealed changes in stiffness upon extract treatment, and extract-treated animals showed reduced pathogen attachment. Although these observations strongly suggest

cuticle changes upon oomycete recognition, whether these changes remain purely at a biochemical level or whether they also have morphological consequences remains unknown. Besides cuticle modification, the induced ORR genes may play a role in other responses conferring a protective effect against oomycete infection. For example, changes in host metabolism are likely to influence immunity, and it is interesting that the ORR list includes a number of metabolic genes. It is conceivable that behavioral changes would represent a putative strategy for nematodes to reduce infection in the wild; however, we did not see such an effect in lab conditions. We hypothesize that ORR genes are more likely to represent effectors of the immune response, as opposed to the machinery required for oomycete detection, which is emphasized by the fact that many ORR genes are only expressed upon exposure to pathogen extract.

Responses of *C. elegans* to different pathogens are thought to be largely distinct. It is therefore interesting that the ORR gene list significantly overlaps with datasets derived from microsporidial or viral infections, which are intracellular pathogens of the intestine (Bakowski et al., 2014). We report here that *pals-22* loss-of-function mutants show induction of more than 50% of the ORR genes, which correlates with their increased resistance to oomycete infection through a reduction in pathogen attachment. It is of note that all shared genes between ORR and IPR are also common between ORR and *pals-22* mutants. Therefore, we argue that the overlap between microsporidial infections and *M. humicola* detection relates to the *pals-22/25* regulatory circuit. Interestingly, *pals-22* and *pals-25* mRNA levels do not change upon extract treatment and infection, and *chil* gene induction upon extract treatment is not compromised in *pals-22* and *pals-22 pals-25* double mutants. This indicates that oomycete detection is unlikely to act through direct changes in the PALS-22/PALS-25 module, although PALS-22/PALS-25 and the extract response pathway may converge on the regulation of a yet unknown downstream factor that triggers *chil* gene expression and cuticle remodeling.

Epidermal immune responses are thought to counteract pathogens that infect through the cuticle. We report here that induction of *chil-27* in the epidermis is partially dependent on the GATA transcription factor ELT-3. This family of transcription factors includes key regulators of pathogen-induced genes and dominates tissue-specific immune responses in *C. elegans* (Block and Shapira, 2015; Shapira et al., 2006; (Yang et al., 2016a)). However, our results support a model wherein the immune response triggered in the epidermis relies on oomycete recognition in neurons. Neuron-to-epidermis communication has been shown to occur during fungal infections and leads to antimicrobial peptide induction in the epidermis (Zugasti and Ewbank, 2009). Our model of neuron-to-epidermis communication culminating in cuticular changes is reminiscent of some recently reported neural regulation of the cuticle barrier, through the neuropeptide Y receptor NPR-8, in response to infection by bacterial pathogens (Sellegounder et al., 2019). NPR-8 is not directly involved in sensing bacteria and acts to suppress cuticular collagen genes that contribute to host defense (Sellegounder et al., 2019). Interestingly, neuron-to-epidermis regulation in the oomycete context is linked to pathogen recognition and does not appear to involve regulation of the same collagen genes, which are not part of the identified ORR. Therefore, neuronal regulation of cuticle remodeling may provide a broad mechanism for protection against pathogens infecting through the epidermis and also those that colonize the *C. elegans* intestine.

Our results are consistent with reports of various sensory neurons modulating innate immune pathways, physical barrier defense, and behavior in *C. elegans* (Cao et al., 2017b; Foster et al., 2020; Styer et al., 2008). TAX-2/4-dependent signaling in chemosensory neurons has been previously linked to cuticle morphology and epidermal pathogen avoidance (Bretscher et al., 2011; Coburn and Bargmann, 1996; Sellegounder et al., 2019; Yook and Hodgkin, 2007). Tissue-specific rescue and neuronal ablation experiments revealed ASK neurons to be the most prominent in modulating the response to oomycete recognition. While ASK neurons have been previously associated with avoidance of certain chemical repellents (Hilliard et al., 2002), an involvement in modulation of pathogen detection has not been reported. Given that ASK-ablated animals are still able to respond to pathogen extract, and restoration of *tax-4* function in other sensory neurons does partially rescue *chil-27p::GFP* induction in *tax-4(ks11)* mutant animals upon extract treatment, we anticipate that other neurons also contribute to this response. The lack of a detectable calcium response in ASK further suggests that these may not be the primary oomycete-sensing neurons, although they are involved in modulating the mounting of the immune response in the hypodermis. A similar scenario has been reported in another study where AWB, AWC, and ASJ neurons regulate cuticle dynamics in response to bacterial infection in *C. elegans*, but none of these neurons are actually involved in sensing the pathogenic bacteria (Sellegounder et al., 2019). Future work will focus on dissecting the machinery and specificity of neuronal and intercellular signaling in the context of oomycete recognition. We speculate that such cross-tissue communication may be an important feature of animal defense to oomycete infection.

## STAR★METHODS

Detailed methods are provided in the online version of this paper and include the following:

- KEY RESOURCES TABLE
- RESOURCE AVAILABILITY
  - Lead Contact
  - Materials Availability
  - Data and Code Availability
- EXPERIMENTAL MODEL AND SUBJECT DETAILS
- METHOD DETAILS
  - Pathogen extract preparation
  - Phenotypic assays
  - qRT-PCR
  - RNA-seq
  - GSEA
  - Molecular cloning and transgenesis
  - Microscopy
- QUANTIFICATION AND STATISTICAL ANALYSIS

## SUPPLEMENTAL INFORMATION

Supplemental Information can be found online at https://doi.org/10.1016/j.celrep.2020.108604.

## ACKNOWLEDGMENTS

We thank Christina Vagena-Pantoula for technical help and Rachel McMullan for the pRJM163 plasmid. We thank Mark Hintze, Vladimir Lazetic, and Emily Troemel for comments on the manuscript and Mandayam Srinivasan for access to the AFM equipment. Some *C. elegans* strains were provided by the CGC, which is funded by NIH Office of Research Infrastructure Programs (P40 OD010440). We acknowledge the support from the Wellcome Trust (219448/Z/19/Z). F.D. is the recipient of an Imperial College Schrödinger PhD Scholarship.

## AUTHOR CONTRIBUTIONS

M.K.F. and M.G. carried out the majority of the experiments. M.K.F. performed the RNA-seq analysis. F.D. performed the molecular cloning and induction assays. C.L.E. performed the AFM experiment. E.K. and W.R.S. contributed the calcium imaging data. M.B. designed and supervised the work. All authors contributed to the writing of the manuscript.

## DECLARATION OF INTERESTS

The authors declare no competing interests.

Received: July 7, 2020
Revised: November 2, 2020
Accepted: December 14, 2020
Published: January 12, 2021

## REFERENCES

Akira, S., Uematsu, S., and Takeuchi, O. (2006). Pathogen recognition and innate immunity. Cell *124*, 783–801.

Angeles-Albores, D., N Lee, R.Y., Chan, J., and Sternberg, P.W. (2016). Tissue enrichment analysis for *C. elegans* genomics. BMC Bioinformatics *17*, 366.

Bakowski, M.A., Desjardins, C.A., Smelkinson, M.G., Dunbar, T.L., Lopez-Moyado, I.F., Rifkin, S.A., Cuomo, C.A., and Troemel, E.R. (2014). Ubiquitin-mediated response to microsporidia and virus infection in *C. elegans*. PLoS Pathog. *10*, e1004200.

Beakes, G.W., Glockling, S.L., and Sekimoto, S. (2012). The evolutionary phylogeny of the oomycete "fungi". Protoplasma *249*, 3–19.

Bigg, H.F., Wait, R., Rowan, A.D., and Cawston, T.E. (2006). The mammalian chitinase-like lectin, YKL-40, binds specifically to type I collagen and modulates the rate of type I collagen fibril formation. J. Biol. Chem. *281*, 21082–21095.

Block, D.H., and Shapira, M. (2015). GATA transcription factors as tissue-specific master regulators for induced responses. Worm *4*, e1118607.

Bray, N.L., Pimentel, H., Melsted, P., and Pachter, L. (2016). Near-optimal probabilistic RNA-seq quantification. Nat. Biotechnol. *34*, 525–527.

Bretscher, A.J., Kodama-Namba, E., Busch, K.E., Murphy, R.J., Soltesz, Z., Laurent, P., and de Bono, M. (2011). Temperature, oxygen, and salt-sensing neurons in *C. elegans* are carbon dioxide sensors that control avoidance behavior. Neuron *69*, 1099–1113.

Butcher, R.A., Ragains, J.R., Li, W., Ruvkun, G., Clardy, J., and Mak, H.Y. (2009). Biosynthesis of the Caenorhabditis elegans dauer pheromone. Proc. Natl. Acad. Sci. USA *106*, 1875–1879.

Cao, J., Packer, J.S., Ramani, V., Cusanovich, D.A., Huynh, C., Daza, R., Qiu, X., Lee, C., Furlan, S.N., Steemers, F.J., et al. (2017a). Comprehensive single-cell transcriptional profiling of a multicellular organism. Science *357*, 661–667.

Cao, X., Kajino-Sakamoto, R., Doss, A., and Aballay, A. (2017b). Distinct roles of sensory neurons in mediating pathogen avoidance and neuropeptide-dependent immune regulation. Cell Rep. *21*, 1442–1451.

Chew, Y.L., Tanizawa, Y., Cho, Y., Zhao, B., Yu, A.J., Ardiel, E.L., Rabinowitch, I., Bai, J., Rankin, C.H., Lu, H., et al. (2018). An Afferent Neuropeptide System Transmits Mechanosensory Signals Triggering Sensitization and Arousal in *C. elegans*. Neuron *99*, 1233–1246.e6.

Cho, Y., Porto, D.A., Hwang, H., Grundy, L.J., Schafer, W.R., and Lu, H. (2017). Automated and controlled mechanical stimulation and functional imaging in vivo in C. elegans. Lab Chip *17*, 2609–2618.

Cho, Y., Oakland, D.N., Lee, S.A., Schafer, W.R., and Lu, H. (2018). On-chip functional neuroimaging with mechanical stimulation in Caenorhabditis elegans larvae for studying development and neural circuits. Lab Chip *18*, 601–609.

Coburn, C.M., and Bargmann, C.I. (1996). A putative cyclic nucleotide-gated channel is required for sensory development and function in *C. elegans*. Neuron *17*, 695–706.

De Cock, A.W., Mendoza, L., Padhye, A.A., Ajello, L., and Kaufman, L. (1987). *Pythium insidiosum sp. nov*., the etiologic agent of pythiosis. J. Clin. Microbiol. *25*, 344–349.

Ermolaeva, M.A., and Schumacher, B. (2014). Insights from the worm: the *C. elegans* model for innate immunity. Semin. Immunol. *26*, 303–309.

Ewbank, J.J., and Pujol, N. (2016). Local and long-range activation of innate immunity by infection and damage in *C. elegans*. Curr. Opin. Immunol. *38*, 1–7.

Fawke, S., Doumane, M., and Schornack, S. (2015). Oomycete interactions with plants: infection strategies and resistance principles. Microbiol. Mol. Biol. Rev. *79*, 263–280.

Foster, K.J., Cheesman, H.K., Liu, P., Peterson, N.D., Anderson, S.M., and Pukkila-Worley, R. (2020). Innate immunity in the *C. elegans* intestine Is programmed by a neuronal regulator of AWC olfactory neuron development. Cell Rep. *31*, 107478.

Fry, W. (2008). *Phytophthora infestans*: the plant (and R gene) destroyer. Mol. Plant Pathol. *9*, 385–402.

Ge, S.X., Jung, D., and Yao, R. (2019). ShinyGO: A graphical gene-set enrichment tool for animals and plants. Bioinformatics *36*, 2628–2629.

Hilliard, M.A., Bargmann, C.I., and Bazzicalupo, P. (2002). *C. elegans* responds to chemical repellents by integrating sensory inputs from the head and the tail. Curr. Biol. *12*, 730–734.

Hintze, M., Koneru, S.L., Gilbert, S.P.R., Katsanos, D., Lambert, J., and Barkoulas, M. (2020). A cell fate switch in the *Caenorhabditis elegans* seam cell lineage occurs through modulation of the Wnt asymmetry pathway in response to temperature increase. Genetics *214*, 927–939.

Hou, S., Liu, Z., Shen, H., and Wu, D. (2019). Damage-associated molecular pattern-triggered immunity in plants. Front. Plant Sci. *10*, 646.

Irazoqui, J.E., Troemel, E.R., Feinbaum, R.L., Luhachack, L.G., Cezairliyan, B.O., and Ausubel, F.M. (2010). Distinct pathogenesis and host responses during infection of *C. elegans* by *P. aeruginosa* and *S. aureus*. PLoS Pathog. *6*, e1000982.

Iwata, T., Kuwajima, M., Sukeno, A., Ishimaru, N., Hayashi, Y., Wabitsch, M., Mizusawa, N., Itakura, M., and Yoshimoto, K. (2009). YKL-40 secreted from adipose tissue inhibits degradation of type I collagen. Biochem. Biophys. Res. Commun. *388*, 511–516.

Katsanos, D., Koneru, S.L., Mestek Boukhibar, L., Gritti, N., Ghose, R., Appleford, P.J., Doitsidou, M., Woollard, A., van Zon, J.S., Poole, R.J., and Barkoulas, M. (2017). Stochastic loss and gain of symmetric divisions in the *C. elegans* epidermis perturbs robustness of stem cell number. PLoS Biol. *15*, e2002429.

Kim, D.H., and Ewbank, J.J. (2018). Signaling in the innate immune response. WormBook, 1–51.

Kim, K., Sato, K., Shibuya, M., Zeiger, D.M., Butcher, R.A., Ragains, J.R., Clardy, J., Touhara, K., and Sengupta, P. (2009). Two chemoreceptors mediate developmental effects of dauer pheromone in *C. elegans*. Science *326*, 994–998.

Kim, W., Underwood, R.S., Greenwald, I., and Shaye, D.D. (2018). OrthoList 2: A new comparative genomic analysis of human and *Caenorhabditis elegans* genes. Genetics *210*, 445–461.

Krajaejun, T., Sathapatayavongs, B., Pracharktam, R., Nitiyanant, P., Leelachaikul, P., Wanachiwanawin, W., Chaiprasert, A., Assanasen, P., Saipetch, M., Mootsikapun, P., et al. (2006). Clinical and epidemiological analyses of human pythiosis in Thailand. Clin. Infect. Dis. *43*, 569–576.

Ludewig, A.H., Izrayelit, Y., Park, D., Malik, R.U., Zimmermann, A., Mahanti, P., Fox, B.W., Bethke, A., Doering, F., Riddle, D.L., and Schroeder, F.C. (2013). Pheromone sensing regulates *Caenorhabditis elegans* lifespan and stress resistance via the deacetylase SIR-2.1. Proc. Natl. Acad. Sci. USA *110*, 5522–5527.

Meisel, J.D., Panda, O., Mahanti, P., Schroeder, F.C., and Kim, D.H. (2014). Chemosensation of bacterial secondary metabolites modulates neuroendocrine signaling and behavior of *C. elegans*. Cell *159*, 267–280.

Mendoza, L., and Newton, J.C. (2005). Immunology and immunotherapy of the infections caused by *Pythium insidiosum*. Med. Mycol. *43*, 477–486.

Mogensen, T.H. (2009). Pathogen recognition and inflammatory signaling in innate immune defenses. Clin. Microbiol. Rev. *22*, 240–273.

Mootha, V.K., Lindgren, C.M., Eriksson, K.F., Subramanian, A., Sihag, S., Lehar, J., Puigserver, P., Carlsson, E., Ridderstråle, M., Laurila, E., et al. (2003). PGC-1alpha-responsive genes involved in oxidative phosphorylation are coordinately downregulated in human diabetes. Nat. Genet. *34*, 267–273.

Osman, G.A., Fasseas, M.K., Koneru, S.L., Essmann, C.L., Kyrou, K., Srinivasan, M.A., Zhang, G., Sarkies, P., Félix, M.A., and Barkoulas, M. (2018). Natural Infection of *C. elegans* by an Oomycete Reveals a New Pathogen-Specific Immune Response. Curr. Biol. *28*, 640–648.e5.

Pfaffl, M.W. (2001). A new mathematical model for relative quantification in real-time RT-PCR. Nucleic Acids Res. *29*, e45.

Pimentel, H., Bray, N.L., Puente, S., Melsted, P., and Pachter, L. (2017). Differential analysis of RNA-seq incorporating quantification uncertainty. Nat. Methods *14*, 687–690.

Pradel, E., Zhang, Y., Pujol, N., Matsuyama, T., Bargmann, C.I., and Ewbank, J.J. (2007). Detection and avoidance of a natural product from the pathogenic bacterium *Serratia marcescens* by *Caenorhabditis elegans*. Proc. Natl. Acad. Sci. USA *104*, 2295–2300.

Pujol, N., Cypowyj, S., Ziegler, K., Millet, A., Astrain, A., Goncharov, A., Jin, Y., Chisholm, A.D., and Ewbank, J.J. (2008). Distinct innate immune responses to infection and wounding in the *C. elegans* epidermis. Curr. Biol. *18*, 481–489.

Pukkila-Worley, R., Ausubel, F.M., and Mylonakis, E. (2011). *Candida albicans* infection of *Caenorhabditis elegans* induces antifungal immune defenses. PLoS Pathog. *7*, e1002074.

Raudvere, U., Kolberg, L., Kuzmin, I., Arak, T., Adler, P., Peterson, H., and Vilo, J. (2019). g:Profiler: a web server for functional enrichment analysis and conversions of gene lists (2019 update). Nucleic Acids Res. *47* (*W1*), W191–W198.

Reddy, K.C., Dror, T., Sowa, J.N., Panek, J., Chen, K., Lim, E.S., Wang, D., and Troemel, E.R. (2017). An intracellular pathogen response pathway promotes proteostasis in *C. elegans*. Curr. Biol. *27*, 3544–3553.e5.

Reddy, K.C., Dror, T., Underwood, R.S., Osman, G.A., Elder, C.R., Desjardins, C.A., Cuomo, C.A., Barkoulas, M., and Troemel, E.R. (2019). Antagonistic paralogs control a switch between growth and pathogen resistance in *C. elegans*. PLoS Pathog. *15*, e1007528.

Schulenburg, H., and Félix, M.A. (2017). The natural biotic environment of *Caenorhabditis elegans*. Genetics *206*, 55–86.

Sellegounder, D., Liu, Y., Wibisono, P., Chen, C.H., Leap, D., and Sun, J. (2019). Neuronal GPCR NPR-8 regulates *C. elegans* defense against pathogen infection. Sci. Adv. *5*, eaaw4717.

Shapira, M., Hamlin, B.J., Rong, J., Chen, K., Ronen, M., and Tan, M.W. (2006). A conserved role for a GATA transcription factor in regulating epithelial innate immune responses. Proc. Natl. Acad. Sci. USA *103*, 14086–14091.

Sifri, C.D., Begun, J., Ausubel, F.M., and Calderwood, S.B. (2003). *Caenorhabditis elegans* as a model host for *Staphylococcus aureus* pathogenesis. Infect. Immun. *71*, 2208–2217.

Singh, J., and Aballay, A. (2020). Neural control of behavioral and molecular defenses in *C. elegans*. Curr. Opin. Neurobiol. *62*, 34–40.

Stiernagle, T. (2006). Maintenance of C. elegans. WormBook, 1–11.

Styer, K.L., Singh, V., Macosko, E., Steele, S.E., Bargmann, C.I., and Aballay, A. (2008). Innate immunity in *Caenorhabditis elegans* is regulated by neurons expressing NPR-1/GPCR. Science *322*, 460–464.

Subramanian, A., Tamayo, P., Mootha, V.K., Mukherjee, S., Ebert, B.L., Gillette, M.A., Paulovich, A., Pomeroy, S.L., Golub, T.R., Lander, E.S., and Mesirov, J.P. (2005). Gene set enrichment analysis: a knowledge-based approach for interpreting genome-wide expression profiles. Proc. Natl. Acad. Sci. USA *102*, 15545–15550.

Tan, M.W., Mahajan-Miklos, S., and Ausubel, F.M. (1999). Killing of *Caenorhabditis elegans* by *Pseudomonas aeruginosa* used to model mammalian bacterial pathogenesis. Proc. Natl. Acad. Sci. USA *96*, 715–720.

Twumasi-Boateng, K., and Shapira, M. (2012). Dissociation of immune responses from pathogen colonization supports pattern recognition in *C. elegans*. PLoS ONE *7*, e35400.

Wong, D., Bazopoulou, D., Pujol, N., Tavernarakis, N., and Ewbank, J.J. (2007). Genome-wide investigation reveals pathogen-specific and shared signatures in the response of *Caenorhabditis elegans* to infection. Genome Biol. *8*, R194.

Yang, W., Dierking, K., Rosenstiel, P.C., and Schulenburg, H. (2016a). GATA transcription factor as a likely key regulator of the *Caenorhabditis elegans* innate immune response against gut pathogens. Zoology (Jena) *119*, 244–253.

Yang, W., Dierking, K., and Schulenburg, H. (2016b). WormExp: a web-based application for a *Caenorhabditis elegans*-specific gene expression enrichment analysis. Bioinformatics *32*, 943–945.

Yook, K., and Hodgkin, J. (2007). Mos1 mutagenesis reveals a diversity of mechanisms affecting response of *Caenorhabditis elegans* to the bacterial pathogen *Microbacterium nematophilum*. Genetics *175*, 681–697.

Zugasti, O., and Ewbank, J.J. (2009). Neuroimmune regulation of antimicrobial peptide expression by a noncanonical TGF-beta signaling pathway in *Caenorhabditis elegans* epidermis. Nat. Immunol. *10*, 249–256.

Zugasti, O., Bose, N., Squiban, B., Belougne, J., Kurz, C.L., Schroeder, F.C., Pujol, N., and Ewbank, J.J. (2014). Activation of a G protein-coupled receptor by its endogenous ligand triggers the innate immune response of *Caenorhabditis elegans*. Nat. Immunol. *15*, 833–838.

# STAR★METHODS

## KEY RESOURCES TABLE

| REAGENT or RESOURCE | SOURCE | IDENTIFIER |
|---|---|---|
| Bacterial and Virus Strains | | |
| *E. coli*: OP50 | Caenorhabditis Genetics Center | Wormbase ID: OP50 |
| Ahringer RNAi Libraries in *E. coli* HT115 | Source Bioscience | N/A |
| Chemicals, Peptides, and Recombinant Proteins | | |
| Calcofluor White | Sigma | Cat: 910090 |
| IPTG | Sigma | Cat: I6758 |
| DNase I | Promega | Cat: Z358A-C |
| TRIzol | Invitrogen | Cat: 15596026 |
| SuperScript IV Reverse Transcriptase | Invitrogen | Cat: 18090010 |
| Critical Commercial Assays | | |
| LightCycler480 SYBR Green I Master | Roche | Cat: 4707516001 |
| Deposited Data | | |
| RNA-seq data, normalized per gene sequence counts and differentially expressed genes as determined by Kallisto & Sleuth respectively | This paper | GEO: GSE150135 |
| Experimental Models: Organisms/Strains | | |
| *M. humicola* Lisbon isolate | Osman et al., 2018 | JUo1 |
| *C. elegans: wild-type* N2 | Caenorhabditis Genetics Center | Wormbase ID: N2 |
| *C. elegans: unc-119(ed3) III; icbSi3[dpy-7::GFP::H2B::unc-54 3′UTR+cb-unc-119]* | This paper | MBA260 |
| *C. elegans: icbIs4[chil-27p::GFP, col-12p::mCherry-pest] II* | Osman et al., 2018 | MBA281 |
| *C. elegans: icbIs5[chil-27p::GFP, col-12p::mCherry-pest] IV* | Osman et al., 2018 | MBA282 |
| *C. elegans: pals-22(icb89)III* | This paper | MBA1080 |
| *C. elegans: elt-3(gk121); icbIs4[chil-27p::GFP, col-12p::mCherry-pest] II* | This paper | MBA397 |
| *C. elegans: pals-22(icb90) pals-25(icb92) III; icbIs4[chil-27p::GFP, col-12p::mCherry-pest] II* | Reddy et al., 2019 | MBA791 |
| *C. elegans: tax-2(p691) I; icbIs4[chil-27p::GFP, col-12p::mCherry-pest] II* | This paper | MBA507 |
| *C. elegans: tax-2(p694) I; icbIs4[chil-27p::GFP, col-12p::mCherry-pest] II* | This paper | MBA667 |
| *C. elegans: tax-4(ks11) III; icbIs5[chil-27p::GFP, col-12p::mCherry-pest] IV* | This paper | MBA668 |
| *C. elegans: tax-2(p691) I* | Caenorhabditis Genetics Center | Wormbase ID: PR691 |
| *C. elegans: icbIs5[chil-27p::GFP, col-12p::mCherry-pest] IV; tax-4(ks11) III.; icbEx246[pFD18(tax-4::tax-4::wrmScarlet::SL2::unc-54),pRJM163 (bus-1::GFP), BJ36]* | This paper | MBA1069 |
| *C. elegans: icbIs5[chil-27p::GFP, col-12p::mCherry-pest] IV; tax-4(ks11) III.; icbEx247[pFD19(str-1::tax-4::wrmScarlet::SL2::unc-54),pRJM163 (bus-1::GFP), BJ36]* | This paper | MBA1070 |
| *C. elegans: icbIs5[chil-27p::GFP, col-12p::mCherry-pest] IV; tax-4(ks11) III.; icbEx243[pFD8(srg-8::tax-4::wrmScarlet::SL2::unc-54),pRJM163 (bus-1::GFP), BJ36]* | This paper | MBA1066 |
| *C. elegans: icbIs5[chil-27p::GFP, col-12p::mCherry-pest] IV; tax-4(ks11) III.; icbEx242[pFD7(trx-1::tax-4::wrmScarlet::SL2::unc-54),pRJM163 (bus-1::GFP), BJ36]* | This paper | MBA1065 |

(*Continued on next page*)

*Continued*

| REAGENT or RESOURCE | SOURCE | IDENTIFIER |
|---|---|---|
| *C. elegans: icbIs5[chil-27p::GFP, col-12p::mCherry-pest] IV; tax-4(ks11) III.; icbEx244[pFD9(gpa-4::tax-4::wrmScarlet::SL2::unc-54),pRJM163 (bus-1::GFP), BJ36]* | This paper | MBA1067 |
| *C. elegans: icbIs5[chil-27p::GFP, col-12p::mCherry-pest] IV; tax-4(ks11) III.; icbEx245[pFD11(gcy-21::tax-4::wrmScarlet::SL2::unc-54),pRJM163 (bus-1::GFP), BJ36]* | This paper | MBA1068 |
| *C. elegans: icbIs4[pGO4, pCMH1195] II; qrIs2[sra-9p::mCasp-1, elt-2p::GFP]* | This paper | MBA1081 |
| *C. elegans: oyIs85 [ceh-36p::TU#813 + ceh-36p::TU#814 + srtx-1p::GFP + unc-122p::DsRed];icbIs5[chil-27p::GFP, col-12p::mCherry-pest] IV.* | This paper | MBA665 |
| *C. elegans: oyIs84 [gpa-4p::TU#813 + gcy-27p::TU#814 + gcy-27p::GFP + unc-122p::DsRed]; icbIs5[chil-27p::GFP, col-12p::mCherry-pest] IV.* | This paper | MBA666 |
| *C. elegans: daf-22(m130) II* | Caenorhabditis Genetics Center | Wormbase ID:DR476 |
| *C. elegans: elt-3(gk121) X* | Caenorhabditis Genetics Center | Wormbase ID:VC143 |
| *C. elegans: qrIs2[sra-9p::mCasp-1, elt-2p::GFP]* | Caenorhabditis Genetics Center | Wormbase ID:PS6025 |
| *C. elegans: ljEx1186[sra-9p::GCaMP3::SL2-tagRFP (pWRS1483); unc-122p::RFP]* | This paper | AQ4259 |
| Oligonucleotides | | |
| oligos, see Table S6 | Eurofins Genomics | N/A |
| smFISH probe, see Table S6 | Biomers.net | N/A |
| Recombinant DNA | | |
| *trx-1p::tax-4:::wrmScarlet::SL2::unc-54* | This paper | pFD7 |
| *srg-8p::tax-4::wrmScarlet::SL2::unc-54* | This paper | pFD8 |
| *gpa-4p::tax-4::wrmScarlet::SL2::unc-54* | This paper | pFD9 |
| *gcy-21p::tax-4::wrmScarlet::SL2::unc-54* | This paper | pFD11 |
| *str-1p::tax-4::wrmScarlet::SL2::unc-54* | This paper | pFD19 |
| *tax-4p::tax-4::wrmScarlet::SL2::unc-54* | This paper | pFD18 |
| Software and Algorithms | | |
| GraphPad Prism 7 | GraphPad Software, La Jolla, CA | https://www.graphpad.com/scientific-software/prism/ |
| ImageJ | NIH Image | https://imagej.nih.gov/ij/ |
| LightCycler480 (version 1.5.1.62) | Roche | N/A |
| Kallisto | Bray et al., 2016 | https://pachterlab.github.io/kallisto/ |
| Gene Set Enrichment Analysis (GSEA) software v4.0.3 | Mootha et al., 2003<br>Subramanian et al., 2005 | https://www.gsea-msigdb.org/gsea/index.jsp |

## RESOURCE AVAILABILITY

### Lead Contact

Further information and requests for resources and reagents should be directed to and will be fulfilled by the Lead Contact, Michalis Barkoulas (m.barkoulas@imperial.ac.uk).

### Materials Availability

All unique/stable reagents generated in this study are available from the Lead Contact without restriction.

### Data and Code Availability

The raw RNA-seq data have been deposited to NCBI GEO under accession GEO: GSE150135.

## EXPERIMENTAL MODEL AND SUBJECT DETAILS

*C. elegans* strains were cultured on NGM plates seeded with *E. coli* OP50 at 20°C under standard conditions (Stiernagle, 2006). *M. humicola* was grown and maintained at 25°C on NGM plates along with *C. elegans* N2. The oomycete cultures can be maintained indefinitely by chunking onto fresh NGM plates with OP50. For use in experiments, freshly killed animals that are full of sporangia were washed off plates that were chunked 2-3 days earlier. Infected animals were separated from live animals using 70 μm nylon filter mesh immersed in M9 buffer. This allowed for live animals to swim through the mesh while the dead animals were retained. The dead animals were gently disrupted using a grinder in a microcentrifuge tube and the numbers of sporangia were counted under a microscope. Dilutions of these suspensions were made using M9 and they were added to standard NGM plates with OP50 to be used in infection or attachment assays. A list of strains used in this study is presented in the Key Resources Table.

## METHOD DETAILS

### Pathogen extract preparation

Crude pathogen extract was prepared by growing *M. humicola* strain JUo1 on 90mm standard or egg yolk plates with N2 until starvation. Plates were washed with 5 mL $H_2O$ each and the suspension was centrifuged in 50 mL tubes for 20 minutes at 5000 rpm to remove debris. The supernatant was collected, filter-sterilized using 0.2 μm filters and autoclaved. The extract was stored for long term at −80°C until use. The control extract was also prepared in the same way from N2 animals grown in the absence of any pathogen.

### Phenotypic assays

All infection, attachment and lifespan assays were performed at 20°C in triplicates on standard NGM plates with OP50. For populations treated with extract, this was added at the L2 stage. Infection assays were started by moving 30 L4 animals to plates containing *M. humicola* (as described above) and experiments were done in triplicates, so 90 animals were used per condition in total. The animals were scored for visible infection (i.e., development of sporangia) and the live ones were moved to new plates containing *M. humicola* for a period up to 7 days, depending on how the infection progressed. Dead animals without signs of infection or lost animals were censored. N2 animals were bleached 8h after *pals-22(-)* mutants in order to have synchronized development as the latter exhibit developmental delay. For lifespan assays, the same conditions were used without the presence of oomycete and the assay continued until all animals had died, while lost or bagged animals were censored. All experiments were repeated at least three times (Table S5) and GraphPad Prism (GraphPad Software Inc.) was used to plot and compare survival curves. The log-rank test was used to assess statistical significance and a $p$ value of < 0.05 was considered significant.

For the attachment assays, day 1 adult animals were moved to plates containing *M. humicola* at approximately 100 animals per plate. They were left for 4h at 20°C and then washed with M9 buffer. Attachment was scored by two methods, using FISH as previously described (Osman et al., 2018) and imaged using an epifluorescence Ti-eclipse (Nikon) microscope equipped with a low noise CCD camera (Andor iKon-M934), or by staining briefly in calcofluor white (Sigma) and visualized with a DAPI filter. Images were analyzed using ImageJ. A chi-square test was used to test significance of the results.

For induction assays, serial dilutions of the extract were made in water and 100 μL of each was added to OP50 plates. The extract was added on top of the bacterial lawn and the plate was moved gently to allow it to spread uniformly. *C. elegans* eggs suspended in M9 were added to each plate to have 100-150 eggs in total. The plates were incubated at 20°C and scored for *chil-27p::GFP* induction after 48h using Zeiss Axio Zoom V16 (Zeiss) microscope. Animals were classified in two groups (GFP positive and negative) and *col-12p::mCherry* expression was confirmed in both cases. For RNAi experiment, 6-8 L4 stage worms were added onto respective RNAi plates containing extract and *chil-27p::GFP* induction was scored after 72h when the progeny on the plate were predominantly L4 stage or beyond. All RNAi clones were obtained from the Ahringer collection (Source BioScience) and were verified by sequencing. The expression of dsRNA was induced by the addition of 1 mM isopropyl β-D-1-thiogalactopyranoside (IPTG; Sigma).

For the avoidance assay, 100 L4 stage animals suspended in M9 were spotted in the center of an OP50 plate with and without extract; and animals present within the lawn were counted after 2h and 24h.

### qRT-PCR

*C. elegans* at L4 stage were treated with extract for 4 h and total RNA was extracted from animals grown at the appropriate stage/exposure time using the TRIzol reagent (Invitrogen) and isopropanol/ethanol precipitation. NanoDrop quantification (Thermo Scientific) and gel electrophoresis was used to assess the quantity and quality of RNA. 2 μg of RNA from all samples was subjected to DNase (Promega) treatment and cDNA was synthesized using Superscript IV (Invitrogen) with Oligo(dT) primers as per manufacturer's instructions. Real-time PCR was performed using LightCycler480 SYBR Green I Master (Roche) in a LightCycler480 instrument and Ct values were derived using the LightCycler480 software and second derivative maximum method. Expression levels of *chil-27* were normalized with the values obtained for the reference gene *pmp-3.* The efficiency of each set of primers and calculation of levels of induction was determined as described previously (Pfaffl, 2001). Experiments were performed in biological triplicates and oligos used are listed in the Key Resources Table.

### RNA-seq

*C. elegans* populations were synchronized by bleaching and grown until the L4 stage. For all extract treatments, 300 μL of undiluted extract was added to NGM plates. Infection samples were exposed to conditions as described for the infection assays. RNA was extracted in triplicates at 1h, 4h, 12h and 24h for extract samples and 12h, 24h and 48h post exposure to the pathogen. RNA samples were sequenced by BGI Genomics (Hong Kong). Pseudoalignment was performed using Kallisto (Bray et al., 2016) and the WS235 transcriptome from Wormbase. Analysis of counts was performed using Sleuth and a Wald test for two-sample comparisons (Pimentel et al., 2017).

### GSEA

The RNA-seq datasets from exposure to extract at 1h and 4h were compared to other *C. elegans* transcriptomic datasets using Gene Set Enrichment Analysis (GSEA) software v4.0.3 (Mootha et al., 2003; Subramanian et al., 2005). The genes that were significantly differentially expressed upon infection (FDR < 0.1) were ranked based on their b value in descending order. We used 65 gene sets for the analysis derived from (Osman et al., 2018), (Reddy et al., 2019) and WormExp ((Yang et al., 2016b), which can be found in Table S3). Pre-ranked analysis with weighted enrichment statistic, 1000 permutations and a minimum of 15 genes overlap, was performed independently for extract treatment at 1h and 4h time points. The NES-values of gene sets with FDR < 0.25 and nominal p value < 0.05 were considered as significant and the results are summarized in Table S3.

### Molecular cloning and transgenesis

To generate constructs for neuron-specific rescue of *tax-4* mutants, splice leader 2 (SL2) sequence was amplified from genomic DNA using primers SL2-F and SL2-R. Restriction sites and Gibson Assembly complementarity arms were added to this SL2 amplicon using primers SL2-F Gib and SL2-R. WrmScarlet followed up by *unc-54* 3′UTR was amplified from the plasmid pDK38C2 using primers SL2 scarlet-F and BJ36-unc54-ter-R. Fragments were cloned using a Gibson Assembly protocol into SpeI-digested BJ97 to give the intermediate vector pFD1. Promoter regions and *tax-4* coding sequence were amplified from genomic DNA and cloned into AvrII-digested pFD1 using Gibson Assembly. Constructs were injected into the *tax-4(ks11)* strain at a concentration of 5ng/μl along with 20ng/μl of *bus-1p::GFP* (pRJM163) as a co-injection marker. A list of oligos used in this study is presented in the Key Resources Table.

### Microscopy

Single molecule FISH was performed as previously described (Hintze et al., 2020; Katsanos et al., 2017). Briefly, animals were synchronized by bleaching and treated with extract for 1 hour at mid-L2 stage. Animals were fixed with 4% formaldehyde (Sigma-Aldrich) in 1x PBS (Ambion) for 45 min and were permeabilised with 70% Ethanol for 24 hours. Hybridization was performed at 30°C for 16 hours. A list of oligos included in the *chil-27* probe can be found in the Key Resources Table. Imaging was performed in an inverted and fully motorised epifluorescence microscope (Nikon Ti-eclipse) with an iKon M DU-934 CCD camera (Andor) controlled via the NIS-Elements software (Nikon) using the 100x objective. Atomic force microscopy was performed as previously described (Osman et al., 2018) using day 1 N2 adults grown on plates supplemented with oomycete or N2 control extract. Calcium imaging was performed on day 2 adult animals in a custom-designed microfluidic devices as previously described (Chew et al., 2018; Cho et al., 2018; Cho et al., 2017). Briefly, experiments were performed on a Leica Axiovert 135 inverted microscope using a 40x air objective. Video sequences were captured using a Hamamatsu ORCA-R2 (C10600-10B) camera with 100 msec exposure time. Simultaneous dual color imaging was performed using an OptoSplit II (Andor Technology) beamsplitter containing a GFP (520 nm)/RFP(605 nm) filter set. CoolLED's pE-300 white was used as a light source for fluorescent imaging. Imaging was carried out in S-basal buffer (100mM NaCl, 0.05M phosphate buffer pH 6.0, 5 μg/mL cholesterol). Stimulus was 1/10 diluted pathogen extract in S-basal as a 10 s pulse and was delivered at t = 5 s after recordings were started. Videos were recorded for 25 s following stimulus delivery. For analysis of calcium transients, fluorescence intensities for each frame were extracted using a custom MATLAB script (Cho et al., 2018; Cho et al., 2017).

## QUANTIFICATION AND STATISTICAL ANALYSIS

Graphic representation and statistical analysis were performed using the GraphPad Prism 7 software. Data shown in bar graphs indicate mean, and error bars represent standard error of the mean or standard error of the proportion as indicated. A log-rank, chi-square, Fisher's exact test, ANOVA and Tukey's multiple comparison test was used to analyze data as described in the figure legends. Results were considered statistically significant when $p < 0.05$. Asterisks in figures indicate corresponding statistical significance as it follows: * $p < 0.05$; ** $p < 0.01$; *** $p < 0.001$; **** $p < 0.0001$. Overlaps between gene lists were assessed based on a hypergeometric test (nemates.org).

Cell Reports, Volume *34*

## Supplemental Information

# Chemosensory Neurons Modulate the Response

# to Oomycete Recognition in *Caenorhabditis elegans*

**Michael K. Fasseas, Manish Grover, Florence Drury, Clara L. Essmann, Eva Kaulich, William R. Schafer, and Michalis Barkoulas**

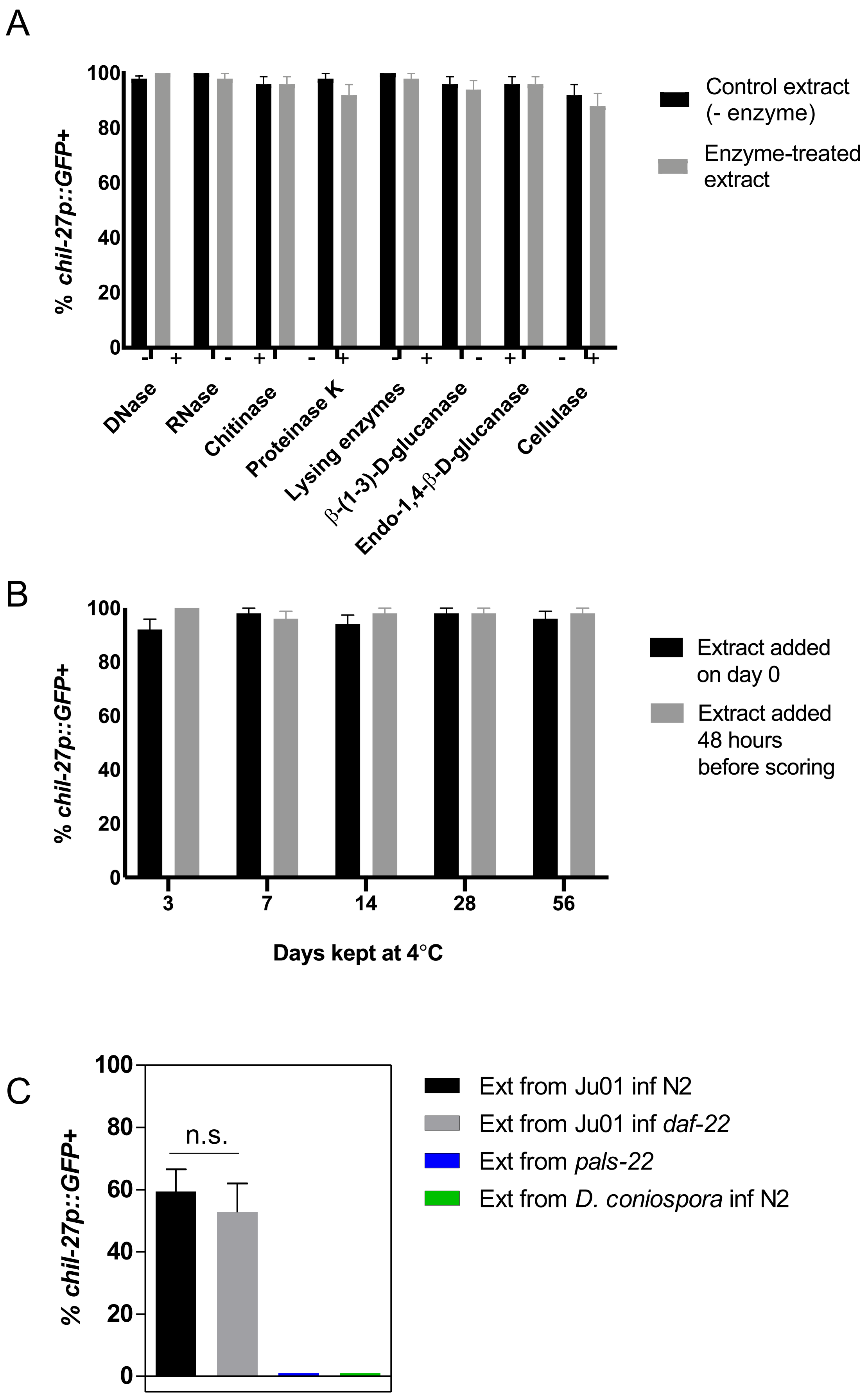
A
% chil-27p::GFP+
100
80
60
40
20
0
- + - + - + - + - + - + - + - +
DNase
RNase
Chitinase
Proteinase K
Lysing enzymes
β-(1-3)-D-glucanase
Endo-1,4-β-D-glucanase
Cellulase
Control extract (- enzyme)
Enzyme-treated extract
B
% chil-27p::GFP+
100
80
60
40
20
0
3
7
14
28
56
Days kept at 4°C
Extract added on day 0
Extract added 48 hours before scoring
C
% chil-27p::GFP+
100
80
60
40
20
0
n.s.
Ext from Ju01 inf N2
Ext from Ju01 inf daf-22
Ext from pals-22
Ext from D. coniospora inf N2

Figure S1

**Figure S1: Determining the chemical identity, stability and source of the active molecule in the pathogen extract. Related to Figure 1.**

(A) Treatment with various degrading enzymes does not reduce the potency of the extract. Each control extract was treated with the same conditions as the enzyme-treated extract, according to manufacturer's instructions to maximise enzyme activity. Lysing enzymes include DNase, RNAse, β-glucanase, cellulase, protease, and chitinase activities. The concentration used for DNase and RNase treatment was 100 μg/ml, while 1 mg/ml was used for rest of the enzymes. Enzyme-treated (+) and control extract (-) was added to the OP50 lawn and animals were scored 48 hours later for *chil-27p::GFP* induction (n = 50, for all treatments). (B) Incubation of extract on OP50 lawn at 4°C for up to 8 weeks does not decrease its potency to induce *chil-27p::GFP*. All plates were seeded with extract on day 0 and kept at 4°C until ready for testing. The same batch of extract was used as control and was added to the OP50 lawn 48 hours before animals were scored for *chil-27p::GFP* induction (n = 50, for all treatments). (C) Response to small-scale extracts (Ext) prepared from *M. humicola*-infected N2, *daf-22(m130)*, *D. coniospora*-infected N2 and uninfected *pals-22(icb89)* animals. No response was seen for *pals-22(-)* and *D. coniospora* and comparable response was found between N2 and *daf-22(-)* mutants. Error bars in A-C indicate standard error of the proportion.

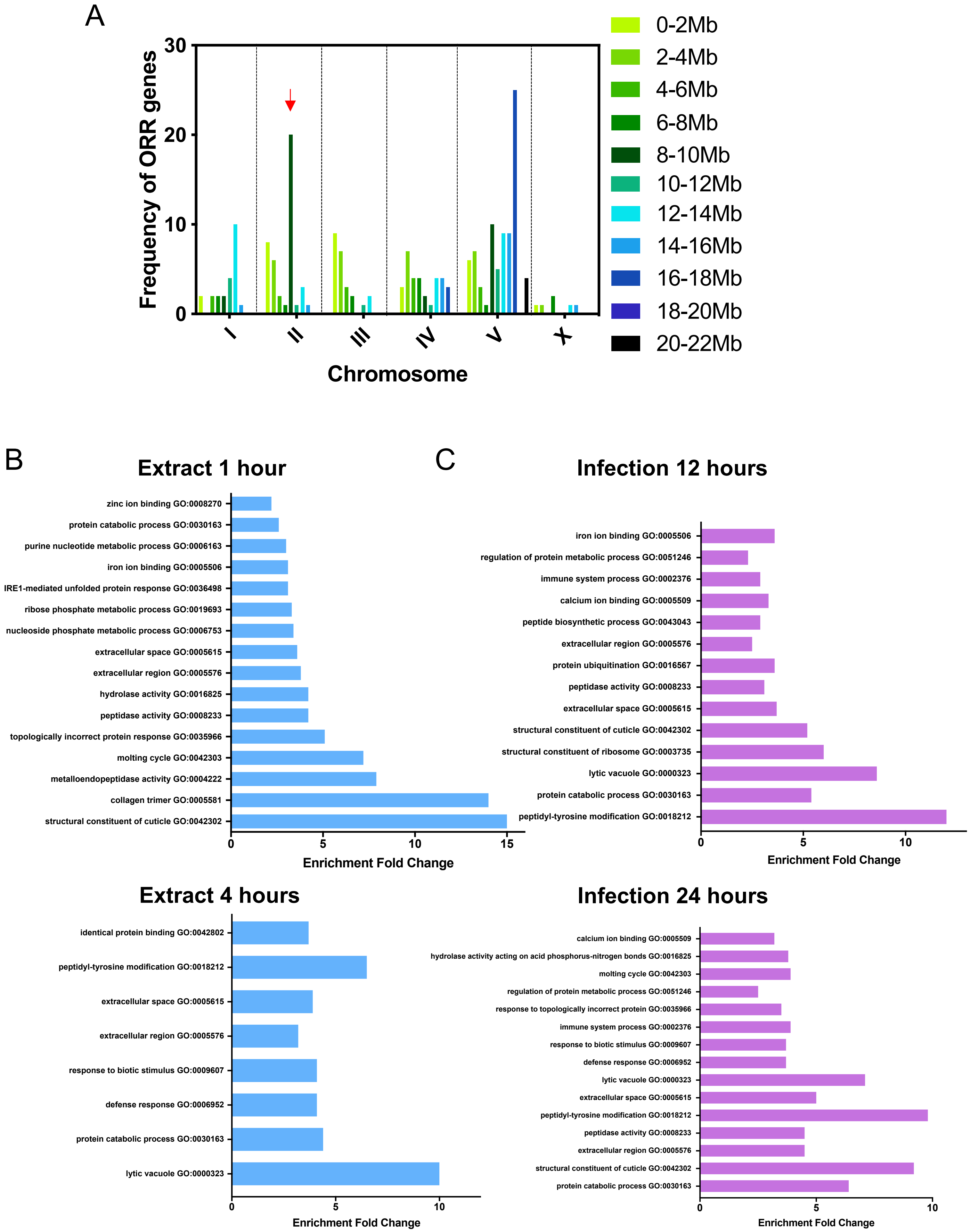
A
Frequency of ORR genes
30
20
10
0
I
II
III
IV
V
X
Chromosome
0-2Mb
2-4Mb
4-6Mb
6-8Mb
8-10Mb
10-12Mb
12-14Mb
14-16Mb
16-18Mb
18-20Mb
20-22Mb
B
Extract 1 hour
zinc ion binding GO:0008270
protein catabolic process GO:0030163
purine nucleotide metabolic process GO:0006163
iron ion binding GO:0005506
IRE1-mediated unfolded protein response GO:0036498
ribose phosphate metabolic process GO:0019693
nucleoside phosphate metabolic process GO:0006753
extracellular space GO:0005615
extracellular region GO:0005576
hydrolase activity GO:0016825
peptidase activity GO:0008233
topologically incorrect protein response GO:0035966
molting cycle GO:0042303
metalloendopeptidase activity GO:0004222
collagen trimer GO:0005581
structural constituent of cuticle GO:0042302
0
5
10
15
Enrichment Fold Change
C
Infection 12 hours
iron ion binding GO:0005506
regulation of protein metabolic process GO:0051246
immune system process GO:0002376
calcium ion binding GO:0005509
peptide biosynthetic process GO:0043043
extracellular region GO:0005576
protein ubiquitination GO:0016567
peptidase activity GO:0008233
extracellular space GO:0005615
structural constituent of cuticle GO:0042302
structural constituent of ribosome GO:0003735
lytic vacuole GO:0000323
protein catabolic process GO:0030163
peptidyl-tyrosine modification GO:0018212
0
5
10
Enrichment Fold Change
Extract 4 hours
identical protein binding GO:0042802
peptidyl-tyrosine modification GO:0018212
extracellular space GO:0005615
extracellular region GO:0005576
response to biotic stimulus GO:0009607
defense response GO:0006952
protein catabolic process GO:0030163
lytic vacuole GO:0000323
0
5
10
Enrichment Fold Change
Infection 24 hours
calcium ion binding GO:0005509
hydrolase activity acting on acid phosphorus-nitrogen bonds GO:0016825
molting cycle GO:0042303
regulation of protein metabolic process GO:0051246
response to topologically incorrect protein GO:0035966
immune system process GO:0002376
response to biotic stimulus GO:0009607
defense response GO:0006952
lytic vacuole GO:0000323
extracellular space GO:0005615
peptidyl-tyrosine modification GO:0018212
peptidase activity GO:0008233
extracellular region GO:0005576
structural constituent of cuticle GO:0042302
protein catabolic process GO:0030163
0
5
10
Enrichment Fold Change

Figure S2

**Figure S2: Distribution and gene ontology enrichment analysis of upregulated ORR genes. Related to Figure 2.**

(A) Graph showing induced ORR genes per chromosome in bins of 2Mb. Genes on chromosome V are significantly overrepresented with a binomial test (*p* value<0.001). Local peaks in the frequency of induced genes along the chromosomes may correspond to response clusters, for example the most prominent peak (indicated by a red arrow) on chromosome II (8-10 Mb bin) includes many *chil* genes. (B-C) Gene ontology enrichment analysis of upregulated genes identified at different timepoints. Two time points are shown for pathogen extract treatment (B) and infection (C). All datasets have been analysed using the enrichment analysis tool on Wormbase.

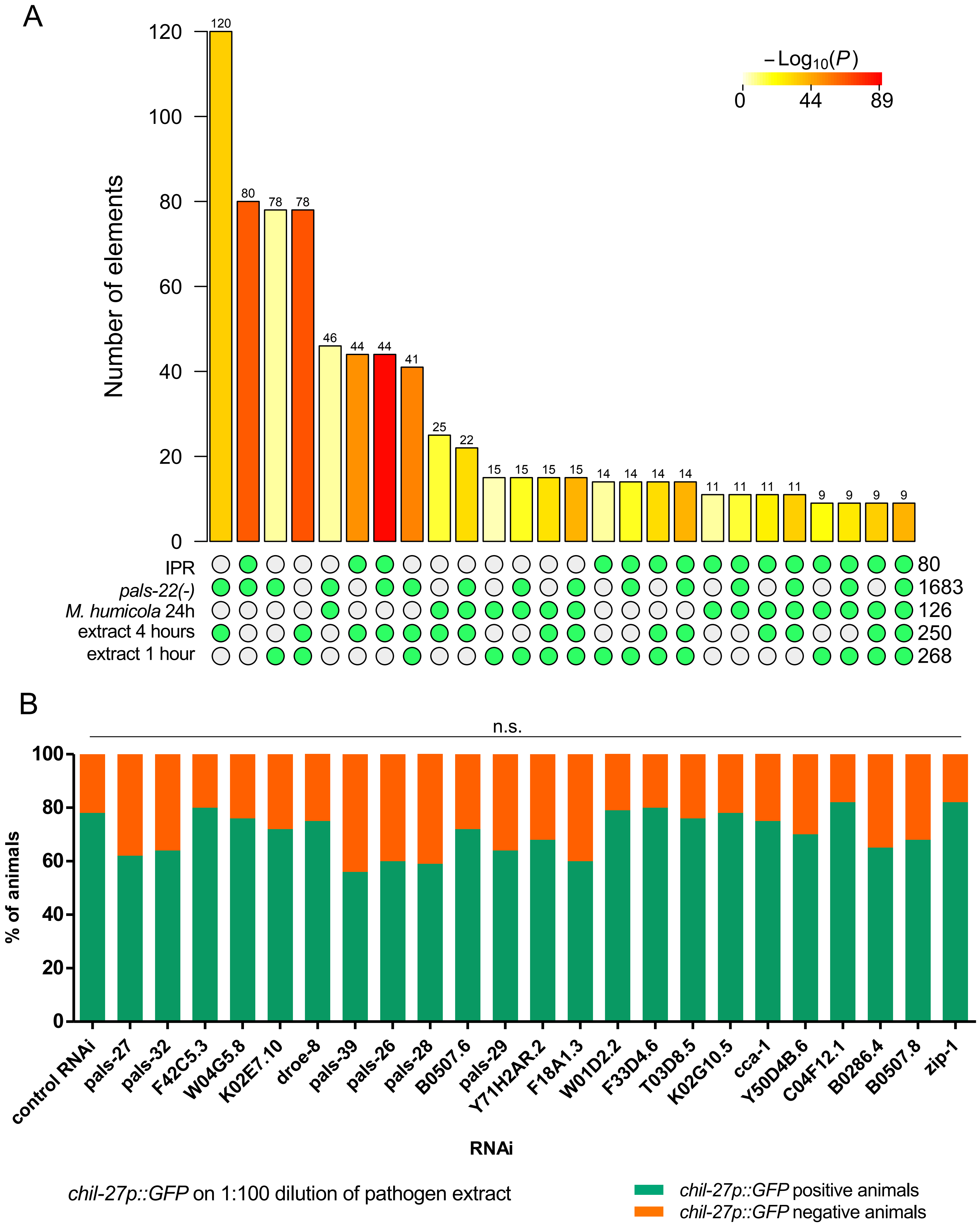
A
Number of elements
−Log10(P)
0
44
89
IPR
pals-22(-)
M. humicola 24h
extract 4 hours
extract 1 hour
80
1683
126
250
268
B
n.s.
% of animals
control RNAi
pals-27
pals-32
F42C5.3
W04G5.8
K02E7.10
droe-8
pals-39
pals-26
pals-28
B0507.6
pals-29
Y71H2AR.2
F18A1.3
W01D2.2
F33D4.6
T03D8.5
K02G10.5
cca-1
Y50D4B.6
C04F12.1
B0286.4
B0507.8
zip-1
RNAi
chil-27p::GFP on 1:100 dilution of pathogen extract
chil-27p::GFP positive animals
chil-27p::GFP negative animals

Figure S3

**Figure S3: Analysis of the transcriptional response to pathogen extract exposure. Related to Figure 3.**

(A) Overlap between different gene sets included in the GSEA analysis. Bars with numbers on top show the overlap of specific gene sets depicted in green. The size of each gene set appears on the bottom right. Key shows *p* value based on Fisher's exact test (Wang et al., 2015). (B) RNAi screen targeting ORR genes and studying their impact on *chil-27p::GFP* induction. No significant changes were found.

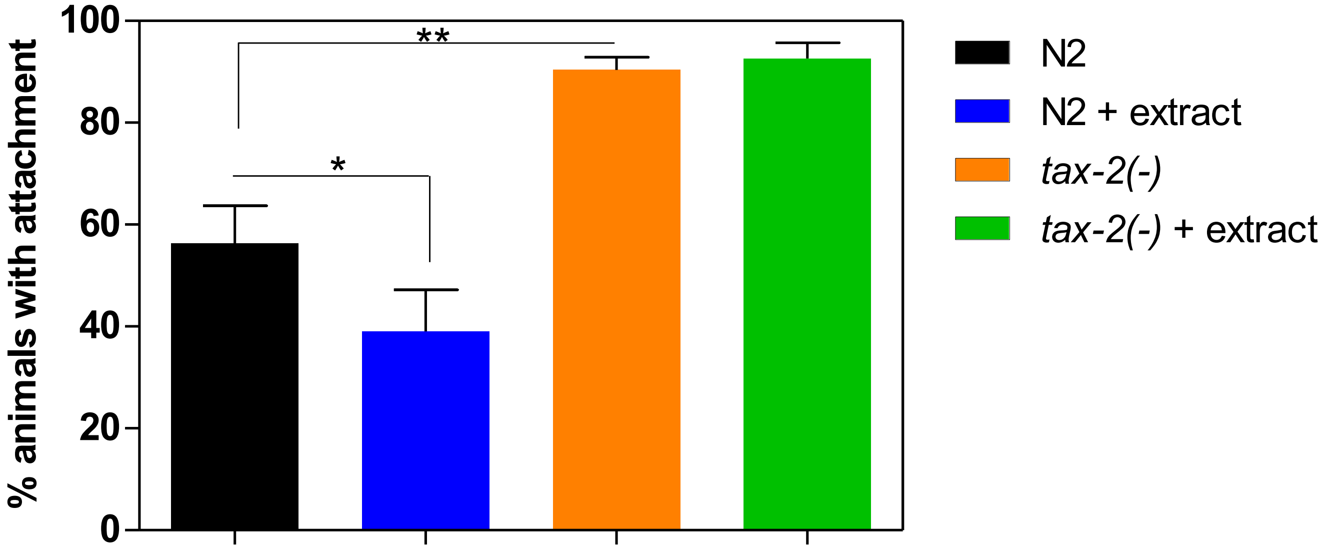
% animals with attachment
100
80
60
40
20
0
**
*
N2
N2 + extract
tax-2(-)
tax-2(-) + extract

Figure S4

**Figure S4: Pathogen attachment assay for *tax-2(p691)* mutant animals. Related to Figure 5.**

(A) Percentage of N2 and *tax-2(p691)* animals with and without pretreatment with extract showing oomycete attachment after 4h exposure with the pathogen at 20$^{o}$C (** *p*<0.01 and * *p*<0.05 with Chi-square test, n=50).

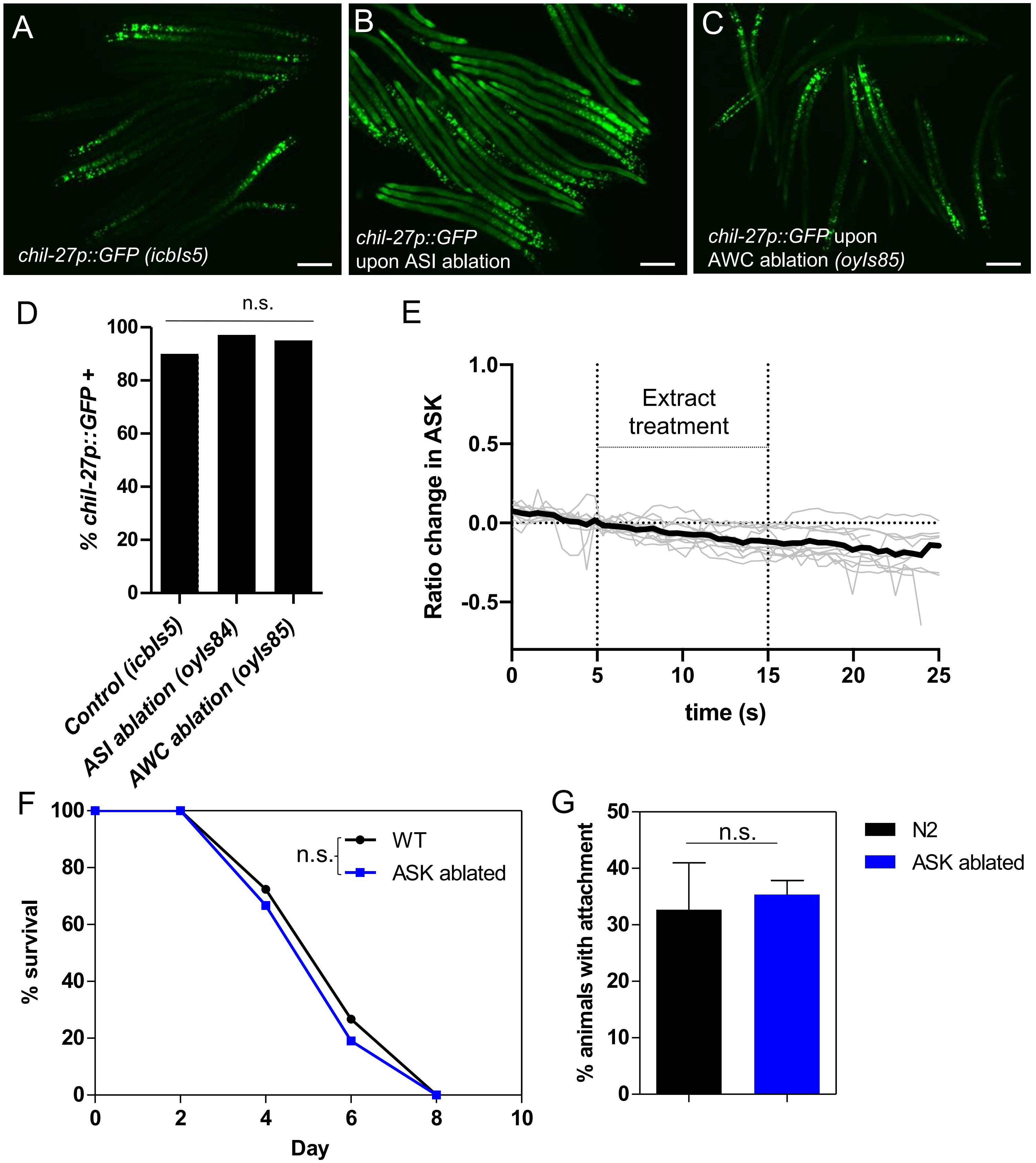

A
chil-27p::GFP (icbIs5)
B
chil-27p::GFP
upon ASI ablation
C
chil-27p::GFP upon
AWC ablation (oyIs85)
D
n.s.
% chil-27p::GFP +
0
20
40
60
80
100
Control (icbIs5)
ASI ablation (oyIs84)
AWC ablation (oyIs85)
E
Ratio change in ASK
Extract
treatment
1.0
0.5
0.0
-0.5
0
5
10
15
20
25
time (s)
F
% survival
n.s.
WT
ASK ablated
0
20
40
60
80
100
0
2
4
6
8
10
Day
G
% animals with attachment
n.s.
N2
ASK ablated
0
10
20
30
40
50


Figure S5

**Figure S5: Calcium response in ASK upon oomycete extract treatment and response to extract and infection in animals with specific neurons ablated. Related to Figure 5.**

(A-D) Response to oomycete extract in not impaired in strains with ASI or AWC neurons ablated. Induction assay showing *chil-27p::GFP* expression in control carrying the transgene (A), ASI ablated *(oyIs84)* (B) and AWC ablated *(oyIs85)* strains (C) in response to treatment with 1:100 dilution of oomycete extract. Neuron ablation is performed in these strains by driving split caspase expression under *gpa-4/gcy-27* promoters in (B) and a *ceh-36* promoter in (C). Scale bar is 200 µm. (D) Quantification of the *chil-27p::GFP* induction in response to extract treatment (n>30). (E) Calcium traces (n=12) over time and upon delivery of pathogen extract in ASK neurons using transgene *ljEx1186[sra-9p::GCaMP3::SL2-tagRFP]*. GCaMP3 and tagRFP intensities were measured as the mean pixel intensity of the 100 brightest pixels in a circular region of interest (ROI) with a 14 pixel radius. Calcium traces were computed as the change in the GCaMP3/tagRFP ratio (R) from the baseline value defined as the mean R prior to stimulus onset. Note no increase in R upon exposure to extract from time 5-15sec. (F) Survival curve of N2 and ASK ablated animals *(qrIs2)* at 20$^{o}$C in the presence of *M. humicola* JUo1 (n.s., *p*>0.05, log-rank test, n=60 per condition). (G) Percentage of N2 and ASK ablated animals showing oomycete attachment after 4h exposure with the pathogen at 20$^{o}$C (n.s., *p*>0.05, Chi-square test, n=40).
